# Synthesis of indole–cycloalkyl[*b*]pyridine hybrids via a four-component six-step tandem process

**DOI:** 10.3762/bjoc.14.269

**Published:** 2018-11-22

**Authors:** Muthumani Muthu, Rakkappan Vishnu Priya, Abdulrahman I Almansour, Raju Suresh Kumar, Raju Ranjith Kumar

**Affiliations:** 1 Department of Organic Chemistry, School of Chemistry, Madurai Kamaraj University, Madurai 625 021, Tamil Nadu, India; 2 Department of Physics, Madura College, Madurai 625011, Tamil Nadu, India; 3 Department of Chemistry, College of Science, King Saud University, Riyadh 11451, Saudi Arabia

**Keywords:** cycloalkyl[*b*]pyridine-3-carbonitrile, cyclododecanone, 3-(1*H*-indol-3-yl)-3-oxopropanenitrile, tandem reaction

## Abstract

The one-pot four-component reaction of 3-(1*H*-indol-3-yl)-3-oxopropanenitriles, aromatic aldehydes, cycloalkanones and ammonium acetate occurred via a six-step tandem Knoevenagel condensation–nucleophilic addition to carbonyl–Michael addition–N-cyclization–elimination–air oxidation sequence to afford structurally intriguing indole–cycloalkyl[*b*]pyridine-3-carbonitrile hybrid heterocycles in excellent yields.

## Introduction

The syntheses of novel heterocycles through greener protocols have received a great deal of attention of the synthetic organic chemists in view of environmental concerns [1–3]. The multicomponent tandem/domino reaction is one among several green chemical protocols widely employed for the synthesis of myriad of natural products and hybrid heterocycles [4–13]. These reactions are one-pot processes involving several bond forming steps under identical reaction conditions affording the desired product in a single transformation [14–22]. Hence, multicomponent tandem reactions minimize the number of steps to synthesize complex heterocycles, avoid the isolation and purification of the intermediates, allow less waste to the environment, shorten the reaction time and are also cost effective.

**Figure 1:**
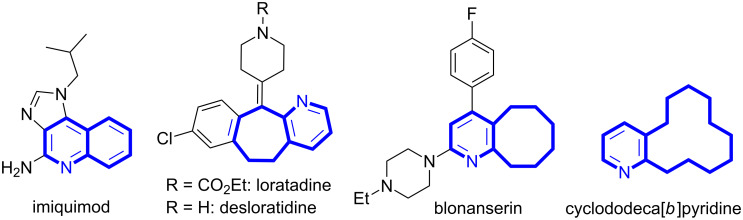
Examples of biologically important cycloalkyl-fused pyridines.

Carbocyclic or heterocyclic fused pyridine derivatives are an important class of compounds omnipresent in natural products and biologically relevant synthetic compounds [23–27]. For example, imiquimod is an immune response modifier used to treat warts on the skin and certain type of skin cancer called superficial basal cell carcinoma (Figure 1) [28–29]. Loratadine is a second-generation histamine H1 receptor antagonist used to treat allergic rhinitis and urticarial [30–31]. Blonanserin is an atypical antipsychotic drug used to treat schizophrenia [32], whereas the decahydrocyclododeca[*b*]pyridine has been reported as inhibitors of cytochrome P450 [33]. Furthermore, muscopyridine is being used in perfume industry [34].

Among the several methods available for the synthesis of pyridines or cycloalkyl-fused pyridines [23–27,35–44], the one-pot four-component reaction of cyclic/acyclic ketones, malononitrile, aromatic aldehyde and ammonium acetate affording 2-amino-3-cyanopyridine derivatives have been explored extensively [45–55]. However, syntheses of pyridine scaffolds bearing an indole side chain have received less attention. For instance, the multicomponent reactions of aldehydes, 3-(1*H*-indol-3-yl)-3-oxopropanenitriles and 5-aminopyrazol or naphthylamine afforded indole substituted fused pyridine derivatives [56]. 2-Indole substituted pyridine derivatives have also been prepared through AlCl_3_-induced C–C bond forming reaction [57] and three-component reactions of aromatic aldehydes, 3-(1*H*-indol-3-yl)-3-oxopropanenitrile and malononitrile [58–59], 2-acetylpyridine [60] or 3-amino-2-enones [61]. Incidentally, the indole scaffold is found in several natural products and bioactive synthetic compounds [62–67]. For example, the synthetic drug sumatriptan used for the treatment of migraine and cluster headaches belongs to the triptan class, whereas indomethacin is a non-steroidal anti-inflammatory drug used to relieve pain, swelling and joint stiffness caused by arthritis [68–69].

Recently we reported the synthesis of pyridine/benzo-fused cyclododecanes through a four-component tandem reaction [70]. In continuation we herein report the synthesis of novel indole substituted cycloalkyl[*b*]pyridine-3-carbonitriles from a one-pot six-step tandem protocol involving 3-(1*H*-indol-3-yl)-3-oxopropanenitriles, aromatic aldehydes, cycloalkanones and ammonium acetate. This work also stems from our continuous effort in synthesizing novel cycloalkyl[*b*]pyridine-3-carbonitrile hybrid heterocycles via tandem/domino reaction [71–72].

## Results and Discussion

**Scheme 1:**
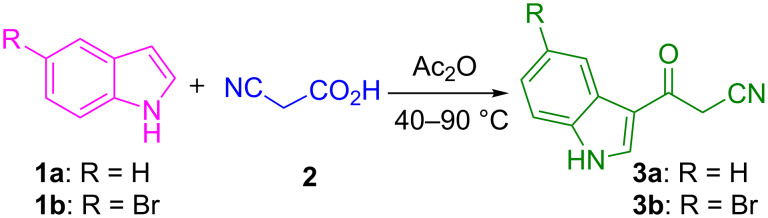
Synthesis of 3-oxopropanenitriles **3**.

Initially the precursors viz. 3-(1*H*-indol-3-yl)-3-oxopropanenitriles **3** were synthesized from the reaction of indoles **1** and 2-cyanoacetic acid (**2**) in acetic anhydride under heating conditions (Scheme 1) [73].

Subsequently the one-pot four-component reaction of 3-(1*H*-indol-3-yl)-3-oxopropanenitrile (**3a**), 4-chlorobenzaldehyde (**4f**), cyclododecanone (**5a**) and ammonium acetate (**6**) was chosen as a model in order to identify the optimum conditions for this reaction (Table 1). To begin with, a 1:1:1:2 mixture of the above reactants was refluxed in toluene for 4 h which led to the formation of indole–cyclododeca[*b*]pyridine-3-carbonitrile **7f** and the intermediate (*E*)-3-(4-chlorophenyl)-2-(1*H*-indole-3-carbonyl)acrylonitrile (**8**) in 10 and 80% yields, respectively. Intermediate **8** was formed exclusively when the reaction was carried out in refluxing acetonitrile or isopropanol. The yield of **7f** increased up to 60% in refluxing methanol. However, the same reaction in refluxing ethanol afforded solely the desired product **7f** in 93% yield within 2 h (Table 1). Furthermore, after completion of the reaction as evident from the TLC, the mixture was set aside for 6 h and the resultant precipitate was filtered, washed with ethanol and dried under vacuum to obtain pure **7f** without the need for additional purification methods.

**Table 1:** Synthesis of indole–cyclododeca[*b*]pyridine-3-carbonitrile **7f**.

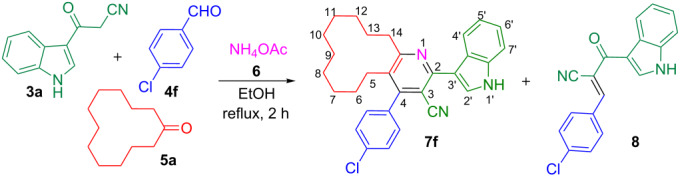			

entry	conditions	yield (%)^a^	

		**7f**	**8**

1	toluene, reflux, 4 h	10	80
2	CH_3_CN, reflux, 3 h	–	96
3	iPrOH, reflux, 3 h	–	98
4	MeOH, reflux, 6 h	60	21
5	EtOH, reflux, 2 h	93	–

^a^Isolated yield.

The structure of **7f** was elucidated with the help of one- and two-dimensional NMR spectroscopy. In the ^1^H NMR of **7f** the 5- and 14-CH_2_ protons appeared as triplets at 2.56 and 3.01 ppm (*J* = 9.0 Hz), respectively. From the H,H-COSY correlation of 14-CH_2_ protons, the multiplets in the range 2.03–2.18 ppm was assigned to the 13-CH_2_ protons. The CH_2_ protons of C-6 to C-12 appeared as multiplets in the range 1.27–1.58 ppm. The H,H-COSY spectrum revealed that the NH proton of indole ring, which appeared as a broad singlet at 8.58 ppm is coupled to a doublet at 8.16 ppm (*J* = 3.0 Hz) due to 2′-CH proton.

**Scheme 2:**
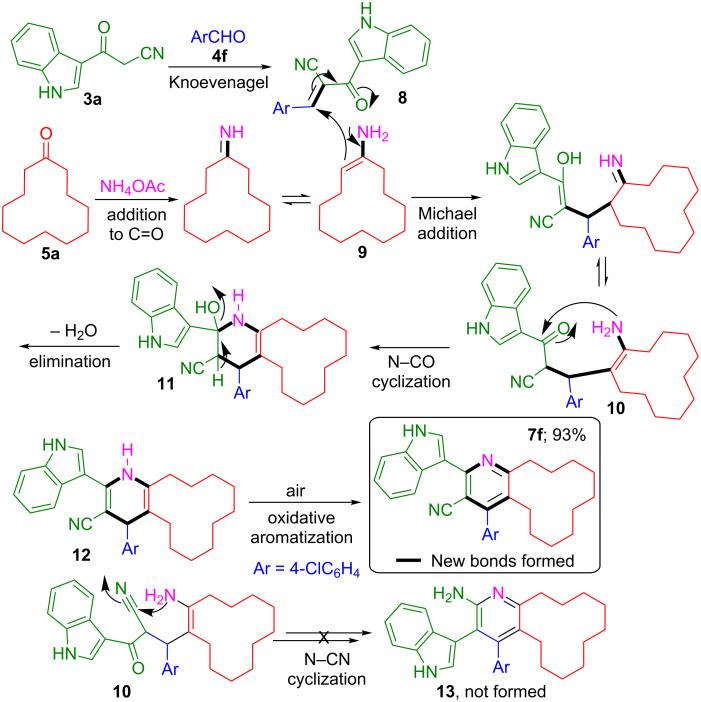
Proposed mechanism for the formation of **7f**.

A persuasive mechanism to justify the formation of indole–cyclododeca[*b*]pyridine-3-carbonitrile hybrid **7f** is depicted in Scheme 2. Initially, the Knoevenagel condensation of 3-(1*H*-indol-3-yl)-3-oxopropanenitrile (**3a**) and 4-chlorobenzaldehyde (**4f**) leads to the formation of (*E*)-3-(4-chlorophenyl)-2-(1*H*-indole-3-carbonyl)acrylonitrile (**8**). Simultaneously, the reaction of cyclododecanone (**5a**) with ammonium acetate affords the enamine **9**. The Michael addition of **8** and the enamine **9** yields the intermediate **10**. Then the amino group of **10** undergoes intramolecular cyclization with the carbonyl to give **11**, which subsequently undergoes dehydration to yield the cyclododeca[*b*]pyridine-3-carbonitrile **12**. The intermediate **12** upon oxidative aromatization by molecular oxygen as the sole oxidant yields the indole–cyclododeca[*b*]pyridine-3-carbonitrile **7f**. This four-component multistep tandem reaction afforded **7f** in 93% yield involving the formation of two new C–N and C–C bonds in a single transformation without the need to isolate or purify the intermediates. Furthermore, the above reaction occurred stereoselectively to afford indole–cyclododeca[*b*]pyridine-3-carbonitrile **7f** exclusively, which is evident from the fact that the decahydrocyclododeca[*b*]pyridin-2-amine **13** anticipated through the intramolecular cyclization of the amino group and the CN in intermediate **10** was not formed in the reaction (Scheme 2).

**Scheme 3:**
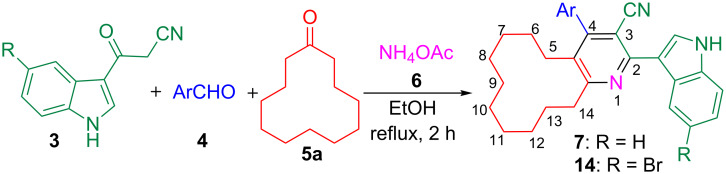
Synthesis of indole–cyclododeca[*b*]pyridine-3-carbonitriles **7** and **14**.

This one-pot four-component strategy was then employed to synthesize twenty-three novel indole–cyclododeca[*b*]pyridine-3-carbonitrile hybrid heterocycles **7** by varying the 3-(1*H*-indol-3-yl)-3-oxopropanenitrile **3** and aromatic aldehyde **4** (Scheme 3 and Table 2). In all the cases, the reaction occurred smoothly affording excellent yields of the product **7** (85–95%). However, the reaction failed to occur with aliphatic aldehydes viz. formaldehyde, heptanal, pentanal and hexanal. The structure of all the indole–cyclododeca[*b*]pyridine-3-carbonitrile hybrid heterocycles **7** was elucidated by NMR spectroscopy.

**Table 2:** Yields and melting points of **7** and **14**.

entry	comp	Ar	R	yield (%)^a^	mp (°C)

1	**7a**	C_6_H_5_	H	90	165–166
2	**7b**	4-CH_3_C_6_H_4_	H	93	153–154
3	**7c**	4-CH_3_OC_6_H_4_	H	85	216–217
4	**7d**	4-iPrC_6_H_4_	H	91	202–203
5	**7e**	4-FC_6_H_4_	H	92	198–199
6	**7f**	4-ClC_6_H_4_	H	93	241–242
7	**7h**	4-CNC_6_H_4_	H	85	214–215
8	**7i**	4-O_2_NC_6_H_4_	H	95	212–213
9	**7j**	2-CH_3_C_6_H_4_	H	94	268–269
10	**7l**	2-BrC_6_H_4_	H	92	254–255
11	**7m**	3-O_2_NC_6_H_4_	H	94	214–215
12	**7n**	2,4-Cl_2_C_6_H_3_	H	93	232–234
13	**7s**	3,4-(OCH_3_)_2_C_6_H_3_	H	91	228-229
14	**7t**	3,4,5-(OCH_3_)_3_C_6_H_2_	H	92	199–200
15	**7u**	thiophene-2-yl	H	90	206–207
16	**14b**	4-CH_3_C_6_H_4_	Br	92	272–273
17	**14d**	4-iPrC_6_H_4_	Br	90	280–281
18	**14f**	4-ClC_6_H_4_	Br	95	289–290
19	**14g**	4-BrC_6_H_4_	Br	92	299–300
20	**14m**	3-O_2_NC_6_H_4_	Br	95	297–298
21	**14p**	2-F,4-ClC_6_H_3_	Br	89	304–305
22	**14s**	3,4-(OCH_3_)_2_C_6_H_3_	Br	90	294–295
23	**14t**	3,4,5-(OCH_3_)_3_C_6_H_2_	Br	94	264–265

^a^Isolated yield.

**Figure 2:**
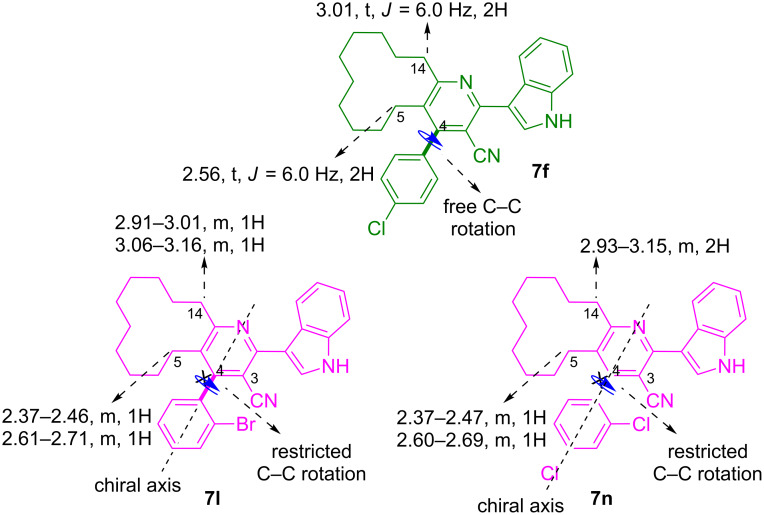
Axial chirality due to restricted C–C bond rotation (representative cases).

Furthermore, the analysis of ^1^H NMR spectra revealed that the indole–cyclododeca[*b*]pyridine-3-carbonitriles **7** with *ortho/ortho-para/ortho-meta* substituted phenyl ring at C-4, exhibited axial chirality. For instance, in the case of **7f** with *p*-Cl substituted phenyl ring at C-4, the 5- and 14-CH_2_ protons appeared as triplets at 2.56 and 3.01 ppm, respectively. However, in **7l** wherein C-4 is bearing an *o*-Br substituted phenyl, the 5-CH_2_ protons appeared as multiplets in the range of 2.37–2.46 and 2.61–2.71 ppm, whereas the 14-CH_2_ protons appeared as multiplets in the range of 2.91–3.01 and 3.06–3.16 ppm. The diastereotopic behavior of 5- and 14-CH_2_ protons of indole–cyclododeca[*b*]pyridine-3-carbonitrile hybrid heterocycles **7** with an *ortho/ortho-para/ortho-meta* substituted phenyl ring at C-4 may be attributed to the axial chirality induced in these molecules due to the restricted rotation of the C–C single bond. The steric hindrance exerted between the nitrile group at C-3 and the *ortho/ortho-para/ortho-meta* substitution in the phenyl ring at C-4 restricts the free rotation of the C-4–phenyl C–C single bond thereby inducing axial chirality in these molecules (representative examples, Figure 2).

**Figure 3:**
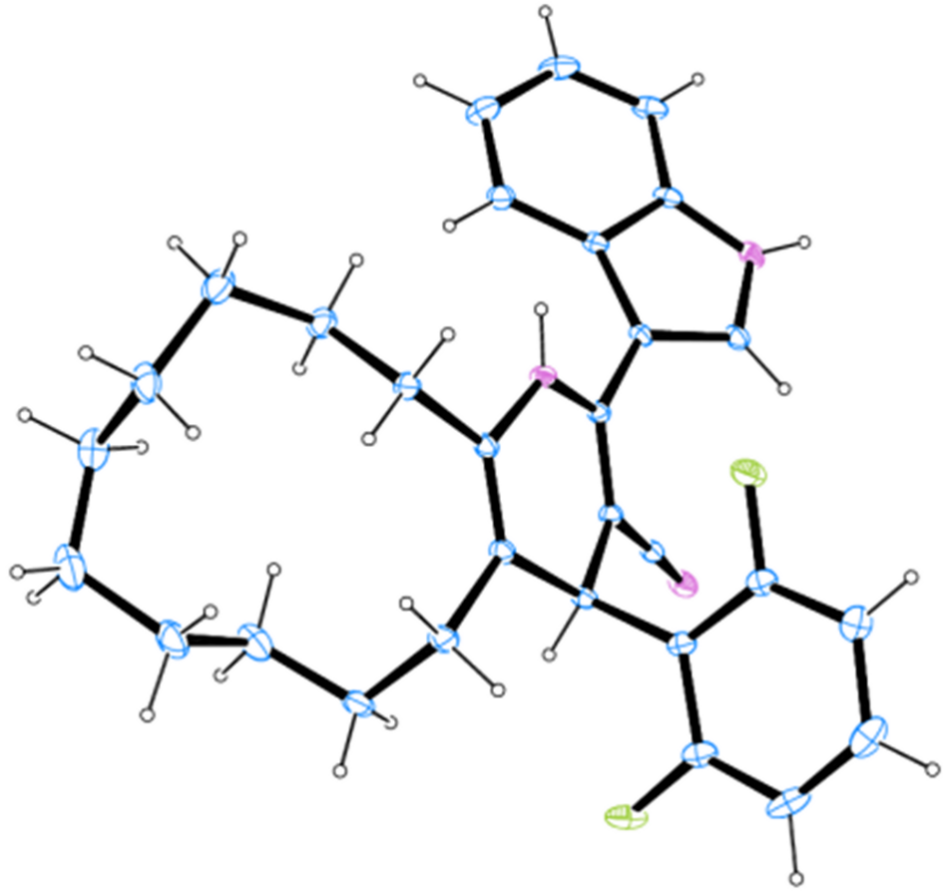
ORTEP diagram of **12r**.

Interestingly in some cases this reaction afforded the unaromatized indole–cyclododeca[*b*]pyridine-3-carbonitriles **12** (Table 3). These experiments were repeated thrice in order to ascertain the exclusive formation of **12**. The structure of **12** was confirmed from the ^1^H NMR spectra, wherein the characteristic singlet around 4.6–5.1 ppm due to the 4-CH proton was observed. In addition, in the case of **12r** the structure was confirmed from the single crystal X-ray studies (Figure 3) [74]. A careful analysis of the reaction progress revealed that in these reactions the corresponding product **12** precipitated from the reaction mixture within 2 h of reflux (Table 3), which was also an indication of the completion of the reaction. Further increment in the reaction time had no influence on the reaction to afford the aromatized product **7**. However, in other cases (Table 2) the reaction was complete within 2 h (TLC analysis) but the product **7** precipitated after 6–8 h.

**Table 3:** Synthesis of indole–cyclododeca[*b*]pyridine-3-carbonitriles **12**.

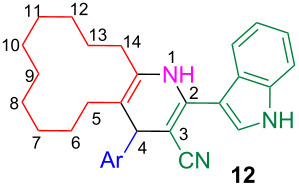				

entry	comp	Ar	yield (%)^a^	mp (°C)

1	**12g**	4-BrC_6_H_4_	89	222–223
2	**12k**	2-ClC_6_H_4_	95	265–266
3	**12o**	2-Cl,3-CH_3_OC_6_H_3_	92	221–222
4	**12p**	2-F,4-ClC_6_H_3_	87	264–265
5	**12q**	2,5-(OCH_3_)_2_C_6_H_3_	85	224–225
6	**12r**	2,6-F_2_C_6_H_3_	89	269–270

^a^Isolated yield.

**Scheme 4:**
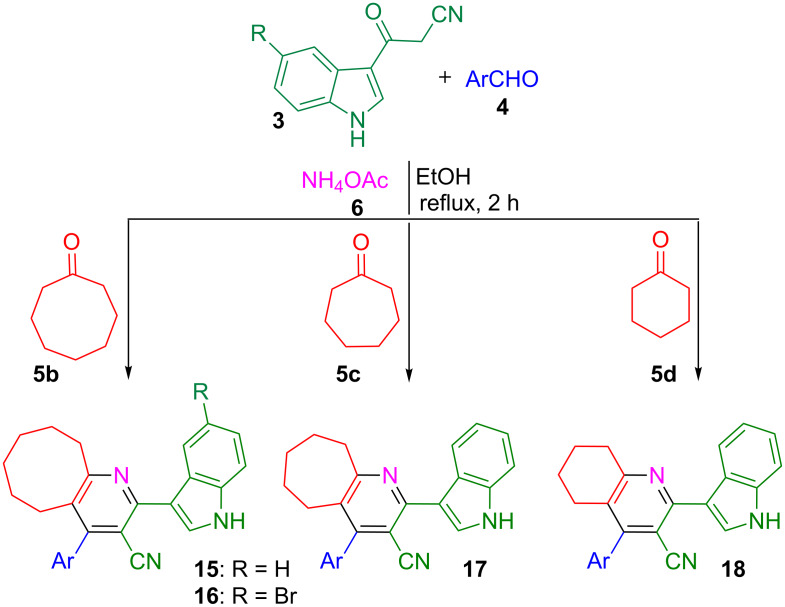
Synthesis of indole–cycloalkyl[*b*]pyridine-3-carbonitrile hybrids **15**–**18**.

**Figure 4:**
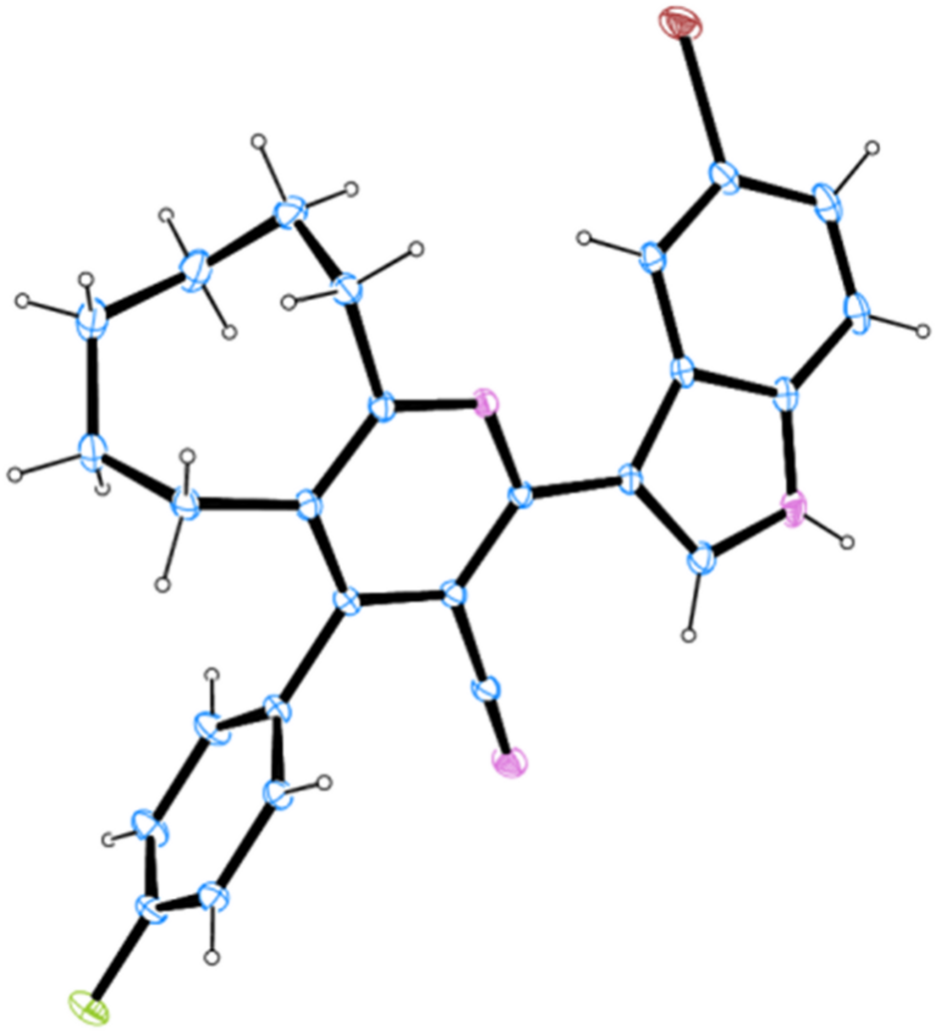
ORTEP diagram of **16f**.

Gratified by the above results and, also to demonstrate the general applicability of this protocol, the reaction of 3-(1*H*-indol-3-yl)-3-oxopropanenitriles **3**, aromatic aldehydes **4** and ammonium acetate (**6**) with lower ring-size cycloalkanones, viz. cyclooctanone (**5b**), cycloheptanone (**5c**) and cyclohexanone (**5d**) was investigated (Scheme 4). Under the previously established conditions, the reaction led to the formation of the respective cycloalkane-fused pyridine–indole hybrid heterocycles in excellent yields (80–95%). However, the reaction failed to occur with cyclopentanone. In total thirty-five indole–cycloalkyl[*b*]pyridine-3-carbonitrile hybrids **15**–**18** were isolated (Table 4). The structure of all the hybrid heterocycles **15**–**18** was elucidated using NMR spectroscopy and in the case of **16f** the structure was further confirmed from single crystal X-ray studies (Figure 4) [74].

**Table 4:** Yields and melting points of **15**–**18**.

entry	comp	Ar	R	yield (%)^a^	mp (°C)

1	**15a**	C_6_H_5_	H	92	189–190
2	**15b**	4-CH_3_C_6_H_4_	H	93	201–202
3	**15d**	4-iPrC_6_H_4_	H	92	198–199
4	**15e**	4-FC_6_H_4_	H	94	225–226
5	**15f**	4-ClC_6_H_4_	H	90	214–215
6	**15g**	4-BrC_6_H_4_	H	91	234–235
7	**15h**	4-CNC_6_H_4_	H	82	235–236
8	**15i**	4-O_2_NC_6_H_4_	H	92	245–246
9	**15j**^b^	2-CH_3_C_6_H_4_	H	91	222–223
10	**15l^b^**	2-BrC_6_H_4_	H	94	259–260
11	**15m**	3-O_2_NC_6_H_4_	H	92	236–237
12	**15n**	2,4-Cl_2_C_6_H_3_	H	85	254–255
13	**15p**	2-F,4-ClC_6_H_3_	H	94	237–238
14	**15r**^b^	2,6-F_2_C_6_H_3_	H	92	261–262
15	**15s**	3,4-(OCH_3_)_2_C_6_H_3_	H	92	267–268
16	**15t**	3,4,5-(OCH_3_)_3_C_6_H_2_	H	95	198–199
17	**15u**	thiophene-2-yl	H	94	200–201
18	**16a**	C_6_H_5_	Br	88	186–187
19	**16b**	4-CH_3_C_6_H_4_	Br	89	268–269
20	**16c**	4-CH_3_OC_6_H_4_	Br	89	274–275
21	**16d**	4-iPrC_6_H_4_	Br	90	276–277
22	**16e**	4-FC_6_H_4_	Br	92	289–290
23	**16f**	4-ClC_6_H_4_	Br	91	278–279
24	**16g**	4-BrC_6_H_4_	Br	95	288–289
25	**16o**^b^	2-Cl,3-CH_3_OC_6_H_3_	Br	90	279–280
26	**16p**	2-F,4-ClC_6_H_3_	Br	90	297–298
27	**16t**	3,4,5-(OCH_3_)_3_C_6_H_2_	Br	95	259–260
28	**17b**	4-CH_3_C_6_H_4_	H	80	165–166
29	**17f**	4-ClC_6_H_4_	H	82	184–185
30	**17l**^b^	2-BrC_6_H_4_	H	89	210–211
31	**17v**	4-CH_3_SC_6_H_4_	H	84	170–171
32	**18a**	C_6_H_5_	H	81	174–175
33	**18b**	4-CH_3_C_6_H_4_	H	80	164–165
34	**18f**	4-ClC_6_H_4_	H	80	158–159
35	**18v**	4-CH_3_SC_6_H_4_	H	85	162–163

^a^Yield of isolated product. ^b^The unaromatized product was obtained.

## Conclusion

The syntheses of a library of novel indole–cycloalkyl[*b*]pyridine-3-carbonitrile hybrid heterocycles have been achieved through a facile one-pot four-component strategy. This reaction occurred through a six-step tandem Hantzsch-like process involving Knoevenagel–Michael–nucleophilic addition–intramolecular cyclization–elimination–oxidative aromatization sequence of reactions in a single transformation leading to the formation of two new C–N and C–C bonds. The structure of all the indole–cycloalkyl[*b*]pyridine-3-carbonitrile hybrid heterocycles was elucidated with the help of NMR spectroscopy and supported by single crystal X-ray studies for two compounds. The indole–cycloalkyl[*b*]pyridine-3-carbonitriles comprising *ortho/ortho-para/ortho-meta* substituted phenyl rings exhibited axial chirality due to restricted C–C single bond rotation.

## Supporting Information

File 1 Experimental procedure, compound characterization data and copies of NMR spectra.
