# Supplementary material for: Synthesis of indole–cycloalkyl[b]pyridine hybrids via a four-component six-step tandem process
Source: Beilstein J Org Chem. 2018 Nov 22;14:2907–15. doi: 10.3762/bjoc.14.269 (PMC6278771; doi:10.3762/bjoc.14.269)
Supplement: File 1 — Experimental procedure, compound characterization data and copies of NMR spectra. [file Beilstein_J_Org_Chem-14-2907-s001.pdf]

BEILSTEIN JOURNAL OF ORGANIC CHEMISTRY

Supporting Information

for

Synthesis of indole–cycloalkyl[*b*]pyridine hybrids via a four-component six-step tandem process

Muthumani Muthu, Rakkappan Vishnu Priya, Abdulrahman I. Almansour,
Raju Suresh Kumar and Raju Ranjith Kumar

Beilstein J. Org. Chem. **2018,** *14,* 2907–2915. doi:10.3762/bjoc.14.269

Experimental procedure, compound characterization data and copies of NMR spectra

License and Terms: This is a supporting information file under the terms of the Creative Commons Attribution License (http://creativecommons.org/licenses/by/4.0). Please note that the reuse, redistribution and reproduction in particular requires that the authors and source are credited.
The license is subject to the *Beilstein Journal of Organic Chemistry* terms and conditions: (https://www.beilstein-journals.org/bjoc)

S. No.	Content	Page No.
1	Experimental section – General	S2
2	General procedure for the synthesis of indole–cycloalkyl[*b*]pyridine-3-carbonitriles **7**, **12** and **14–18**	S3
3	Compounds characterization data	S3
4	Copies of NMR spectra	S30
5	X-ray Structure Determination and Refinement	S94
6	**Table S1**. Crystal data and structure refinement parameters of **12r**	S95
7	**Table S2**. Crystal data and structure refinement parameters of **16f**	S96

Experimental section

General

Melting point of the indole–cycloalkyl[*b*]pyridine-3-carbonitrile hybrids were measured in open capillary tubes using a Sigma melting point apparatus, Sl. No. 71281, watts-250, volts-230 AC and are uncorrected. The ^{1}H, ^{13}C, DEPT, H,H-COSY, C,H-COSY and HMBC spectra were recorded on a Bruker 300 MHz NMR instrument using TMS as internal standard and $CDCl_3$ and/or DMSO-d_6 as solvents. Standard Bruker software was used throughout the spectral analysis. Chemical shifts are given in parts per million (δ-scale) and the coupling constants are given in Hertz. Elemental analyses were performed on a Perkin Elmer 2400 Series II Elemental CHNS analyzer. Silica gel-G plates (Merck) were used for TLC analysis with a mixture of petroleum ether (60–80 °C) and ethyl acetate as the eluent. All the chemicals were purchased from Sigma-Aldrich, Alfa-Aesar or Merck and used without further purification. The single crystal X-ray data set was collected on Bruker AXS KAPPA APEX-2 diffractometer equipped with graphite monochromator. The structure was solved by direct methods and refined by full-matrix least squares calculations using SHELXL-2014.

General procedure for the synthesis of indole–cycloalkyl[*b*] pyridine-3-carbonitriles 7, 12 and 14–18

A mixture of 3-(1*H*-indol-3-yl)-3-oxopropanenitrile **3** (0.1 g, 0.543 mmol), aromatic aldehyde **4** (0.543 mmol), cycloalkanone **5** (0.543 mmol) and ammonium acetate (**6**, 0.1 g, 1.1 mmol) was dissolved in ethanol (10 mL) and heated to reflux on a heating mantle for 2 h. After completion of the reaction as evident from TLC, the reaction mixture was set aside at ambient temperature for 6–7 h. The precipitate formed was filtered and dried to get the pure indole–cycloalkyl[*b*]pyridine-3-carbonitriles. The unaromatized product was obtained in the cases where the product was precipitated within 2 h.

Compounds characterization data

2-(1*H*-Indol-3-yl)-4-phenyl-5,6,7,8,9,10,11,12,13,14-decahydrocyclododeca[*b*]pyridine-3-carbonitrile (7a)

Obtained as pale yellow solid (211.9 mg, 0.488 mmol, 90%). ^{1}H NMR (300 MHz, $CDCl_3$) δ_H: 1.18–1.75 (m, 14H), 2.06–2.20 (m, 2H), 2.59 (t, *J*=7.5 Hz, 2H), 3.02 (t, *J*=7.5 Hz, 2H), 7.25–7.38 (m, 4H), 7.40–7.47 (m, 1H), 7.48–7.54 (m, 3H), 8.18 (d, *J*=3.0 Hz, 1H), 8.51–8.54 (m, 1H), 8.57 (br s, 1H) ppm; ^{13}C NMR (75 MHz, $CDCl_3$) δ_C: 22.8, 23.3, 25.9, 26.5, 26.8, 26.9, 27.3, 27.7, 28.6, 33.1, 103.5, 111.3, 114.1, 121.2, 122.2, 122.9, 126.4, 126.8, 128.3, 128.5, 128.6, 130.7, 136.2, 137.2, 153.7, 155.0, 165.0 ppm; Anal. Calcd. for $C_{30}H_{31}N_3$: C, 83.10; H, 7.21; N, 9.69; found: C 83.19, H 7.02, N 9.52.

2-(1*H*-Indol-3-yl)-4-(*p*-tolyl)-5,6,7,8,9,10,11,12,13,14-decahydrocyclododeca[*b*]pyridine-3-carbonitrile (7b)

Obtained as white solid (226.0 mg, 0.505 mmol, 93%). ^{1}H NMR (300 MHz, $CDCl_3$) δ_H: 1.19–1.88 (m, 14H), 2.01–2.25 (m, 2H), 2.45 (s, 3H), 2.61 (t, *J*=7.8 Hz, 2H), 3.03 (t, *J*=7.8 Hz, 2H), 7.18–7.36 (m, 7H), 8.15 (s, 1H), 8.47–8.56 (m, 1H), 8.72–8.78 (m, 1H) ppm; ^{13}C NMR (75 MHz, $CDCl_3$) δ_C: 18.3, 21.4, 22.8, 23.3, 25.9, 26.5, 26.9, 27.3, 27.6, 28.6, 33.0, 28.3, 103.6, 111.4, 121.0, 122.1, 122.7, 126.4, 126.8, 128.2, 129.2, 130.8, 134.2, 136.2, 138.3, 153.8, 155.2, 164.8 ppm; Anal. Calcd. for $C_{31}H_{33}N_3$: C, 83.18; H, 7.43; N, 9.39; found: C 83.28, H 7.58, N 9.26.

2-(1*H*-Indol-3-yl)-4-(4-methoxyphenyl)-5,6,7,8,9,10,11,12,13,14-decahydrocyclododeca[*b*]-pyridine-3-carbonitrile (7c)

Obtained as pale yellow solid (213.5 mg, 0.461 mmol, 85%). ^{1}H NMR (300 MHz, $CDCl_3$) δ_H: 1.22–1.76 (m, 14H), 2.04–2.22 (m, 2H), 2.62 (t, *J*=7.5 Hz, 2H), 3.02 (t, *J*=7.5 Hz, 2H), 3.88 (s, 3H), 7.04 (d, *J*=8.7 Hz, 2H), 7.24–7.30 (m, 4H), 7.35–7.39 (m, 1H), 8.16 (d, *J*=2.7 Hz, 1H), 8.49–8.55 (m, 1H), 8.67 (br s, 1H) ppm; ^{13}C NMR (75 MHz, $CDCl_3$) δ_C: 22.3, 23.3, 25.9, 26.5, 26.9, 27.3, 27.7, 28.5, 33.1, 55.2, 103.9, 111.3, 114.0, 114.1, 118.9, 121.1, 122.2, 122.9, 126.4, 126.7, 129.4, 129.6, 131.1, 136.2, 153.7, 154.9, 159.6, 164.9 ppm; Anal. Calcd. for $C_{31}H_{33}N_3O$: C, 80.31; H, 7.17; N, 9.06; found: C, 81.18; H, 7.05; N, 9.16.

2-(1*H*-Indol-3-yl)-4-(4-isopropylphenyl)-5,6,7,8,9,10,11,12,13,14-decahydrocyclododeca[*b*]-pyridine-3-carbonitrile (7d)

Obtained as white solid (234.9 mg, 0.494 mmol, 91%). ^{1}H NMR (300 MHz, $CDCl_3$) δ_H: 1.35 (d, *J*=6.9Hz, 6H), 1.39–1.72 (m, 14H), 2.08–2.22 (m, 2H), 2.63 (t, *J*=7.2Hz, 2H),

2.96–3.11 (m, 3H), 7.21–7.36 (m, 7H), 8.12 (d, *J*=3.0 Hz, 1H), 8.47–8.50 (m, 1H), 8.70 (br s, 1H) ppm; ^{13}C NMR (75 MHz, $CDCl_3$) δ_C: 23.0, 23.5, 23.9, 25.9, 26.6, 27.0, 27.3, 27.7, 28.7, 33.1, 33.9, 103.7, 111.3, 114.2, 121.1, 122.3, 122.8, 126.5, 126.6, 126.8, 128.3, 130.9, 134.6, 136.3, 149.2, 153.8, 155.3, 164.8 ppm; Anal. Calcd. for $C_{33}H_{37}N_3$: C, 83.33; H, 7.84; N, 8.83; found: C 83.20, H 7.76, N 8.92.

4-(4-Fluorophenyl)-2-(1*H*-indol-3-yl)-5,6,7,8,9,10,11,12,13,14-decahydrocyclododeca[*b*]-pyridine-3-carbonitrile (7e)

Obtained as pale yellow solid (225.6 mg, 0.499 mmol, 92%). ^{1}H NMR (300 MHz, $CDCl_3$) δ_H: 1.19–1.79 (m, 14H), 2.03–2.21 (m, 2H), 2.52–2.68 (m, 2H), 2.95–3.11 (m, 2H), 7.18–7.41 (m, 7H), 8.14–8.22 (m, 1H), 8.47–8.55 (m, 1H), 8.59 (br s, 1H) ppm; ^{13}C NMR (75 MHz, $CDCl_3$) δ_C: 22.8, 23.3, 25.9, 26.5, 26.9, 27.3, 27.7, 28.6, 33.1, 103.6, 111.4, 115.6, 115.9, 121.2, 122.2, 122.9, 126.4, 126.8, 130.2, 136.2, 153.8, 154.0, 165.2 ppm; Anal. Calcd. for $C_{30}H_{30}FN_3$: C, 79.79; H, 6.70; N, 9.31; found: C 71.62, H 6.81, N 9.28.

4-(4-Chlorophenyl)-2-(1*H*-indol-3-yl)-5,6,7,8,9,10,11,12,13,14-decahydrocyclododeca[*b*]-pyridine-3-carbonitrile (7f)

Obtained as pale yellow solid (236.3 mg, 0.505 mmol, 93%). ^{1}H NMR (300 MHz, $CDCl_3$) δ_H: 1.27–1.58 (m, 14H), 2.03–2.18 (m, 2H), 2.56 (t, *J*=7.5 Hz, 2H),), 3.01 (t, *J*=7.8 Hz, 2H), 7.26 (d, *J*=8.1 Hz, 4H), 7.33–7.41 (m, 1H), 7.49 (d, *J*=8.4 Hz, 2H), 8.16 (d, *J*=3.0 Hz, 1H), 8.45–8.53 (m, 1H), 8.58 (br s, 1H) ppm; ^{13}C NMR (75 MHz, $CDCl_3$) δ_C: 22.8, 23.3, 25.9, 26.5, 26.9, 27.3, 27.7, 28.6, 33.1, 103.2, 111.4, 113.9, 118.5, 121.2, 122.2, 122.9, 126.4, 126.8, 128.9, 129.8, 130.6, 134.8, 135.6, 136.2, 153.7, 153.9, 165.2

ppm; Anal. Calcd. for $C_{30}H_{30}ClN_3$: C, 76.99; H, 6.46; N, 8.98; found: C 76.87, H 6.59, N 8.91.

4-(4-Cyanophenyl)-2-(1*H*-indol-3-yl)-5,6,7,8,9,10,11,12,13,14-decahydrocyclododeca[*b*]-pyridine-3-carbonitrile (7h)

Obtained as white solid (212.0 mg, 0.462 mmol, 85%). ^{1}H-NMR (300 MHz, $CDCl_3$) δ_H: 1.15–1.95 (m, 14H), 2.01–2.28 (m, 2H), 2.45–2.66 (m, 2H), 3.03 (t, *J*=7.5 Hz, 2H), 7.26–7.31 (m, 2H), 7.42–7.48 (m, 3H), 7.82 (d, *J*=8.1 Hz, 2H), 8.21 (d, *J*=3.0 Hz, 1H), 8.51–8.54 (m, 1H), 8.58(br s, 1H) ppm; ^{13}C NMR (75 MHz, $CDCl_3$+DMSO-d_6) δ_C: 21.9, 22.4, 24.9, 25.5, 25.9, 26.0, 26.3, 26.7, 27.7, 32.1, 101.1, 111.0, 111.7, 112.1, 117.5, 120.0, 121.3, 121.6, 125.5, 126.8, 128.7, 131.6, 135.8, 141.2, 151.9, 153.3, 164.4 ppm; Anal. Calcd. for $C_{31}H_{30}N_4$: C, 81.19; H, 6.59; N, 12.22; found: C 81.25, H 6.44, N 12.08.

2-(1*H*-Indol-3-yl)-4-(4-nitrophenyl)-5,6,7,8,9,10,11,12,13,14-decahydrocyclododeca[*b*]-pyridine-3-carbonitrile (7i)

Obtained as pale yellow solid (246.9 mg, 0.515 mmol, 95%). ^{1}H NMR (300 MHz, $CDCl_3$) δ_H: 1.17–1.90 (m, 14H), 2.01–2.22 (m, 2H), 2.56 (t, *J*=7.5 Hz, 2H), 3.04 (t, *J*=7.5 Hz, 2H), 7.30 (q, *J*= 3Hz, 2H), 7.39–7.47 (m, 1H), 7.54 (d, *J*=8.4 Hz, 2H), 8.23 (d, *J*=3.0 Hz, 1H), 8.40 (d, *J*=8.7 Hz, 2H), 8.53–8.63 (m, 2H) ppm; ^{13}C NMR (75 MHz, $CDCl_3$) δ_C: 22.9, 23.4, 25.9, 26.4, 26.9, 27.1, 27.3, 27.7, 28.7, 33.2, 102.4, 111.4, 121.5, 122.4, 123.2, 123.9, 126.4, 126.9, 129.8, 129.9, 136.3, 143.9, 148.1, 152.6, 154.0, 165.6 ppm; Anal. Calcd. for $C_{30}H_{30}N_4O_2$: C, 75.29; H, 6.32; N, 11.71; found: C 75.19, H 6.23, N 11.59.

2-(1*H*-Indol-3-yl)-4-(*o*-tolyl)-5,6,7,8,9,10,11,12,13,14-decahydrocyclododeca[*b*]pyridine-3-carbonitrile (7j)

Obtained as pale yellow solid (228.2 mg, 0.510 mmol, 94%). ^{1}H NMR (300 MHz, $CDCl_3$) δ_H: 1.18–1.82 (m, 16H), 2.15 (s, 3H), 2.33–2.43 (m, 1H), 2.57–2.71 (m, 1H), 2.92–3.02 (m, 1H), 3.07–3.16 (m, 1H), 7.19 (d, *J*=7.2 Hz, 1H), 7.25–7.30 (m, 3H), 7.34–7.40 (m, 3H), 8.18 (d, *J*=2.7 Hz, 1H), 8.57–8.60 (m, 1H), 8.68 (br s, 1H) ppm; ^{13}C NMR (75 MHz, $CDCl_3$) δ_C: 19.8, 22.7, 23.3, 25.9, 26.6, 26.9, 27.0, 27.4, 27.7, 28.2, 33.1, 103.3, 111.3, 114.1, 118.4, 121.2, 122.3, 122.9, 126.0, 126.4, 126.8, 128.5, 128.9, 130.4, 130.6, 135.1, 136.2, 136.6, 153.9, 154.8, 165.2 ppm; Anal. Calcd. for $C_{31}H_{33}N_3$: C, 83.18; H, 7.43; N, 9.39; found: C, 83.31; H, 7.53; N, 9.48.

4-(2-Bromophenyl)-2-(1*H*-indol-3-yl)-5,6,7,8,9,10,11,12,13,14-decahydrocyclododeca[*b*]-pyridine-3-carbonitrile (7l)

Obtained as pale yellow solid (255.7 mg, 0.499 mmol, 92%). ^{1}H NMR (300 MHz, $CDCl_3$) δ_H: 1.15–1.85 (m, 14H), 1.98–2.26 (m, 2H), 2.37–2.46 (m, 1H), 2.61–2.71 (m, 1H), 2.91–3.01 (m, 1H), 3.06–3.16 (m,1H), 7.26–7.49 (m, 7H), 7.76 (d, *J*=6.9 Hz, 1H), 8.23 (d, *J*=2.7 Hz, 1H), 8.55 (br s, 1H) ppm; ^{13}C NMR (75 MHz, $CDCl_3$) δ_C: 22.7, 23.3, 25.8, 26.7, 26.9, 27.2, 27.4, 27.7, 28.2, 33.1, 103.3, 111.3, 121.3, 122.4, 122.7, 123.0, 126.4, 126.9, 127.6, 130.3, 130.4, 130.6, 133.1, 136.3, 153.7, 153.9, 165.5 ppm; Anal. Calcd. for $C_{30}H_{30}BrN_3$: C, 70.31; H, 5.90; N, 8.20; found: C 70.46, H 5.83, N 8.15.

2-(1*H*-Indol-3-yl)-4-(3-nitrophenyl)-5,6,7,8,9,10,11,12,13,14-decahydrocyclododeca[*b*]-pyridine-3-carbonitrile (7m)

Obtained as pale yellow solid (244.0 mg, 0.510 mmol, 94%). ^{1}H NMR (300 MHz, $CDCl_3$) δ_H: 1.14–1.92 (m, 14H), 2.01–2.27 (m, 2H), 2.57 (t, *J*=6.9 Hz, 2H), 2.92–3.18 (m,

2H), 7.25–7.38 (m, 3H), 7.68–7.73 (m, 2H), 8.17 (d, *J*=2.7 Hz, 1H), 8.23–8.28 (m, 1H), 8.34–8.41 (m, 1H), 8.51–8.55 (m, 1H), 8.66 (br s, 1H) ppm; ^{13}C NMR (75 MHz, $CDCl_3$) δ_C: 22.8, 23.3, 25.8, 26.4, 26.8, 26.9, 27.3, 27.7, 28.6, 33.1, 102.8, 111.4, 121.4, 122.3, 123.1, 123.6, 123.8, 126.9, 129.9, 130.3, 134.7, 136.2, 148.2, 152.2, 154.1, 165.7 ppm; Anal. Calcd. for $C_{30}H_{30}N_4O_2$: C, 75.29; H, 6.32; N, 11.71; found: C 75.40, H 6.39, N 7.83.

4-(2,4-Dichlorophenyl)-2-(1*H*-indol-3-yl)-5,6,7,8,9,10,11,12,13,14-decahydrocyclododeca[*b*]-pyridine-3-carbonitrile (7n)

Obtained as white solid (253.7 mg, 0.505 mmol, 93%). ^{1}H NMR (300 MHz, $CDCl_3$) δ_H: 1.18–1.88 (m, 12H), 1.98–2.27 (m, 3H), 2.37–2.47 (m, 1H), 2.60–2.69 (m, 1H), 2.93–3.15 (m, 3H), 7.23–7.30 (m, 2H), 7.40–7.45 (m, 3H), 7.58 (d, *J*=2.1 Hz, 1H), 8.23 (d, *J*=2.7 Hz, 1H), 8.53–8.56 (m, 2H) ppm. ^{13}C NMR (75 MHz, $CDCl_3$) δ_C: 22.9, 23.4, 25.9, 26.6, 27.3, 27.4, 27.7, 28.3, 33.3, 102.1, 111.3, 121.4, 122.5, 123.2, 126.9, 127.6, 130.0, 130.8, 131.2, 133.8, 135.7, 136.3, 151.1, 154.1, 165.6 ppm.; Anal. Calcd. For $C_{30}H_{29}Cl_2N_3$: C, 71.71; H, 5.82; N, 8.36; found: C 71.64, H 5.71, N 8.20.

4-(3,4-Dimethoxyphenyl)-2-(1*H*-indol-3-yl)-5,6,7,8,9,10,11,12,13,14-decahydrocyclododeca-[*b*]pyridine-3-carbonitrile (7s)

Obtained as pale yellow solid (243.8 mg, 0.494 mmol, 91%). ^{1}H NMR (300 MHz, $CDCl_3$+DMSO–d_6) δ_H: 1.06–1.67 (m, 16H), 1.93–2.14 (m, 2H), 2.43–2.68 (m, 2H), 3.83 (s, 3H), 3.87 (s, 3H), 6.77–6.83 (m, 2H), 6.94 (d, *J*=9.0 Hz, 1H), 7.09–7.15 (m, 2H), 7.38 (t, *J*=3.0 Hz, 1H), 8.15 (d, *J*=2.7 Hz, 1H), 8.42 (t, *J*=3.6 Hz, 1H), 10.8 (s, 1H) ppm; ^{13}C NMR (75 MHz, $CDCl_3$+DMSO–d_6) δ_C: 22.1, 22.6, 25.2, 25.8, 26.3, 26.6, 27.0, 28.1, 32.4, 55.2, 55.3, 102.6, 110.4, 111.0, 112.7, 118.3, 120.0, 120.4, 121.6, 121.7, 125.9, 126.7,

129.1, 129.9, 136.0, 148.0, 148.3, 153.3, 154.0, 164.1 ppm; Anal. Calcd. for $C_{32}H_{35}N_3O_2$: C, 77.86; H, 7.15; N, 8.51; found: C, 77.97; H, 7.25; N, 8.39.

2-(1*H*-Indol-3-yl)-4-(3,4,5-trimethoxyphenyl)-5,6,7,8,9,10,11,12,13,14-decahydrocyclododeca[*b*]pyridine-3-carbonitrile (7t)

Obtained as white solid (261.6 mg, 0.499 mmol, 92%). ^{1}H NMR (300 MHz, $CDCl_3$) δ_H: 1.27–1.87 (m, 12H), 2.01–2.28 (m, 2H), 2.53–2.76 (m, 2H), 2.93–3.16 (m, 2H), 3.77 (s, 2H), 3.88 (s, 6H), 3.95 (s, 3H), 6.51 (s, 2H), 7.26–7.44 (m, 3H), 8.21 (s, 1H), 8.46–8.56 (m, 1H), 8.68 (s, 1H) ppm; ^{13}C NMR (75 MHz, $CDCl_3$) δ_C: 22.8, 23.3, 25.9, 26.6, 27.1, 27.2, 27.4, 27.7, 29.1, 33.1, 56.1, 25.2, 61.0, 103.5, 105.8, 106.2, 111.4, 113.9, 118.6, 121.2, 122.1, 122.9, 126.4, 126.8, 130.8, 132.6, 136.3, 137.9, 153.2, 153.8, 154.8, 165.1 ppm. Anal. Calcd. For $C_{33}H_{37}N_3O_3$: C, 75.69; H, 7.12; N, 8.02; found: C, 75.74; H, 7.02; N, 8.14.

2-(1*H*-Indol-3-yl)-4-(thiophen-2-yl)-5,6,7,8,9,10,11,12,13,14-decahydrocyclododeca[*b*]-pyridine-3-carbonitrile (7u)

Obtained as brown solid; Yield (214.8 mg, 0.489 mmol, 90%). ^{1}H NMR (300 MHz, $CDCl_3$) δ_H: 1.24–1.82 (m, 14H), 2.01–2.24 (m, 2H), 2.62–2.81 (m, 2H), 2.91–3.15 (m, 2H), 7.15–7.55 (m, 6H), 8.20 (s, 1H), 8.42–8.67 (m, 2H), ppm; ^{13}C NMR (75 MHz, $CDCl_3$) δ_C:23.0, 23.5, 25.9, 26.7, 27.1, 27.3, 27.5, 27.7, 29.3, 33.2, 104.8, 111.3, 114.2, 118.4, 121.3, 122.4, 123.1, 126.5, 126.9, 127.2, 127.3, 128.9, 132.5, 136.3, 136.5, 147.8, 153.9, 165.1 ppm; Anal. Calcd. for $C_{28}H_{29}N_3S$: C, 76.50; H, 6.65; N, 9.56; found: C 76.64, H 6.57, N 9.53.

2-(5-Bromo-1*H*-indol-3-yl)-4-(*p*-tolyl)-5,6,7,8,9,10,11,12,13,14-decahydrocyclododeca[*b*]-pyridine-3-carbonitrile (14b)

Obtained as white solid (184.0 mg, 0.349 mmol, 92%). ^{1}H NMR (300 MHz, $CDCl_3$+DMSO–d_6) δ_H: 1.06–1.61 (m, 14H), 1.91–2.06 (m, 2H), 2.30 (s, 3H), 2.39–2.51 (m, 2H), 2.80–2.95 (m, 2H), 7.05 (d, *J*=8.1 Hz, 2H), 7.15–7.22 (m, 4H), 8.09 (d, *J*=3.0 Hz, 1H), 8.56 (s, 1H), 10.71 (s, 1H) ppm; ^{13}C NMR (75 MHz,, $CDCl_3$+DMSO–d_6) δ_C: 19.9, 21.5, 21.9, 24.3, 24.8, 25.3, 25.6, 26.1, 27.0, 31.5, 101.5, 111.5, 112.1, 112.2, 123.4, 123.5, 126.8, 127.1, 127.8, 129.3, 132.8, 133.9, 136.7, 151.7, 153.5, 163.1 ppm; Anal. Calcd. for $C_{31}H_{32}BrN_3$: C, 70.72; H, 6.13; N, 7.98; found: C 70.63, H 6.29, N 7.85.

2-(5-Bromo-1*H*-indol-3-yl)-4-(4-isopropylphenyl)-5,6,7,8,9,10,11,12,13,14-decahydrocyclo-dodeca[*b*]pyridine-3-carbonitrile (4d)

Obtained as white solid (189.7 mg, 0.342 mmol, 90%). ^{1}H NMR (300 MHz, $CDCl_3$) δ_H: 1.33 (d, *J*=6.9Hz, 6H), 1.37–1.76 (m, 14H), 2.06–2.22 (m, 2H), 2.54–2.67 (m, 2H), 2.92–3.11 (m, 3H), 7.23 (d, *J*=8.1 Hz, 3H), 7.36 (d, *J*=8.1 Hz, 3H), 8.17 (s, 1H), 8.65 (br s, 1H), 8.70 (s, 1H) ppm; ^{13}C NMR (75 MHz, $CDCl_3$ +DMSO–d_6) δ_C: 22.1, 22.6, 23.0, 24.9, 25.5, 25.9, 26.2, 26.6, 27.7, 32.1, 33.9, 102.2, 112.2, 112.4, 112.9, 117.7, 124.1, 125.6, 127.4, 127.5, 130.0, 133.6, 134.4, 148.1, 152.4, 154.2, 163.8 ppm; Anal. Calcd. for $C_{33}H_{36}BrN_3$: C, 71.47; H, 6.54; N, 7.58; found: C 71.55, H 5.47, N 7.65.

2-(5-Bromo-1*H*-indol-3-yl)-4-(4-chlorophenyl)-5,6,7,8,9,10,11,12,13,14-decahydrocyclo-dodeca[*b*]pyridine-3-carbonitrile (14f)

Obtained as white solid (197.4 mg, 0.361 mmol, 95%). ^{1}H NMR (300 MHz, $CDCl_3$+DMSO–d_6) δ_H: 1.10–1.73 (m, 16H), 1.91–2.14 (m, 2H), 2.82–3.05 (m, 2H), 7.22–7.38 (m, 4H), 7.48 (d, *J*=8.1 Hz, 2H), 8.19 (s, 1H), 8.61 (s, 1H), 11.63 (s, 1H) ppm; ^{13}C

NMR (75 MHz, $CDCl_3$+DMSO–d_6) δ_C: 21.2, 21.6, 23.9, 24.4, 24.9, 25.2, 25.7, 26.6, 31.2, 100.8, 110.9, 111.9, 116.5, 123.2, 126.4, 126.9, 127.1, 128.5, 128.6, 132.5, 133.6, 134.1, 151.5, 151.7, 163.0 ppm; Anal. Calcd. for $C_{30}H_{29}BrClN_3$: C, 65.88; H, 5.34; N, 7.68; found: C 65.80, H 5.22, N 7.77.

2-(5-Bromo-1*H*-indol-3-yl)-4-(4-bromophenyl)-5,6,7,8,9,10,11,12,13,14-decahydrocyclo-dodeca[*b*]pyridine-3-carbonitrile (14g)

Obtained as pale yellow solid (206.7 mg, 0.349 mmol, 92%). ^{1}H NMR (300 MHz, $CDCl_3$+DMSO–d_6) δ_H: 1.08–1.73 (m, 16H), 1.91–2.15 (m, 2H), 2.84–3.04 (m, 2H), 7.29 (d, *J*=8.1 Hz, 3H), 7.42 (d, *J*=8.7 Hz, 1H), 7.68 (d, *J*=8.1 Hz, 2H), 8.23 (s, 1H), 8.61 (s, 1H), 11.71 (s, 1H) ppm; ^{13}C NMR (75 MHz, $CDCl_3$+DMSO–d_6) δ_C: 2.08, 21.2, 23.5, 24.0, 24.5, 24.9, 25.4, 26.2, 30.7, 100.5, 110.5, 111.5, 111.9, 120.5, 122.7, 122.9, 126.1, 126.9, 128.3, 128.6, 129.7, 133.2, 134.2, 151.1, 151.5, 162.6 ppm; Anal. Calcd. for $C_{30}H_{29}Br_2N_3$: C, 60.93; H, 4.94; N, 7.11; found: C 60.81, H 5.02, N 7.25.

2-(5-Bromo-1*H*-indol-3-yl)-4-(3-nitrophenyl)-5,6,7,8,9,10,11,12,13,14-decahydrocyclodo-deca[*b*]pyridine-3-carbonitrile (14m)

Obtained as pale yellow solid (201.2 mg, 0.361 mmol, 95%). ^{1}H NMR (300 MHz, $CDCl_3$+DMSO–d_6) δ_H: 0.97–1.69 (m, 14H), 2.09–2.30 (m, 2H), 2.31–2.43 (m, 2H), 2.44–2.59 (m, 2H), 7.18 (d, *J*=8.7 Hz, 1H), 7.31 (d, *J*=8.7 Hz, 1H), 7.45–7.57 (m, 2H), 7.60–7.72 (m, 1H), 7.97–8.10 (m, 2H), 8.27 (s, 1H), 11.48 (s, 1H) ppm; ^{1}C NMR (75 MHz, $CDCl_3$+DMSO–d_6) δ_C: 22.7, 22.9, 23.1, 24.7, 25.0, 25.1, 25.4, 25.6, 26.5, 26.7, 108.9, 110.2, 113.5, 114.3, 122.5, 123.2, 125.2, 128.9, 130.2, 134.3, 145.1, 148.9, 149.1 ppm; Anal. Calcd. for $C_{30}H_{29}BrN_4O_2$: C, 64.63; H, 5.24; N, 10.05; found: C 64.56, H 5.39, N 10.14.

2-(5-Bromo-1*H*-indol-3-yl)-4-(4-chloro-2-fluorophenyl)-5,6,7,8,9, 10,11,12,13,14-decahydro-cyclododeca[*b*]pyridine-3-carbonitrile (14p)

Obtained as pale yellow solid (191.0 mg, 0.338 mmol, 89%). ^{1}H NMR (300 MHz, $CDCl_3$+DMSO–d_6) δ_H: 0.58–1.35 (m, 16H), 1.44–1.77 (m, 2H), 1.88–2.21 (br s, 2H), 6.77–6.85 (m, 5H), 7.72 (s, 1H), 8.18 (s, 1H), 10.78 (s, 1H) ppm; ^{13}C NMR (75 MHz, $CDCl_3$+DMSO–d_6) δ_C: 22.3, 22.7, 25.0, 25.5, 25.9, 26.4, 26.8, 27.5, 32.4, 102.2, 112.1, 112.6, 113.4, 116.1, 124.4, 124.5, 127.5, 127.9, 130.4, 130.8, 134.7, 147.0, 152.9, 156.5, 159.8, 164.6 ppm; Anal. Calcd. for $C_{30}H_{28}BrClFN_3$: C, 63.78; H, 5.00; N, 7.44; found: C 63.91, H 5.09, N 7.54.

2-(5-Bromo-1*H*-indol-3-yl)-4-(3,4-dimethoxyphenyl)-5,6,7,8,9,10,11,12,13,14-decahydro-cyclododeca[*b*] pyridine-3-carbonitrile (14s)

Obtained as pale yellow solid (195.8 mg, 0.342 mmol, 90%). ^{1}H NMR (300 MHz, $CDCl_3$+DMSO–d_6) δ_H: 1.08–1.72 (m, 16H), 1.90–2.14 (m, 2H), 2.80–3.03 (m, 2H), 3.79 (s, 3H), 3.85 (s, 3H), 6.73–6.92 (m, 2H), 6.94–7.08 (m, 1H), 7.18–7.30 (m, 1H), 7.33–7.44 (m, 1H), 8.21 (d, *J*=3.0 Hz, 1H), 8.62 (s, 1H), 11.64 (s, 1H) ppm. ^{13}C NMR (75 MHz, $CDCl_3$+DMSO–d_6) δ_C: 21.4, 21.8, 24.2, 24.7, 25.3, 25.5, 26.0, 27.0, 31.4, 54.3, 54.5, 101.7, 110.1, 110.8, 112.0, 112.1, 119.6, 123.3, 123.4, 126.7, 127.0, 129.3, 133.7, 147.3, 147.6, 151.6, 153.1, 162.9 Anal. Calcd. for $C_{32}H_{34}BrN_3O_2$: C, 67.13; H, 5.99; N, 7.34; found: C 67.21, H 5.91, N 7.39.

2-(5-Bromo-1*H*-indol-3-yl)-4-(3,4,5-trimethoxyphenyl)-5,6,7,8,9,10,11,12,13,14-decahydro-cyclododeca[*b*] pyridine-3-carbonitrile (14t)

Obtained as white solid (215.2 mg, 0.357 mmol, 94%). ^{1}H NMR (300 MHz, $CDCl_3$+DMSO–d_6) δ_H: 1.13–1.75 (m, 14H), 1.91–2.16 (m, 2H), 2.53–2.68 (m, 2H), 2.86–

3.02 (m, 2H), 3.73–3.85 (m, 9H), 6.61 (s, 2H), 7.21–7.29 (m, 1H), 7.37–7.44 (m, Hz, 1H), 8.25 (s, 1H), 8.62 (s, 1H), 11.71 (s, 1H) ppm; ^{13}C NMR (75 MHz, $CDCl_3$+DMSO–d_6) δ_C: 21.0, 21.4, 23.8, 24.3, 24.9, 25.0, 25.1, 25.5, 26.8, 30.9, 54.4, 58.5, 101.0, 104.4, 110.8, 111.6, 116.4, 122.9, 126.3, 126.8, 128.7, 130.5, 133.3, 136.0, 151.1, 151.2, 152.8, 162.5 ppm; Anal. Calcd. for $C_{33}H_{36}BrN_3O_3$: C, 65.78; H, 6.02; N, 6.97; found: C 65.90, H 6.09, N 7.05.

4-(4-Bromophenyl)-2-(1*H*-indol-3-yl)-1,4,5,6,7,8,9,10,11,12,13,14-dodecahydrocyclododeca-[*b*]pyridine-3-carbonitrile (12g)

Obtained as white solid (248.5 mg, 0.483 mmol, 89%). ^{1}H NMR (300 MHz, $CDCl_3$+DMSO–d_6) δ_H: 1.05–1.75 (m, 16H), 1.99–2.30 (m, 2H), 2.49–2.61 (m, 2H), 4.18 (s, 1H), 7.05–7.14 (m, 2H), 7.22 (d, *J*=9.0 Hz, 2H), 7.33–7.44 (m, 3H), 7.46–7.52 (m, 1H), 7.54–7.62 (m, 2H), 11.09 (s, 1H) ppm; ^{13}C NMR (75 MHz, $CDCl_3$+DMSO–d_6) δ_C: 21.1, 21.5, 23.0, 23.3, 23.8, 24.0, 24.9, 25.0, 43.0, 75.4, 107.9, 109.2, 111.1, 118.9, 119.1, 119.2, 121.1, 122.3, 124.3, 125.9, 128.4, 130.4, 130.7, 135.3, 143.7, 144.5 ppm; Anal. Calcd. for $C_{30}H_{32}BrN_3$: C, 70.03; H, 6.27; N, 8.17; found: C, 70.15; H, 6.37; N, 8.25.

4-(2-Chlorophenyl)-2-(1*H*-indol-3-yl)-1,4,5,6,7,8,9,10,11,12,13,14-dodecahydrocyclododeca-[*b*]pyridine-3-carbonitrile (12k)

Obtained as white solid (242.5 mg, 0.516 mmol, 95%). ^{1}H NMR (300 MHz, $CDCl_3$) δ_H: 1.21–1.89 (m, 16H), 1.95–2.13 (m, 1H), 2.15–2.34 (m, 1H), 2.54–2.72 (m, 1H), 5.04 (s, 1H), 5.89 (s, 1H), 7.10–7.22 (m, 3H), 7.23–7.33 (m, 3H), 7.34–7.44 (m, 2H), 7.50–7.57 (m, 1H), 7.60–7.65 (m, 1H), 9.22 (s, 1H) ppm; ^{13}C NMR (75 MHz, $CDCl_3$) δ_C: 22.3, 24.3, 24.4, 24.7, 24.8, 24.9, 25.6, 26.2, 26.3, 40.2, 76.7, 108.8, 111.6, 112.4, 118.9, 120.8, 122.7, 123.3, 124.7, 126.7, 127.6, 128.0, 129.3, 130.6, 130.8, 132.3, 136.1, 143.8, 145.1

ppm; Anal. Calcd. for $C_{30}H_{32}ClN_3$: C, 76.66; H, 6.86; N, 8.94; found: C, 76.80; H, 6.90; N, 8.79.

4-(2-Chloro-3-methoxyphenyl)-2-(1*H*-indol-3-yl)-1,4,5,6,7,8,9,10,11,12,13,14-dodecahydro-cyclododeca[*b*] pyridine-3-carbonitrile (12o)

Obtained as white solid (249.8 mg, 0.499 mmol, 92%). ^{1}H NMR (300 MHz, $CDCl_3$+DMSO–d_6) δ_H: 1.18–1.92 (m, 17H), 2.02–2.14 (m, 1H), 2.16–2.23 (m, 1H), 2.48–2.64 (m, 1H), 3.90 (s, 3H), 5.01 (s, 1H), 6.84 (dd, *J*=7.5 Hz, 1H), 7.10–7.25 (m, 5H), 7.42 (d, *J*=7.2 Hz, 1H), 7.56 (d, *J*=2.7 Hz, 1H), 7.62 (d, *J*=7.2 Hz, 1H), 11.0 (s, 1H) ppm; ^{13}C NMR (75 MHz, $CDCl_3$+DMSO–d_6) δ_C: 27.5, 27.7, 29.6, 29.9, 30.2, 30.3, 31.0, 31.4, 31.5, 45.7, 81.7, 114.2, 115.3, 116.2, 117.4, 125.0, 125.4, 125.7, 127.4, 127.7, 128.1, 130.5, 132.0, 132.8, 136.7, 141.6, 150.4, 151.1, 159.7 ppm; Anal. Calcd. for $C_{31}H_{34}ClN_3O$: C, 74.46; H, 6.85; N, 8.40; found: C, 74.59; H, 6.94; N, 8.49.

4-(4-Chloro-2-fluorophenyl)-2-(1*H*-indol-3-yl)-1,4,5,6,7,8,9,10,11,12,13,14-dodecahydro-cyclododeca[*b*] pyridine-3-carbonitrile (12p)

Obtained as white solid (230.3 mg, 0.472 mmol, 87%). ^{1}H NMR (300 MHz, $CDCl_3$) δ_H: 1.16–1.86 (m, 16H), 1.94–2.10 (m, 1H), 2.17–2.33 (m, 1H), 2.53–2.71 (m, 1H), 4.73 (s, 1H), 5.87 (s, 1H), 7.08–7.13 (m, 2H), 7.16–7.23 (m, 2H), 7.31–7.37 (m, 2H), 7.53 (d, *J*=2.4 Hz, 1H), 7.61–7.67 (m, 1H), 9.08 (s, 1H) ppm; ^{13}C NMR (75 MHz, $CDCl_3$) δ_C: 22.8, 22.9, 24.7, 25.0, 25.2, 25.3, 26.0, 26.6, 26.9, 37.2, 76.6, 109.5, 110.7, 112.7, 116.3, 116.7, 119.3, 121.4, 123.2, 123.3, 125.2, 125.4, 125.5, 127.0, 131.4, 131.4, 131.5, 131.6, 131.8, 133.5, 133.6, 136.6, 145.7, 158.4, 161.6 ppm; Anal. Calcd. for $C_{30}H_{31}ClFN_3$: C, 73.83; H, 6.40; N, 8.61; found: C, 73.78; H, 6.51; N, 8.70.

4-(2,5-Dimethoxyphenyl)-2-(1*H*-indol-3-yl)-1,4,5,6,7,8,9,10,11,12,13,14-dodecahydrocyclo-dodeca[*b*]pyridine-3-carbonitrile (12q)

Obtained as white solid (228.9 mg, 0.462 mmol, 85%). ^{1}H NMR (300 MHz, $CDCl_3$+DMSO–d_6) δ_H: 1.14–1.88 (m, 17H), 2.05–2.60 (m, 2H), 2.39–2.58 (m, 1H), 3.64 (s, 3H), 3.77 (s. 3H), 4.67 (s, 1H), 6.66 (dd, *J*=9.0 Hz, 1H), 6.79 (d, *J*=7.5 Hz, 1H), 6.90 (d, *J*=3.0 Hz, 1H), 7.03–7.13 (m, 2H), 7.38 (d, *J*=7.8 Hz, 1H), 7.51 (d, *J*=2.4 Hz, 1H), 7.55 (d, *J*=7.5 Hz, 1H), 7.75 (s, 1H), 11.24 (s. 1H) ppm; ^{13}C NMR (75 MHz, $CDCl_3$+DMSO–d_6) δ_C: 20.7, 21.1, 22.7, 23.4, 24.5, 24.6, 24.7, 34.4, 53.9, 55.1, 74.7, 107.8, 109.8, 110.1, 110.3, 110.6, 114.3, 118.5, 118.7, 120.6, 122.0, 124.0, 125.4, 130.1, 134.9, 135.3, 143.8, 149.0, 152.7 ppm; Anal. Calcd. for $C_{32}H_{37}N_3O_2$: C, 77.54; H, 7.52; N, 8.48; found: C, 77.45; H, 7.48; N, 8.55.

4-(2,6-Difluorophenyl)-2-(1*H*-indol-3-yl)-1,4,5,6,7,8,9,10,11,12,13,14-dodecahydrocyclodo-deca[*b*]pyridine-3-carbonitrile (12r)

Obtained as white solid (227.8 mg, 0.483 mmol, 89%). ^{1}H NMR (300 MHz, $CDCl_3$+DMSO–d_6) δ_H: 1.11–1.79 (br s, 17H), 1.88–2.05 (m, 1H), 2.09–2.26 (m, 1H), 2.33–2.46 (m, 1H), 4.87 (s, 1H), 6.83 (t, *J*=8.4 Hz, 2H), 7.02–7.18 (m, 3H), 7.35 (d, *J*=7.5 Hz, 1H), 7.50 (d, *J*=2.7 Hz, 1H), 7.56 (d, *J*=6.0 Hz, 1H), 7.61 (s, 1H), 11.09 (s, 1H) ppm; ^{13}C NMR (75 MHz, $CDCl_3$+DMSO–d_6) δ_C: 27.5, 27.7, 29.6, 29.8, 30.0, 30.2, 30.3, 30.9, 31.3, 31.4, 39.2, 78.2, 112.4, 114.3, 116.5, 116.9, 117.3, 125.2, 125.3, 125.5, 125.7, 125.9, 127.3, 128.3, 130.6, 130.8, 133.4, 133.6, 133.7, 137.3, 141.6, 151.5, 165.3, 165.4, 168.6, 168.7 ppm; Anal. Calcd. for $C_{30}H_{31}F_2N_3$: C, 76.41; H, 6.63; N, 8.91; found: C, 76.55; H, 6.77; N, 8.99.

2-(1*H*-Indol-3-yl)-4-phenyl-5,6,7,8,9,10-hexahydrocycloocta[*b*]pyridine-3-carbonitrile (15a)

Obtained as white solid (188.9 mg, 0.499 mmol, 92%). ^{1}H NMR (300 MHz, $CDCl_3$) δ_H: 1.33–1.70 (m, 6H), 1.90–2.06 (m, 2H), 2.60–2.80 (m, 2H), 3.11–3.33 (m, 2H), 7.17–7.66 (m, 8H), 8.19 (s, 1H), 8.47–8.71 (m, 2H), ppm; ^{13}C NMR (75 MHz, $CDCl_3$) δ_C: 25.7, 26.5, 27.4, 30.8, 31.0, 36.1, 103.7, 111.3, 114.0, 121.2, 122.3, 122.9, 126.5, 126.7, 128.2, 128.6, 128.7, 130.3, 136.2, 154.1, 154.2, 165.6 ppm; Anal. Calcd. for $C_{26}H_{23}N_3$: C, 82.73; H, 6.14; N, 11.13; found: C 82.65, H 6.07, N 11.19.

2-(1*H*-Indol-3-yl)-4-(*p*-tolyl)-5,6,7,8,9,10-hexahydrocycloocta[*b*]pyridine-3-carbonitrile(15b)

Obtained as white solid (197.7 mg, 0.505 mmol, 93%). ^{1}H NMR (300 MHz, $CDCl_3$) δ_H: 1.32–1.61 (m, 6H), 1.88–2.06 (m, 2H), 2.44 (s, 3H), 2.60–2.74 (m, 2H), 3.11–3.25 (m, 2H), 7.19–7.39 (m, 7H), 8.17 (d, *J*=3.0 Hz, 1H), 8.48–8.56 (m, 1H), 8.61 (br s, 1H) ppm; ^{13}C NMR (75 MHz, $CDCl_3$) δ_C: 21.4, 25.7, 26.5, 27.3, 30.7, 31.0, 36.1, 1039, 111.3, 121.1, 122.2, 122.9, 126.4, 126.7, 128.1, 129.3, 130.5, 134.0, 136.2, 138.4, 154.2, 154.3, 165.5 ppm; Anal. Calcd. for $C_{27}H_{25}N_3$: C, 82.83; H, 6.44; N, 10.73; found: C 82.94, H 6.54, N 10.81.

2-(1*H*-Indol-3-yl)-4-(4-isopropylphenyl)-5,6,7,8,9,10-hexahydrocycloocta[*b*]pyridine-3-carbonitrile (15d)

Obtained as white solid (209.6 mg, 0.499 mmol, 92%). ^{1}H NMR (300 MHz, $CDCl_3$) δ_H: 1.33 (d, *J*=6.9 Hz, 6H), 1.37–1.59 (m, 6H), 1.90–2.05 (m, 2H), 2.62–2.74 (m, 2H), 2.92–3.07 (m, 1H), 3.13–3.25 (m, 2H), 7.22–7.29 (m, 4H), 7.36 (d, *J*=7.8 Hz, 3H), 8.14–8.21 (m, 1H), 8.47–8.56 (m, 1H), 8.60 (s, 1H) ppm; ^{13}C NMR (75 MHz, $CDCl_3$) δ_C: 23.8, 25.7, 26.5, 27.3, 30.7, 31.1, 33.8, 36.0, 103.9, 111.3, 121.0, 122.1, 122.7, 126.4, 126.6, 126.7,

128.1, 130.5, 134.2, 136.2, 149.1, 154.2, 154.3, 165.5 ppm; Anal. Calcd. for $C_{29}H_{29}N_3$: C, 83.02; H, 6.97; N, 10.02; found: C, 83.19; H, 6.89; N 10.18.

4-(4-Fluorophenyl)-2-(1*H*-indol-3-yl)-5,6,7,8,9,10-hexahydrocycloocta[*b*]pyridine-3-carbonitrile (15e)

Obtained as yellow solid (201.6 mg, 0.510 mmol, 94%). ^{1}H NMR (300 MHz, $CDCl_3$) δ_H: 1.30–1.67 (m, 6H), 1.84–2.06 (m, 2H), 2.56–2.78 (m, 2H), 3.09–3.31 (m, 2H), 7.20–7.38 (m, 7H), 8.15 (d, *J*=2.7 Hz, 1H), 8.49–8.58 (m, 1H), 8.70 (s, 1H) ppm; ^{13}C NMR (75 MHz, $CDCl_3$) δ_C: 25.7, 26.5, 27.3, 30.7, 30.9, 36.0, 103.7, 111.4, 113.8, 115.6, 115.9, 121.2, 122.2, 122.9, 126.7, 130.1, 130.2, 130.4, 136.3, 153.1, 154.3, 161.2, 164.4, 165.7 ppm; Anal. Calcd. for $C_{26}H_{22}FN_3$; C, 78.96; H, 5.61; N, 10.63; found: C 78.83, H 5.70, N 10.52.

4-(4-Chlorophenyl)-2-(1*H*-indol-3-yl)-5,6,7,8,9,10-hexahydrocycloocta[*b*]pyridine-3-carbonitrile (15f)

Obtained as yellow solid (201.3 mg, 0.489 mmol, 90%). ^{1}H NMR (300 MHz, $CDCl_3$) δ_H: 1.32–1.66 (m, 6H), 1.88–2.09 (s, 2H), 2.65 (t, *J*=6.0Hz, 2H), 3.18 (t, *J*=6.0Hz, 2H), 7.18–7.43 (m, 5H), 7.49 (d, *J*=8.4 Hz, 2H), 8.15 (d, *J*=2.7 Hz, 1H), 8.47–8.60 (m, 1H), 8.66 (br s, 1H) ppm; ^{13}C NMR (75 MHz, $CDCl_3$) δ_C: 25.7, 26.5, 27.4, 30.7, 31.0, 36.1, 103.5, 111.3, 113.9, 121.3, 122.3, 123.1, 126.4, 126.8, 129.0, 129.7, 130.3, 134.9, 135.4, 136.3, 152.9, 154.3, 165.8 ppm; Anal. Calcd. for $C_{26}H_{22}ClN_3$: C, 75.81; H, 5.38; N, 10.20; found: C 75.96, H 5.46, N 10.28.

4-(4-Bromophenyl)-2-(1*H*-indol-3-yl)-5,6,7,8,9,10-hexahydrocycloocta[*b*]pyridine-3-carbonitrile (15g)

Obtained as yellow solid (225.5 mg, 0.494 mmol, 91%). ^{1}H NMR (300 MHz, $CDCl_3$) δ_H: 1.30–1.71 (m, 6H), 1.86–2.14 (m, 2H), 2.53–2.81 (m, 2H), 3.05–3.33 (m, 2H), 7.20 (d, *J*=6.0 Hz, 2H), 7.24–7.35 (m, 2H), 7.37–7.51 (m, 1H), 7.66 (d, *J*=8.1 Hz, 2H), 8.22 (s, 1H), 8.56 (s, 2H) ppm; ^{13}C NMR (75 MHz, $CDCl_3$) δ_C: 25.7, 26.5, 27.4, 30.7, 31.0, 36.1, 103.5, 111.3, 113.9, 121.3, 122.3, 123.1, 126.4, 126.8, 129.0, 129.7, 130.3, 134.9, 135.4, 136.3, 152.9, 154.3, 165.8 ppm; Anal. Calcd. for $C_{26}H_{22}BrN_3$: C, 68.43; H, 4.86; N, 9.21; found: C, 68.60; H, 4.77; N, 9.09.

4-(4-Cyanophenyl)-2-(1*H*-indol-3-yl)-5,6,7,8,9,10-hexahydrocycloocta[*b*]pyridine-3-carbonitrile (15h)

Obtained as white solid (179.1 mg, 0.445 mmol, 82%). ^{1}H NMR (300 MHz, $CDCl_3$) δ_H: 1.31–1.57 (m, 6H), 1.88–2.10 (m, 2H), 2.52–2.75 (m, 2H), 3.08–3.32 (t, 2H), 7.27–7.31 (m, 2H), 7.42–7.52 (m, 3H), 7.83 (d, *J*=7.5 Hz, 2H), 8.19–8.25 (m, 1H), 8.50–8.63 (m, 2H) ppm; ^{13}C NMR (75 MHz, $CDCl_3$) δ_C: 25.6, 26.4, 27.4, 30.7, 30.9, 36.1, 102.6, 111.4, 112.9, 118.3, 121.4, 122.4, 123.2, 126.8, 129.3, 129.6, 132.5, 136.2, 141.7, 151.9, 154.2, 166.2 ppm; Anal. Calcd. for $C_{27}H_{22}N_4$: C, 80.57; H, 5.51; N, 13.92; found: C 80.49, H 5.46, N 13.90.

2-(1*H*-indol-3-yl)-4-(4-nitrophenyl)-5,6,7,8,9,10-hexahydrocycloocta[*b*]pyridine-3-carbonitrile (15i)

Obtained as pale yellow solid (211.0 mg, 0.499 mmol, 92%). ^{1}H NMR (300 MHz, $CDCl_3$) δ_H: 1.30–1.62 (m, 6H), 1.88–2.08 (m, 2H), 2.51–2.73 (m, 2H), 3.10–3.31 (m, 2H), 7.26–7.40 (m, 2H), 7.36–7.44 (m, 1H), 7.52 (d, *J*=8.1 Hz, 2H), 8.21 (d, *J*=2.7 Hz, 1H),

8.39 (d, *J*=8.4 Hz, 2H), 8.54–8.59 (m, 2H) ppm; ^{13}C NMR (75 MHz, $CDCl_3$) δ_C: 25.6, 26.4, 27.4, 30.7, 30.9, 36.1, 102.5, 111.4, 121.4, 122.3, 123.2, 124.0, 126.3, 126.8, 129.5, 129.6, 136.2, 143.6, 148.0, 151.6, 154.4, 166.3 ppm; Anal. Calcd. for $C_{26}H_{22}N_4O_2$: C, 73.92; H, 5.25; N, 13.26; found: C 73.80, H 5.39, N 13.35.

2-(1*H*-indol-3-yl)-4-(*o*-tolyl)-1,4,5,6,7,8,9,10-octahydrocycloocta[*b*]pyridine-3-carbonitrile (15j)

Obtained as pale yellow solid (194.4 mg, 0.494 mmol, 91%). ^{1}H NMR (300 MHz, $CDCl_3$+DMSO–d_6) δ_H: 1.15–1.86 (m, 9H), 1.90–2.07 (m, 1H), 2.17–2.39 (m, 2H), 2.44 (s, 3H), 4.60 (s, 1H), 7.10–7.22 (m, 5H), 7.40–7.50 (m, 3H), 7.51 (d, *J*=2.4 Hz, 1H), 7.57–7.62 (m, 1H), 11.02 (s, 1H) ppm; ^{13}C NMR (75 MHz, $CDCl_3$+DMSO–d_6) δ_C: 18.8, 25.3, 25.7, 27.8, 28.0, 28.3, 40.8, 108.2, 109.1, 111.1, 119.1, 121.2, 122.6, 124.5, 125.4, 125.5, 125.6, 129.1, 129.2, 130.1, 133.9, 135.3, 144.0, 144.5 ppm; Anal. Calcd. for $C_{27}H_{27}N_3$: C, 82.41; H, 6.92; N, 10.68; found: C, 82.57; H, 6.82; N, 10.80.

4-(2-Bromophenyl)-2-(1*H*-indol-3-yl)-1,4,5,6,7,8,9,10-octahydrocycloocta[*b*]pyridine-3-carbonitrile (15l)

Obtained as pale yellow solid (233.8 mg, 0.510 mmol, 94%). ^{1}H NMR (300 MHz, $CDCl_3$+DMSO–d_6) δ_H: 1.12–2.45 (m, 12H), 4.89 (s, 1H), 7.02–7.15 (m, 3H), 7.30 (t, *J*=7.5 Hz, 1H), 7.38 (d, *J*=8.1 Hz, 1H), 7.44 (d, *J*=8.1 Hz, 1H), 7.53 (d, *J*=2.7 Hz, 1H), 7.58–7.62 (m, 3H), 11.02 (s, 1H) ppm; ^{13}C NMR (75 MHz, $CDCl_3$+DMSO–d_6) δ_C: 25.3, 25.6, 27.6, 27.8, 28.0, 28.3, 43.5, 75.9, 108.0, 108.9, 111.1, 119.1, 121.2, 121.3, 122.0, 124.5, 125.7, 127.3, 130.5, 130.9, 131.1, 135.3, 144.6, 145.5 ppm; Anal. Calcd. for $C_{26}H_{24}BrN_3$: C, 68.12; H, 5.28; N, 9.17; found: C, 68.26; H, 5.34; N, 9.27.

2-(1*H*-indol-3-yl)-4-(3-nitrophenyl)-5,6,7,8,9,10-hexahydrocycloocta[*b*]pyridine-3-carbonitrile (15m)

Obtained as pale yellow solid (211.0 mg, 0.499 mmol, 92%). ^{1}H NMR (300 MHz, $CDCl_3$) δ_H: 1.31–1.67 (m, 6H), 1.89–2.05 (m, 2H), 2.55–2.73 (m, 2H), 3.21 (t, *J*=6.3 Hz, 2H), 7.26–7.32 (m, 2H), 7.41–7.44 (m, 1H), 7.66–7.75 (m, 2H), 8.21–8.24 (m, 2H), 8.35–8.57 (m, 1H), 8.49–8.57 (m, 1H), 8.61 (br s, 1H) ppm; ^{13}C NMR (75 MHz, $CDCl_3$) δ_C: 25.6, 26.4, 27.4, 30.7, 30.9, 36.1, 102.9, 111.4, 121.4, 122.3, 123.1, 123.5, 123.8, 126.3, 126.9, 129.9, 134.6, 136.2, 138.5, 148.2, 151.2, 154.5, 166.3 ppm; Anal. Calcd. for $C_{26}H_{22}N_4O_2$: C, 73.92; H, 5.25; N, 13.26; found: C, 73.82; H, 5.09; N, 13.18.

4-(2,4-Dichlorophenyl)-2-(1*H*-indol-3-yl)-5,6,7,8,9,10-hexahydrocycloocta[*b*]pyridine-3-carbonitrile (15n)

Obtained as white solid (206.0 mg, 0.461 mmol, 85%). ^{1}H NMR (300 MHz, $CDCl_3$) δ_H: 1.28–1.67 (m, 6H), 1.85–2.08 (m, 2H), 2.44–2.80 (m, 2H), 3.08–3.36 (m, 2H), 7.26–7.42 (m, 5H), 7.59 (s, 1H), 8.14 (s, 1H), 8.47–8.61 (m, 1H), 8.70 (br s, 1H) ppm; ^{13}C NMR (75 MHz, $CDCl_3$) δ_C: 25.7, 26.4, 27.6, 30.2, 30.7, 36.1, 103.3, 111.4, 121.3, 122.3, 123.1, 126.8, 127.6, 130.0, 130.3, 130.9, 133.7, 135.7, 136.2, 150.1, 154.5, 166.3 ppm; Anal. Calcd. for $C_{26}H_{21}Cl_2N_3$: C, 69.96; H, 4.74; N, 9.41; found: C 69.85, H 4.66, N 9.53.

4-(4-Chloro-2-fluorophenyl)-2-(1*H*-indol-3-yl)-5,6,7,8,9,10-hexahydrocycloocta[*b*]pyridine-3-carbonitrile (15p)

Obtained as white solid (219.2 mg, 0.510 mmol, 94%). ^{1}H NMR (300 MHz, $CDCl_3$) δ_H: 1.28–1.72 (m, 6H), 1.87–2.12 (m, 2H), 2.54–2.85 (m, 2H), 3.21 (t, *J*=6.3 Hz, 2H), 7.21–7.42 (m, 6H), 8.20 (d, *J*=3.0 Hz, 1H), 8.52–8.57 (m, 1H), 8.60 (br s, 1H) ppm; ^{13}C NMR (75 MHz, $CDCl_3$) δ_C: 25.6, 26.4, 27.7, 30.5, 30.7, 36.1, 103.7, 111.4, 113.8, 118.2, 121.3,

122.2, 123.0, 126.3, 126.8, 130.9, 136.2, 146.8, 154.5, 157.2, 160.5, 166.1 ppm; Anal. Calcd. for $C_{26}H_{21}ClFN_3$: C, 72.64; H, 4.92; N, 9.77; found: C, 72.73; H, 4.80; N, 9.63.

4-(2,6-Difluorophenyl)-2-(1*H*-indol-3-yl)-1,4,5,6,7,8,9,10-octahydrocycloocta[*b*]pyridine-3-carbonitrile (15r)

Obtained as white solid (207.5 mg, 0.499 mmol, 92%). ^{1}H NMR (300 MHz, $CDCl_3$+DMSO–d_6) δ_H: 1.07–1.268(m, 1H), 1.41–1.87 (m, 7H), 2.05 (t, *J*=6.3 Hz, 2H), 2.13–2.42 (m, 2H), 4.96 (s, 1H), 5.84 (s, 1H), 6.91 (t, *J*=8.4 Hz, 2H), 7.14–7.41 (m, 4H), 7.56 (d, *J*=2.7 Hz, 1H), 7.65–7.75 (m, 1H), 9.00 (s, 1H) ppm; ^{13}C NMR (75 MHz, $CDCl_3$+DMSO–d_6) δ_C: 25.8, 27.8, 28.0, 28.3, 28.5, 43.8, 76.1, 108.3, 109.1, 111.4, 119.3, 121.4, 121.6, 122.3, 124.7, 125.9, 127.6, 130.8, 131.1, 131.3, 135.6, 144.9, 145.7 ppm; Anal. Calcd. for $C_{26}H_{23}F_2N_3$: C, 75.16; H, 5.58; N, 10.11; found: C, 75.29; H, 5.64; N, 10.04.

4-(3,4-Dimethoxyphenyl)-2-(1*H*-indol-3-yl)-5,6,7,8,9,10-hexahydrocycloocta[*b*]pyridine-3-carbonitrile (15s)

Obtained as white solid (218.5 mg, 0.499 mmol, 92%). ^{1}H NMR (300 MHz, $CDCl_3$) δ_H: 1.30–1.68 (m, 6H), 1.87–2.14 (m, 2H), 2.59–2.85 (m, 2H), 3.06–3.37 (m, 2H), 3.90 (s, 3H), 3.94 (s, 3H), 6.81 (s, 1H), 6.87 (d, *J*=8.1 Hz, 1H), 6.99 (d, *J*=8.1 Hz, 1H), 7.20–7.31 (m, 2H), 7.31–7.40 (m, 1H), 8.11–8.21 (m, 1H), 8.45–8.60 (m, 1H), 8.81 (s, 1H) ppm; ^{13}C NMR (75 MHz, $CDCl_3$) δ_C: 25.8, 26.5, 27.4, 30.7, 31.3, 36.0, 55.8, 56.0, 104.0, 111.1, 111.3, 111.5, 121.0, 121.1, 122.2, 122.9, 126.7, 129.4, 130.7, 136.2, 148.8, 149.1, 153.9, 154.2, 165.5 ppm; Anal. Calcd. for $C_{28}H_{27}N_3O_2$: C, 76.86; H, 6.22; N, 9.60; found: C, 76.98; H, 6.31; N, 9.71.

2-(1*H*-Indol-3-yl)-4-(3,4,5-trimethoxyphenyl)-5,6,7,8,9,10-hexahydrocycloocta[*b*]pyridine-3-carbonitrile (15t)

Obtained as white solid (241.1 mg, 0.515 mmol, 95%). ^{1}H NMR (300 MHz, $CDCl_3$) δ_H: 1.35–1.69 (m, 6H), 1.90–2.07 (m, 2H), 2.62–2.79 (m, 2H), 3.09–3.26 (m, 2H), 3.88 (s, 6H), 3.94 (s, 3H), 6.43–6.56 (m, 2H), 7.19–7.32 (m, 2H), 7.34–7.46 (m, 1H), 8.16–8.25 (m, 1H), 8.46–8.58 (m, 1H), 8.68 (s, 1H) ppm; ^{13}C NMR (75 MHz, $CDCl_3$) δ_C: 25.9, 26.4, 27.6, 30.7, 31.6, 35.9, 56.3, 62.0, 103.6, 105.7, 111.3, 113.9, 118.6, 121.2, 122.2, 122.9, 126.4, 126.7, 130.4, 132.4, 136.3, 138.0, 153.2, 153.9, 154.2, 165.6 ppm; Anal. Calcd. for $C_{29}H_{29}N_3O_3$: C, 74.50; H, 6.25; N, 8.99; found: C 74.59, H 6.36, N 8.85.

2-(1*H*-Indol-3-yl)-4-(thiophen-2-yl)-5,6,7,8,9,10-hexahydrocycloocta[*b*]pyridine-3-carbonitrile (15u)

Obtained as pale yellow solid (195.6 mg, 0.510 mmol, 94%). ^{1}H NMR (300 MHz, $CDCl_3$) δ_H: 1.33–1.80 (m, 6H),1.87–2.09 (m, 2H), 2.79 (t, *J*=6.0 Hz, 2H), 3.20 (t, *J*=6.0 Hz, 2H), 7.14–7.21 (m, 2H), 7.26–7.30 (m, 2H), 7.36–7.40 (m, 1H), 7.53 (dd, *J*=5.1 Hz, 1H), 8.19 (d, *J*=2.7 Hz, 1H), 8.46–8.55 (m, 1H), 8.67 (s, 1H) ppm; ^{13}C NMR (75 MHz, $CDCl_3$) δ_C: 25.8, 25.9, 26.4, 27.9, 30.7, 31.8, 35.9, 105.0, 111.3, 113.9, 118.4, 121.2, 122.3, 122.9, 126.4, 126.8, 127.2, 127.3, 128.5, 128.9, 132.2, 136.2, 136.3, 146.8, 154.4, 165.6 ppm; Anal. Calcd. for $C_{24}H_{21}N_3S$: C, 75.16; H, 5.52; N, 10.96; found: C, 75.11; H, 5.43; N 10.94.

2-(5-Bromo-1*H*-indol-3-yl)-4-phenyl-5,6,7,8,9,10-hexahydrocycloocta[*b*]pyridine-3-carbonitrile (16a)

Obtained as pale yellow solid (152.6 mg, 0.334 mmol, 88%). ^{1}H NMR (300 MHz, $CDCl_3$+DMSO–d_6) δ_H: 1.20–1.62 (m, 6H), 1.76–1.99 (m, 2H), 2.44–2.67 (m, 2H), 2.95–

3.25 (m, 2H), 7.15–7.62 (m, 7H), 8.24 (s, 1H), 8.62 (s, 1H), 11.71 (br s, 1H) ppm; ^{13}C NMR (75 MHz, $CDCl_3$+DMSO–d_6) δ_C: 23.8, 24.4, 25.3, 28.8, 28.9, 34.0, 101.1, 110.8, 111.7, 111.9, 116.5, 123.0, 123.1, 126.4, 126.8, 128.2, 133.5, 135.1, 151.7, 152.0, 163.4 ppm; Anal. Calcd. for $C_{26}H_{22}BrN_3$: C, 68.43; H, 4.86; N, 9.21; Found: C, 68.58; H, 4.75; N, 9.30.

2-(5-Bromo-1*H*-indol-3-yl)-4-(*p*-tolyl)-5,6,7,8,9,10-hexahydrocycloocta[*b*]pyridine-3-carbonitrile (16b)

Obtained as white solid (159.1 mg, 0.338 mmol, 89%). ^{1}H NMR (300 MHz, $CDCl_3$) δ_H: 1.34–1.61 (m, 6H), 1.89–2.06 (m, 2H), 2.45 (s, 3H), 2.62–2.76 (m, 2H), 3.13–3.27 (m, 2H), 7.18–7.31 (m, 6H), 8.14 (d, *J*=3.0 Hz, 1H), 8.65 (s, 1H), 8.83 (s, 1H) ppm; ^{13}C NMR (75 MHz, $CDCl_3$) δ_C: 21.4, 25.7, 26.4, 27.3, 30.7, 31.0, 36.0, 104.0, 112.8, 113.4, 114.5, 124.8, 125.7, 127.5, 128.0, 129.3, 131.0, 133.8, 134.8, 138.5, 153.6, 154.4, 165.7 ppm; Anal. Calcd. for $C_{27}H_{24}BrN_3$: C, 68.94; H, 5.14; N, 8.93; Found: C, 68.82; H, 5.26; N, 8.80.

2-(5-Bromo-1*H*-indol-3-yl)-4-(4-methoxyphenyl)-5,6,7,8,9,10-hexahydrocycloocta[*b*]-pyridine-3-carbonitrile (16c)

Obtained as white solid (164.5 mg, 0.338 mmol, 89%). ^{1}H NMR (300 MHz, $CDCl_3$) δ_H: 1.33–1.63 (m, 6H), 1.89–2.07 (m, 2H), 2.61–2.77 (m, 2H), 3.10–3.28 (m, 2H), 3.87 (s, 3H), 7.04 (d, *J*=8.7 Hz, 2H), 7.18–7.34 (m, 4H), 8.11 (d, *J*=3.0 Hz, 1H), 8.65 (s, 1H), 8.96 (br s, 1H) ppm; ^{13}C NMR (75 MHz, $CDCl_3$) δ_C: 25.7, 26.4, 27.3, 30.7, 30.9, 36.0, 55.2, 104.1, 112.8, 113.3, 114.0, 114.4, 118.7, 124.7, 125.6, 127.5, 128.0, 128.8, 129.4, 131.2, 134.8, 153.6, 154.0, 159.7, 165.7 ppm; Anal. Calcd. for $C_{27}H_{24}BrN_3O$: C, 66.67; H, 4.97; N, 8.64; found: C, 66.58; H, 4.86; N, 8.70.

2-(5-Bromo-1*H*-indol-3-yl)-4-(4-isopropylphenyl)-5,6,7,8,9,10-hexahydrocycloocta[*b*]-pyridine-3-carbonitrile (16d)

Obtained as white solid (170.5 mg, 0.342 mmol, 90%). ^{1}H NMR (300 MHz, $CDCl_3$+DMSO–d_6) δ_H: 1.27 (d, *J*=6.9, 6H), 1.30–1.51 (m, 6H), 1.79–2.02 (m, 2H), 2.50–2.69 (m, 2H), 2.87–3.00 (m, 1H), 3.03–3.21 (m, 2H), 7.18 (d, *J*=8.1 Hz, 2H), 7.25–7.40 (m, 5H), 8.20–8.27 (m, 1H), 8.66 (s, 1H) ppm; ^{13}C NMR (75 MHz, $CDCl_3$+DMSO–d_6) δ_C: 22.3, 24.0, 24.7, 25.5, 29.0, 29.3, 32.0, 34.3, 101.5, 111.2, 112.1, 116.9, 123.3, 124.9, 126.6, 126.7, 126.9, 128.7, 132.6, 133.7, 147.2, 152.0, 152.4, 163.5 ppm; Anal. Calcd. for $C_{29}H_{28}BrN_3$: C, 69.88; H, 5.66; N, 8.43; found: C, 69.95; H, 5.59; N, 8.56.

2-(5-Bromo-1*H*-indol-3-yl)-4-(4-fluorophenyl)-5,6,7,8,9,10-hexahydrocycloocta[*b*]pyridine-3-carbonitrile (16e)

Obtained as pale yellow solid (165.8 mg, 0.349 mmol, 92%). ^{1}H NMR (300 MHz, $CDCl_3$) δ_H: 1.35–1.59 (m, 6H), 1.89–2.06 (m, 2H), 2.59–2.73 (m, 2H), 3.12–3.27 (m, 2H), 7.15–7.42 (m, 6H), 8.22 (d, *J*=2.1 Hz, 1H), 8.58–8.73 (m, 2H) ppm;^{13}C NMR (75 MHz, $CDCl_3$) δ_C: 24.7, 25.5, 26.3, 29.7, 29.9, 35.1, 102.3, 111.9, 112.4, 113.0, 114.6, 114.9, 117.5, 124.1, 124.2, 127.3, 127.5, 129.3, 129.4, 132.0, 134.4, 152.0, 152.9, 160.1, 163.4, 164.7 ppm; Anal. Calcd. for $C_{26}H_{21}BrFN_3$: C, 65.83; H, 4.46; N, 8.86; found: C, 65.76; H, 4.51; N, 8.81.

2-(5-Bromo-1*H*-indol-3-yl)-4-(4-chlorophenyl)-5,6,7,8,9,10-hexahydrocycloocta[*b*]pyridine-3-carbonitrile (16f)

Obtained as white solid (169.7 mg, 0.345 mmol, 91%). ^{1}H NMR (300 MHz, $CDCl_3$) δ_H: 1.31–1.65 (m, 6H), 1.88–2.07 (m, 2H), 2.55–2.75 (m, 2H), 3.08–3.30 (m, 2H), 7.20–7.34 (m, 4H), 7.51 (d, *J*=7.5 Hz, 2H), 8.07–8.16 (m, 1H), 8.67 (s, 1H), 8.85 (br s, 1H) ppm;

^{13}C NMR (75 MHz, $CDCl_3$) δ_C: 25.7, 26.5, 27.4, 30.7, 31.0, 36.1, 103.5, 112.7, 113.6, 114.7, 125.1, 126.0, 127.5, 128.1, 129.0, 129.7, 130.7, 134.9, 135.0, 135.2, 152.9, 153.7, 166.1 ppm; Anal. Calcd. for $C_{26}H_{21}BrClN_3$: C, 63.62; H, 4.31; N, 8.56; found: C, 63.53; H, 4.21; N, 8.69.

2-(5-Bromo-1*H*-indol-3-yl)-4-(4-bromophenyl)-5,6,7,8,9,10-hexahydrocycloocta[*b*]pyridine-3-carbonitrile (16g)

Obtained as pale yellow solid (193.2 mg, 0.361 mmol, 95%). ^1H NMR (300 MHz, $CDCl_3$) δ_H: 1.28–1.63 (m, 6H), 1.86–2.10 (m, 2H), 2.55–2.81 (m, 2H), 3.08–3.32 (m, 2H), 7.18–7.35 (m, 4H), 7.66 (d, *J*=8.1 Hz, 2H), 8.17 (s, 1H), 8.67 (s, 1H), 8.76 (br s, 1H) ppm;^{13}C NMR (75 MHz, $CDCl_3$) δ_C: 25.7, 26.5, 27.4, 30.7, 31.0, 36.1, 103.4, 112.7, 114.7, 123.2, 125.0, 126.0, 127.5, 128.1, 129.9, 130.6, 132.0, 134.8, 135.7, 152.9, 153.6, 166.1 ppm; Anal. Calcd. for $C_{26}H_{21}Br_2N_3$: C, 58.34; H, 3.95; N, 7.85; found: C, 58.48; H, 3.81; N, 7.75.

2-(5-Bromo-1*H*-indol-3-yl)-4-(2-chloro-3-methoxy phenyl)-1,4,5,6,7,8,9,10-octahydrocyclo-octa[*b*]pyridine-3-carbonitrile (16o)

Obtained as pale yellow solid (178.8 mg, 0.342 mmol, 90%). ^1H NMR (300 MHz, $CDCl_3$+DMSO–d_6) δ_H: 1.07–2.15 (m, 10H), 2.22–2.44 (m, 2H), 3.86 (s, 3H), 4.97 (s, 1H), 6.83–6.88 (m, 1H), 7.21–7.34 (m, 4H), 7.44–7.50 (m, 1H), 7.71 (s, 1H), 7.91–8.18 (m, 1H), 11.45 (d, *J*=16.2 Hz, 1H) ppm; ^{13}C NMR (75 MHz, $CDCl_3$+DMSO–d_6) δ_C: 24.8, 25.2, 26.9, 27.3, 27.7, 39.1, 54.7, 107.4, 108.0, 108.8, 111.4, 112.3, 119.0, 121.2, 121.3, 121.7, 123.2, 125.8, 126.2, 126.5, 130.3, 133.5, 143.6, 144.5, 152.9 ppm; Anal. Calcd. for $C_{27}H_{23}BrClN_3O$: C, 62.02; H, 4.82; N, 8.04; found: C, 62.16; H, 4.96; N, 8.11.

2-(5-Bromo-1*H*-indol-3-yl)-4-(4-chloro-2-fluorophenyl)-5,6,7,8,9,10-hexahydrocycloocta[*b*]-pyridine-3-carbonitrile (16p)

Obtained as pale yellow solid (174.7 mg, 0.342 mmol, 90%) ^{1}H NMR (300 MHz, $CDCl_3$+DMSO–d_6) δ_H: 1.21–1.59 (m, 6H), 1.78–2.02 (m, 2H), 2.41–2.74 (m, 2H), 2.99–3.25 (m, 2H), 7.25–7.41 (m, 5H), 8.26 (d, *J*=2.7 Hz, 1H), 8.65 (s, 1H), 11.68 (br s, 1H) ppm; ^{13}C NMR (75 MHz, $CDCl_3$+DMSO–d_6) δc: 23.8, 24.4, 25.8, 28.6, 29.0, 34.2, 101.3, 110.7, 112.0, 112.1, 123.2, 123.4, 123.5, 126.4, 127.0, 129.0, 129.9, 133.6, 144.9, 152.1, 155.3, 158.7, 164.1 ppm; Anal. Calcd. for $C_{26}H_{20}BrClFN_3$: C, 61.37; H, 3.96; N, 8.26; found: C, 61.45; H, 3.86; N, 8.14.

2-(5-Bromo-1*H*-indol-3-yl)-4-(3,4,5-trimethoxyphenyl)-5,6,7,8,9,10-hexahydrocycloocta[*b*]-pyridine-3-carbonitrile (16t)

Obtained as pale yellow solid (198.0 mg, 0.361 mmol, 95%). ^{1}H NMR (300 MHz, $CDCl_3$+DMSO–d_6) δ_H: 1.29–1.66 (m, 6H), 1.78–2.02 (m, 2H), 2.55–2.76 (m, 2H), 3.00–3.18 (m, 2H), 3.81 (s, 9H), 6.43–6.62 (m, 2H), 7.23–7.37 (m, 2H), 8.15–8.25 (m, 1H), 8.57 (s, 1H), 11.54 (br s, 1H) ppm; ^{13}C NMR (75 MHz, $CDCl_3$+DMSO–d_6) δ_C: 24.3, 24.7, 26.0, 29.1, 29.9, 34.2, 54.7, 59.0, 101.6, 104.2, 111.1, 112.0, 112.1, 116.8, 123.3, 123.4, 126.7, 127.0, 128.8, 130.8, 133.8, 136.4, 151.6, 152.0, 152.2, 163.7 ppm; Anal. Calcd. for $C_{29}H_{28}BrN_3O_3$: C, 63.74; H, 5.16; N, 7.69; found: C, 63.88; H, 5.21; N, 7.75.

2-(1*H*-Indol-3-yl)-4-(*p*-tolyl)-6,7,8,9-tetrahydro-5H-cyclohepta[*b*]pyridine-3-carbonitrile (17b)

Obtained as white solid (163.9 mg, 0.434 mmol, 80%). ^{1}H NMR (300 MHz, $CDCl_3$) δ_H: 1.51–2.01 (m, 6H), 2.45 (s, 3H), 2.59–2.74 (m, 2H), 3.20–3.35 (m, 2H), 7.21–7.40 (m, 7H), 8.14 (d, *J*=3.0 Hz, 1H), 8.43–8.54 (m, 1H), 8.66 (br s, 1H) ppm; ^{13}C NMR (75 MHz,

$CDCl_3$) δ_C: 21.4, 26.4, 27.7, 29.8, 32.1, 40.1, 103.5, 111.3, 121.2, 122.3, 122.9, 126.5, 126.7, 128.5, 129.4, 132.6, 134.0, 136.3, 138.5, 153.3, 153.8, 167.3 ppm; Anal. Calcd. for $C_{26}H_{23}N_3$: C, 82.73; H, 6.14; N, 11.13; found: C, 82.79; H, 6.26; N, 11.20.

4-(4-Chlorophenyl)-2-(1*H*-indol-3-yl)-6,7,8,9-tetrahydro-5*H*-cyclohepta[*b*]pyridine-3-carbonitrile (17f)

Obtained as white solid (177.1 mg, 0.445 mmol, 82%). ^{1}H NMR (300 MHz, $CDCl_3$) δ_H: 1.72–2.04 (m, 6H), 2.48–2.75 (m, 2H), 3.15–3.41 (m, 2H), 7.25–7.30 (m, 4H), 7.32–7.40 (m, 1H), 7.51 (d, *J*=8.4 Hz, 2H), 8.15 (d, *J*=2.7 Hz, 1H), 8.45–8.53 (m, 1H), 8.69 (br s, 1H) ppm; ^{13}C NMR (75 MHz, $CDCl_3$) δ_C: 26.2, 27.6, 29.7, 32.0, 40.0, 102.9, 111.4, 113.7, 121.2, 122.0, 122.9, 126.2, 126.8, 129.0, 130.0, 134.9, 135.2, 136.2, 151.8, 153.9, 167.6 ppm; Anal. Calcd. for $C_{25}H_{20}ClN_3$: C, 75.46; H, 5.07; N, 10.56; found: C, 75.51; H, 5.16; N, 10.42.

4-(2-Bromophenyl)-2-(1*H*-indol-3-yl)-4,5,6,7,8,9-hexahydro-1*H*-cyclohepta[*b*]pyridine-3-carbonitrile (17l)

Obtained as yellow solid (214.7 mg, 0.483 mmol, 89%). ^{1}H NMR (300 MHz, $CDCl_3$+DMSO–d_6) δ_H: 0.96–1.19 (m, 1H), 1.33–2.14(m, 7H), 2.19–2.44 (m, 2H), 4.84 (s, 1H), 7.03–7.14 (m, 3H), 7.30–7.38 (m, 2H), 7.46 (d, *J*=8.1 Hz, 1H), 7.51 (s, 1H), 7.59 (t, *J*=9.0 Hz, 3H), 11.08 (br s, 1H) ppm; ^{13}C NMR (75 MHz, $CDCl_3$+DMSO–d_6) δ_C: 24.7, 25.8, 30.2, 30.7, 30.8, 45.9, 76.0, 107.9, 110.6, 110.9, 118.9, 119.1, 120.9, 121.2, 121.6, 124.3, 125.3, 127.1, 127.2, 133.7, 135.1, 143.9, 144.6 ppm; Anal. Calcd. for $C_{25}H_{22}BrN_3$; C, 67.57; H, 4.99; N, 9.46; found: C, 67.64; H, 5.10; N, 9.36.

2-(1*H*-Indol-3-yl)-4-(4-(methylthio)phenyl)-6,7,8,9-tetrahydro-5*H*-cyclohepta[*b*]pyridine-3-carbonitrile (17v)

Obtained as yellow solid (186.7 mg, 0.456 mmol, 84%). ^{1}H NMR (300 MHz, $CDCl_3$) δ_H: 1.70–2.03 (m, 6H), 2.55 (s, 3H), 2.57–2.72 (m, 2H), 3.19–3.35 (m, 2H), 7.22–7.41 (m, 7H), 8.09–8.22 (m, 1H), 8.41–8.54 (m, 1H), 8.60–8.72 (br s, 1H) ppm; ^{13}C NMR (75 MHz, $CDCl_3$) δ_C: 15.3, 26.3, 27.7, 29.7, 32.1, 40.0, 103.3, 111.3, 113.9, 121.2, 122.9, 126.1, 126.1, 126.3, 126.8, 129.0, 132.7, 133.2, 136.2, 139.6, 152.6, 153.8, 167.4 ppm; Anal. Calcd. for $C_{26}H_{23}N_3S$; C, 76.25; H, 5.66; N, 10.26; found: C, 76.33; H, 5.70; N, 10.20.

2-(1*H*-Indol-3-yl)-4-phenyl-5,6,7,8-tetrahydroquinoline-3-carbonitrile (18a)

Obtained as yellow solid (153.6 mg, 0.439 mmol, 81%;). ^{1}H NMR (300 MHz, $CDCl_3$) δ_H: 1.66–1.84 (m, 2H), 1.87–2.07 (m, 2H), 2.50 (t, *J*=6.3 Hz, 2H), 3.18 (t, *J*=6.3 Hz, 2H), 7.25–7.38 (m, 2H), 7.30 – 738 (m, 3H), 7.50–7.59 (m, 3H), 8.13 (d, *J*=2.7 Hz, 1H), 8.42–8.52 (m, 1H), 8.72 (br s, 1H) ppm; ^{13}C NMR (75 MHz, $CDCl_3$) δ_C: 23.0, 23.1, 27.5, 34.1, 103.7, 111.7, 121.6, 122.5, 123.4, 127.2, 127.5, 128.6, 129.2, 136.7, 154.4, 155.0, 161.8 ppm; Anal. Calcd. for $C_{24}H_{19}N_3$: C, 82.49; H, 5.48; N, 12.03; found: C, 82.56; H, 5.42; N, 12.05.

2-(1*H*-Indol-3-yl)-4-(*p*-tolyl)-5,6,7,8-tetrahydroquinoline-3-carbonitrile (18b)

Obtained as pale yellow solid (157.8 mg, 0.434 mmol, 80%). ^{1}H NMR (300 MHz, $CDCl_3$) δ_H: 1.73–1.84 (m, 2H), 2.40 (s, 2H), 2.45 (s, 3H), 2.61 (t, *J*=6.0 Hz, 2H), 2.96 (t, *J*=6.0 Hz, 2H), 7.23–7.48 (m, 6H), 8.24 (d, *J*=2.7 Hz, 1H), 8.39 (br s, 1H), 8.52–8.62 (m, 2H) ppm; ^{13}C NMR (75 MHz, $CDCl_3$) δ_C: 21.8, 21.9, 22.9, 28.2, 28.4, 103.9, 111.7, 121.7, 122.5, 123.4, 127.3, 128.7, 128.7, 129.4, 129.9, 130.3, 131.6, 135.0, 136.6, 154.1,

159.4, 156.0 ppm; Anal. Calcd. for $C_{25}H_{21}N_3$: C, 82.61; H, 5.82; N, 11.56; found: C, 82.76; H, 5.90; N, 11.60.

4-(4-Chlorophenyl)-2-(1*H*-indol-3-yl)-5,6,7,8-tetrahydroquinoline-3-carbonitrile (18f)

Obtained as white solid (166.7 mg, 0.434 mmol, 80%). ^{1}H NMR (300 MHz, $CDCl_3$) δ_H: 1.74–1.85 (m, 2H), 1.88–2.02 (m, 2H), 2.47 (t, *J*=6.3 Hz, 2H), 3.16 (t, *J*=6.3 Hz, 2H), 7.26–7.43 (m, 5H), 7.51 (d, *J*=8.4 Hz, 2H), 8.15 (d, *J*=3.0 Hz, 1H), 8.43–8.52 (m, 1H), 8.66 (br s, 1H) ppm; ^{13}C NMR (75 MHz, $CDCl_3$) δ_C: 22.5, 22.7, 27.1, 33.7, 102.9, 111.3, 114.1, 121.3, 122.3, 123.1, 126.7, 129.2, 129.3, 129.7, 134.7, 135.1, 136.3, 153.2, 161.7 ppm; Anal. Calcd. for $C_{24}H_{18}ClN_3$: C, 75.09; H, 4.73; N, 10.95; found: C, 75.15; H, 4.77; N, 10.98.

2-(1*H*-Indol-3-yl)-4-(4-(methylthio)phenyl)-5,6,7,8-tetrahydro-quinoline-3-carbonitrile(18v)

Obtained as yellow solid (182.5 mg, 0.461 mmol, 85%). ^{1}H NMR (300 MHz, $CDCl_3$) δ_H: 1.67–2.05 (m, 4H), 2.41–2.67 (m, 5H), 3.07–3.28 (m, 2H), 7.15–7.49 (m, 6H), 7.67 (d, *J*=7.8 Hz, 1H), 8.16 (s, 1H), 8.46 (s, 1H), 8.69 (s, 1H) ppm; ^{13}C NMR (75 MHz, $CDCl_3$) δ_C: 15.3, 22.5, 22.7, 27.1, 33.6, 103.1, 111.3, 121.2, 122.0, 122.9, 126.0, 126.1, 126.3, 126.7, 126.8, 128.7, 129.9, 132.1, 136.2, 153.9, 154.0, 161.5 ppm; Anal. Calcd. for $C_{25}H_{21}N_3S$: C, 75.92; H, 5.35; N, 10.62; found: C, 75.81; H, 5.28; N, 10.60.

Copies of NMR spectra

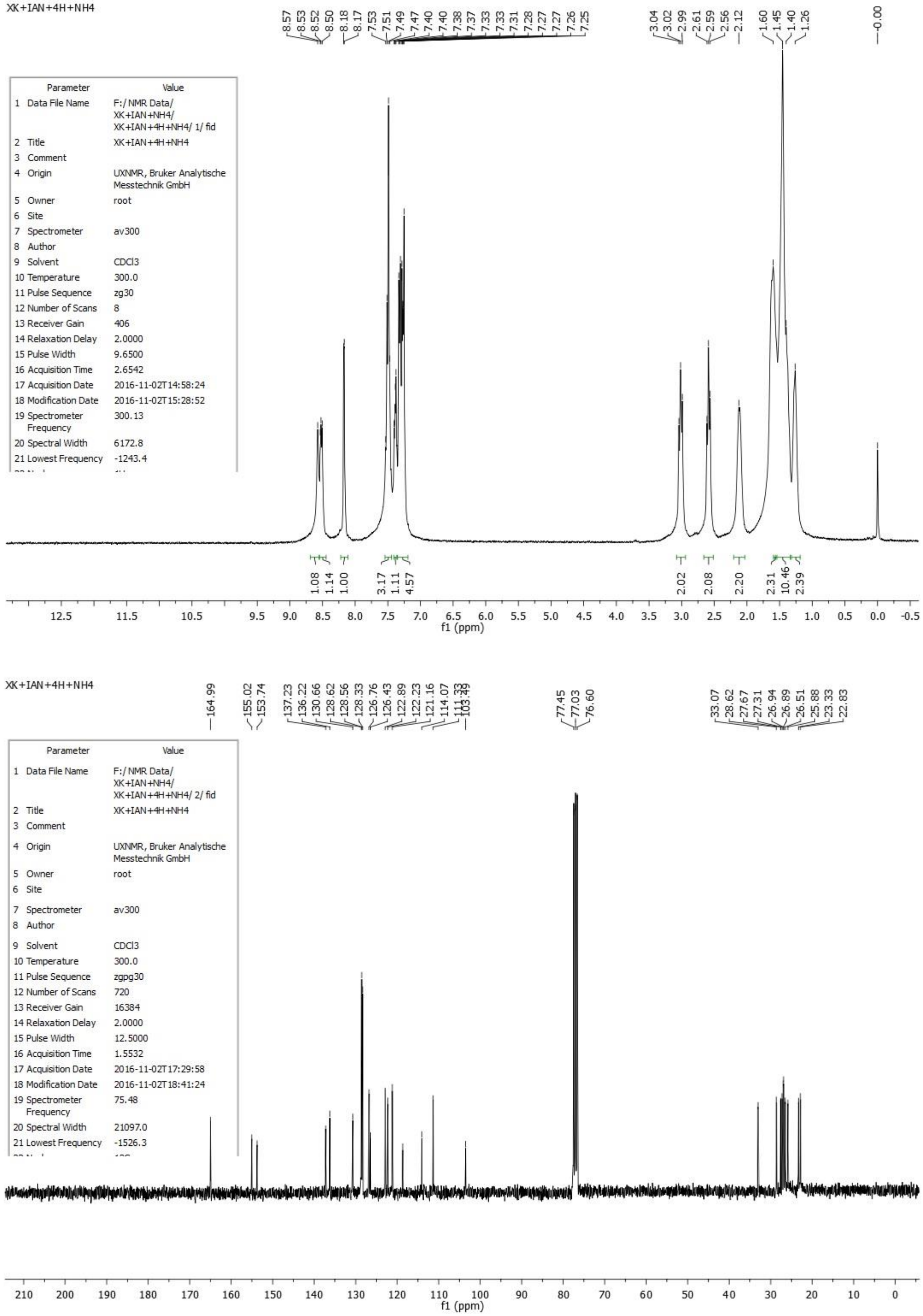

Figure 1: ^{1}H and ^{13}C NMR spectra of **7a**

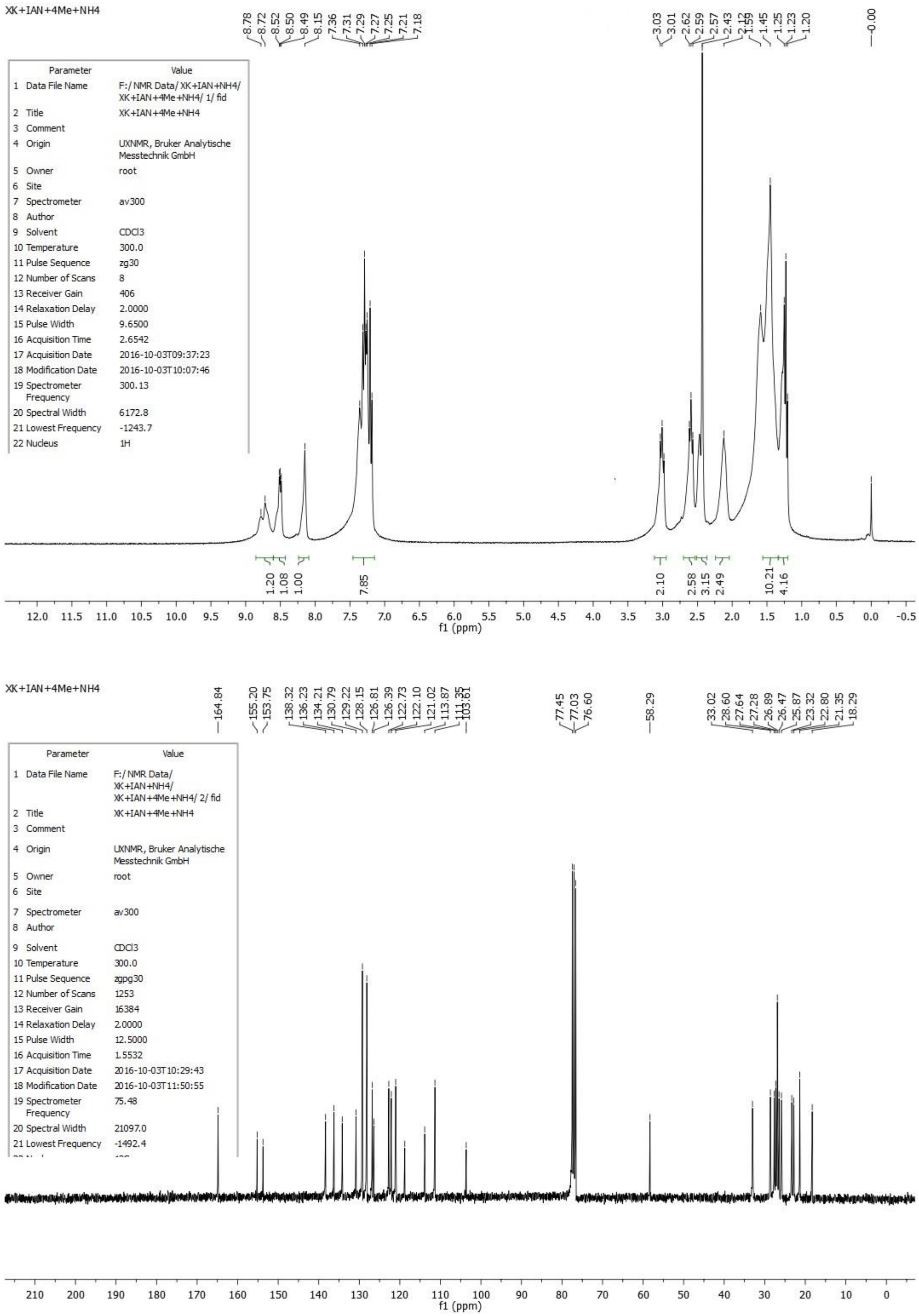

Figure 2: ^{1}H and ^{13}C NMR spectra of **7b**

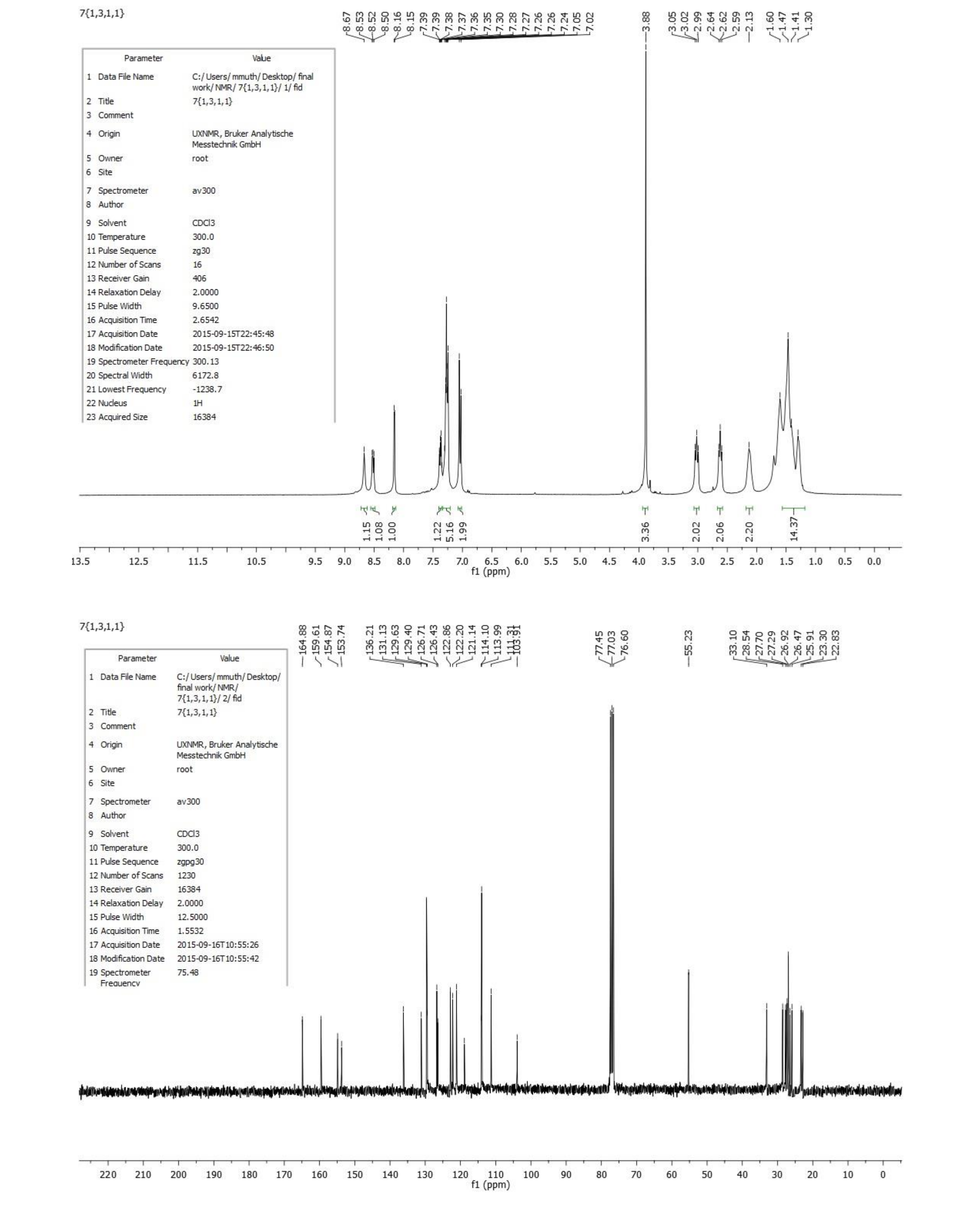

Figure 3: ^{1}H and ^{13}C NMR spectra of **7c**

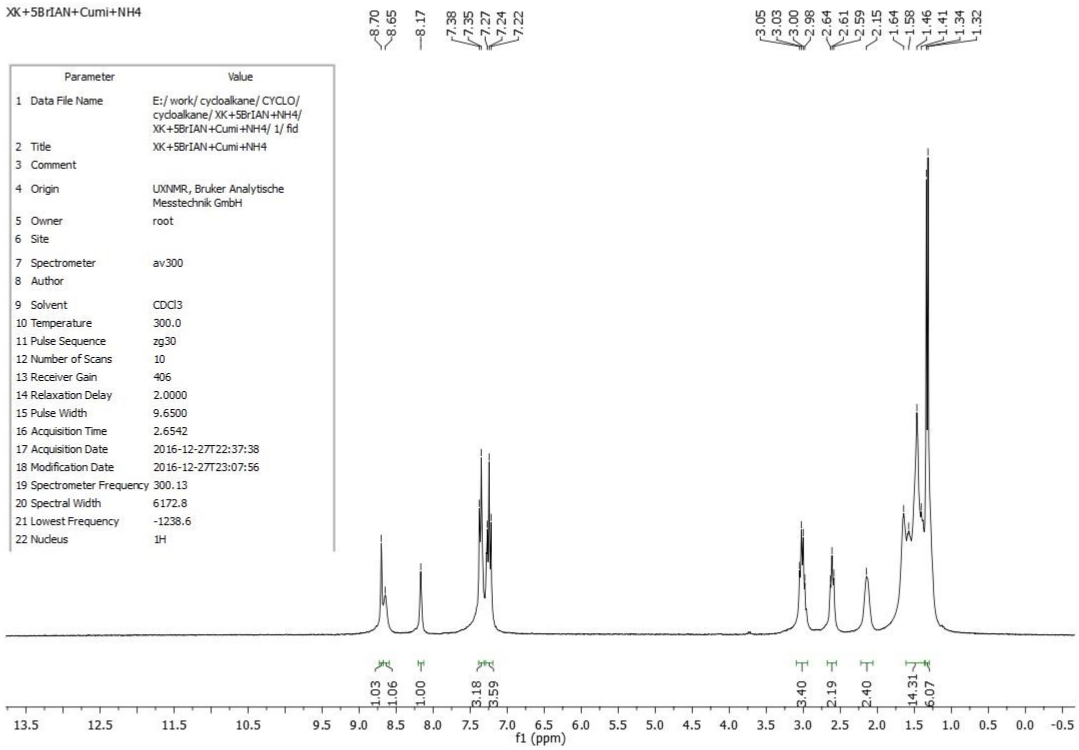

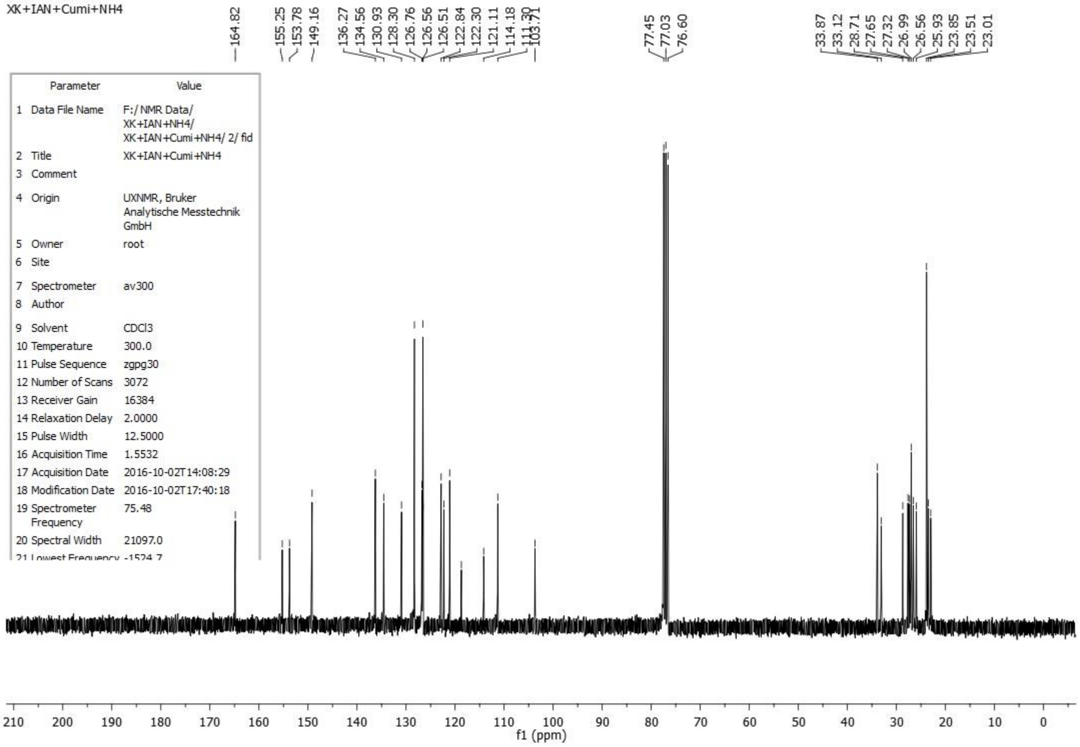

Figure 4: ^{1}H and ^{13}C NMR spectra of **7d**

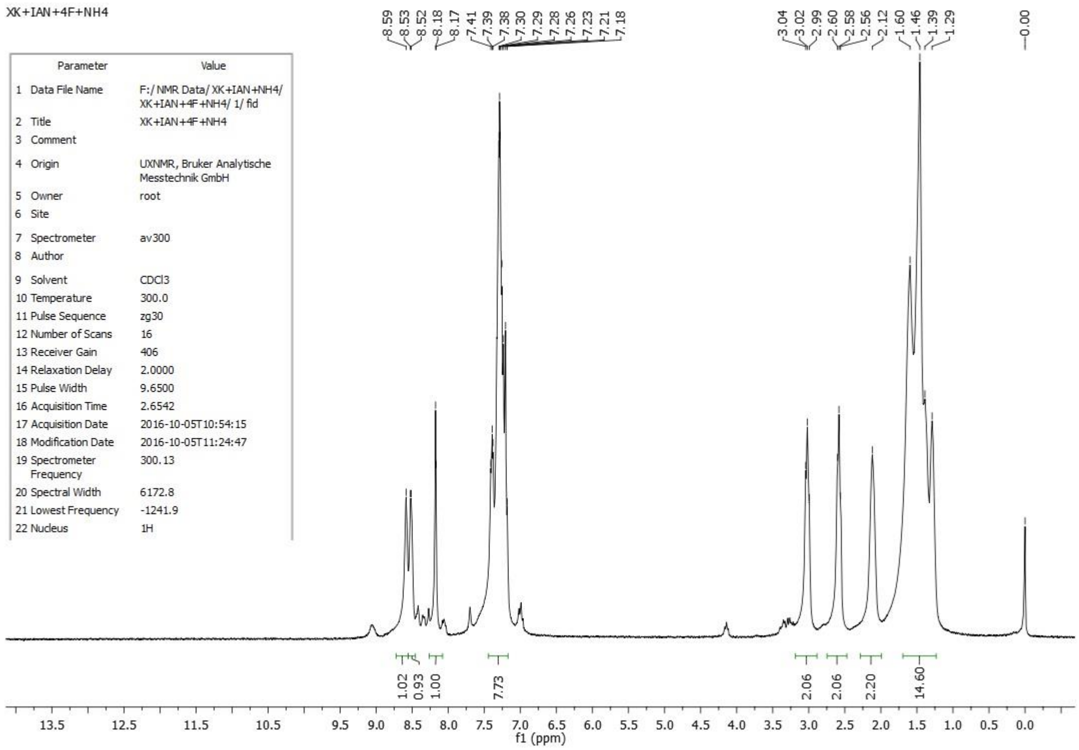

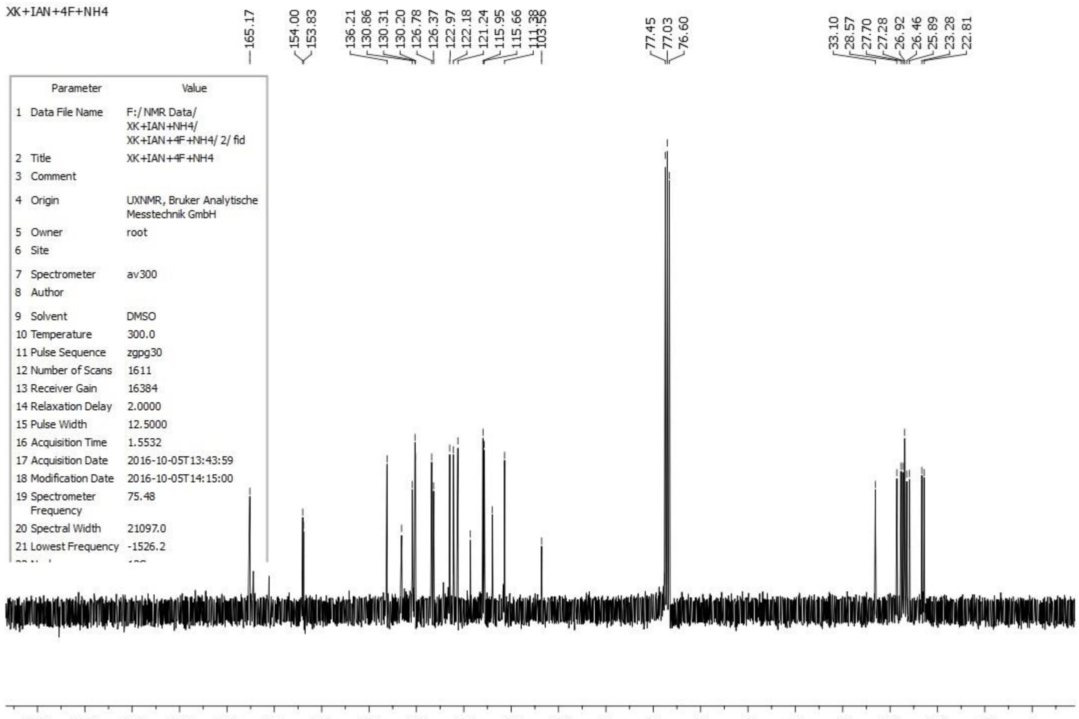

Figure 5: ^{1}H and ^{13}C NMR spectra of **7e**

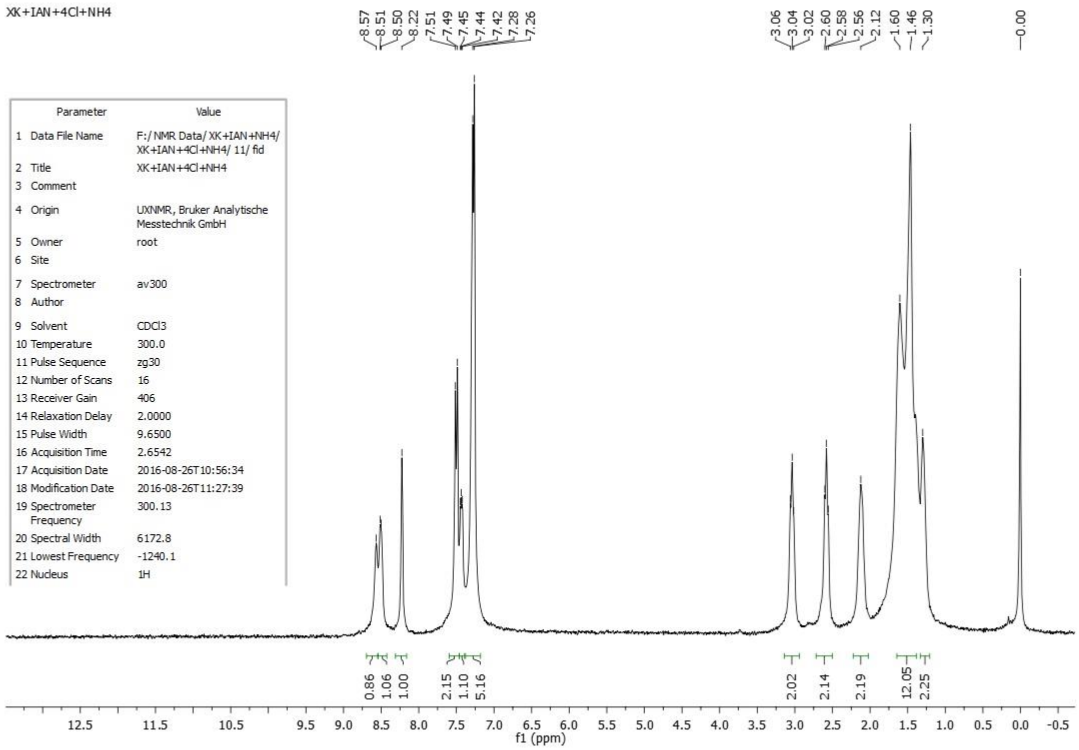

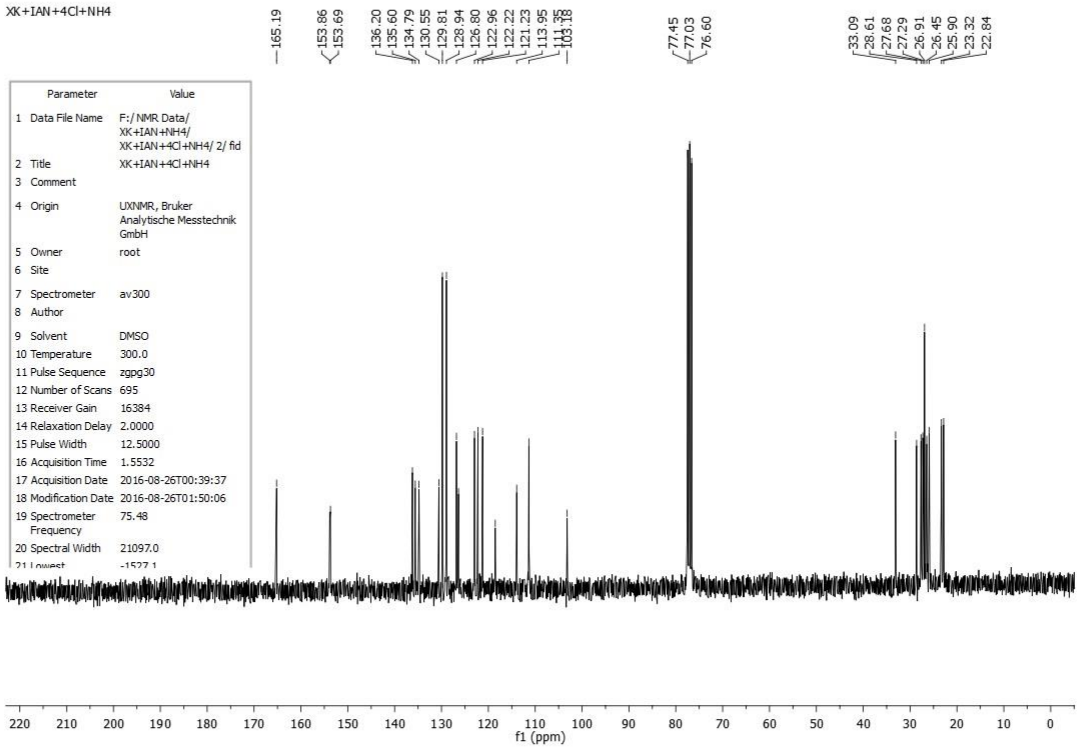

Figure 6: ^{1}H and ^{13}C NMR spectra of **7f**

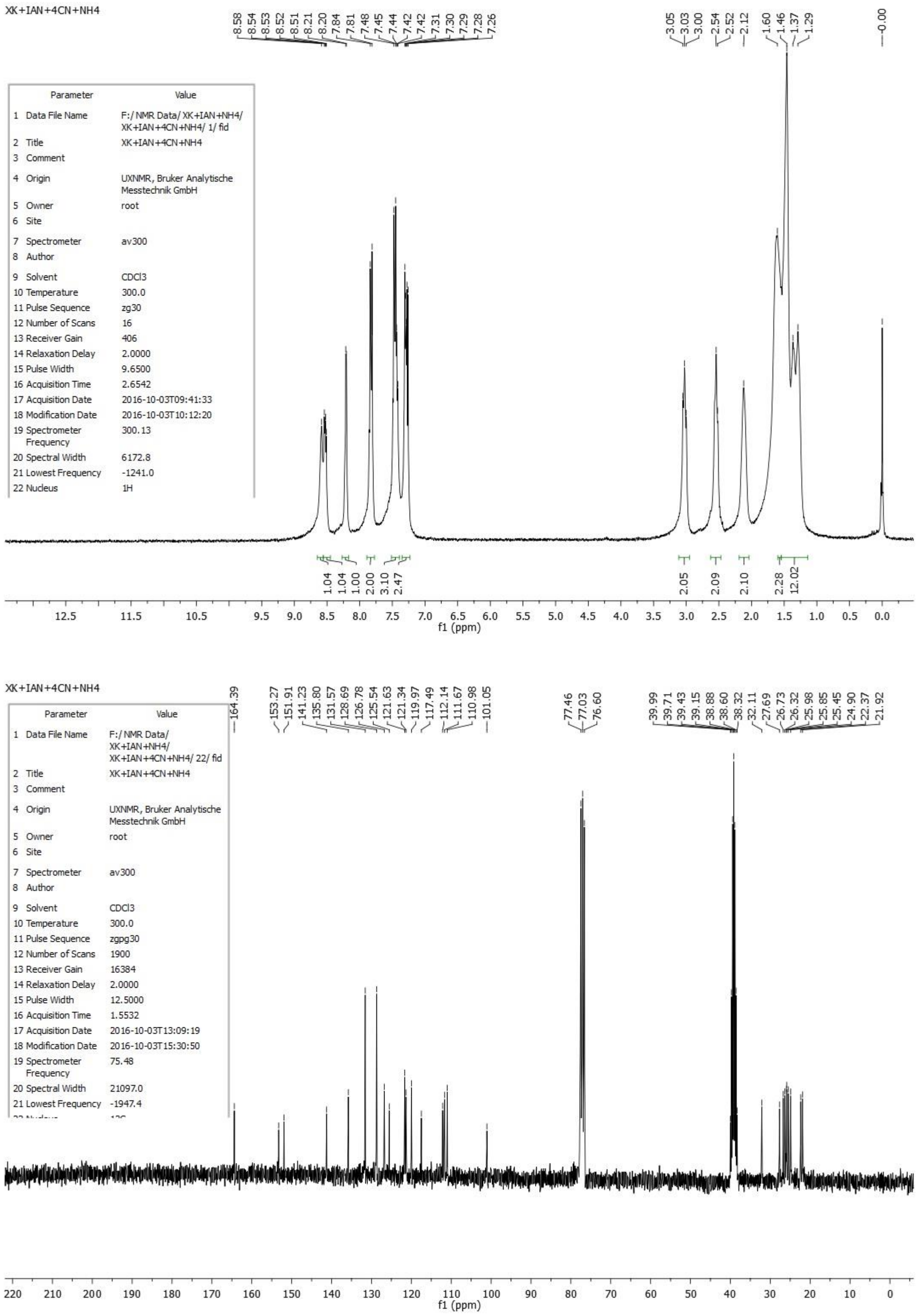

Figure 7: ^{1}H and ^{13}C NMR spectra of **7h**

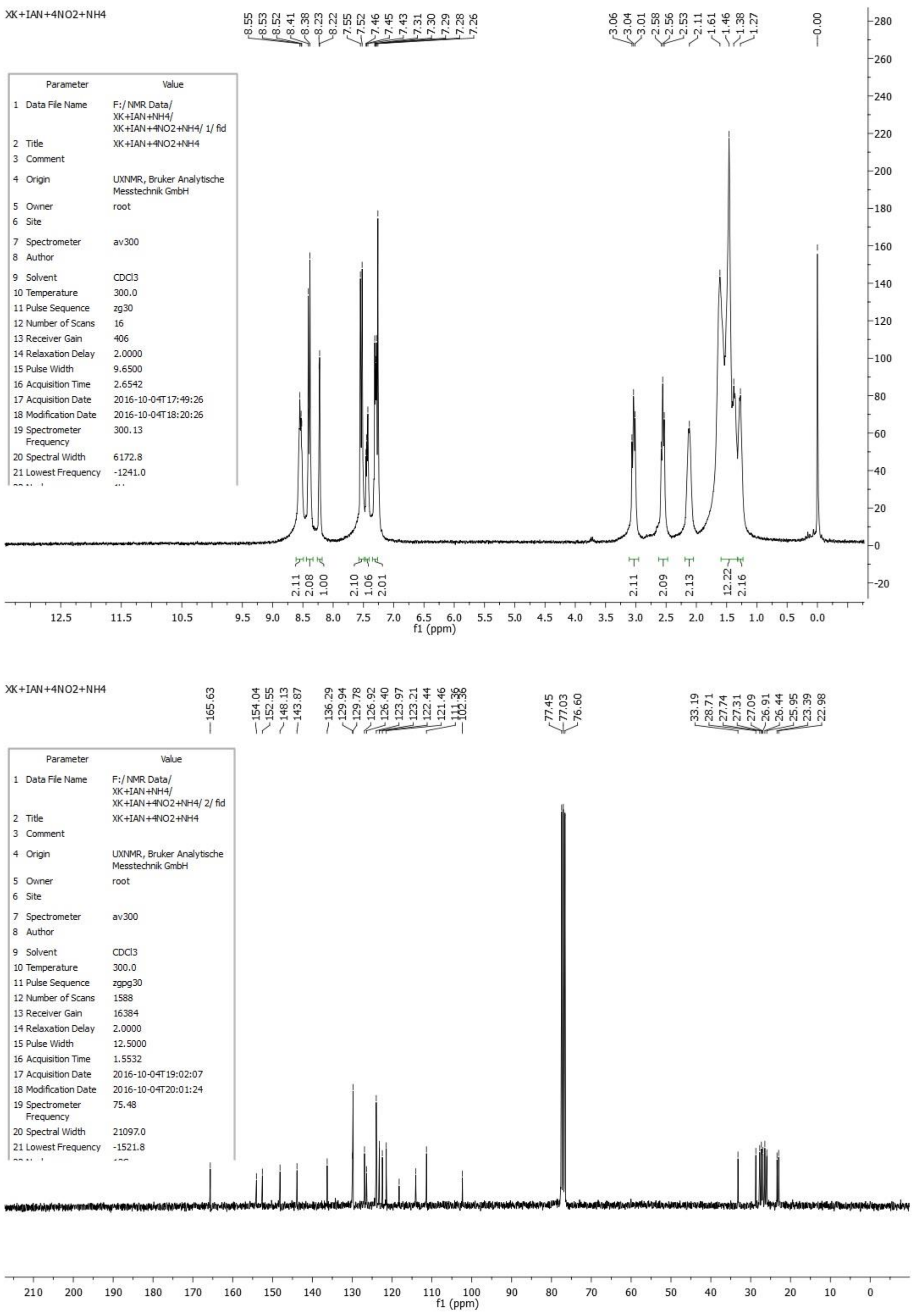

Figure 8: ^{1}H and ^{13}C NMR spectra of **7i**

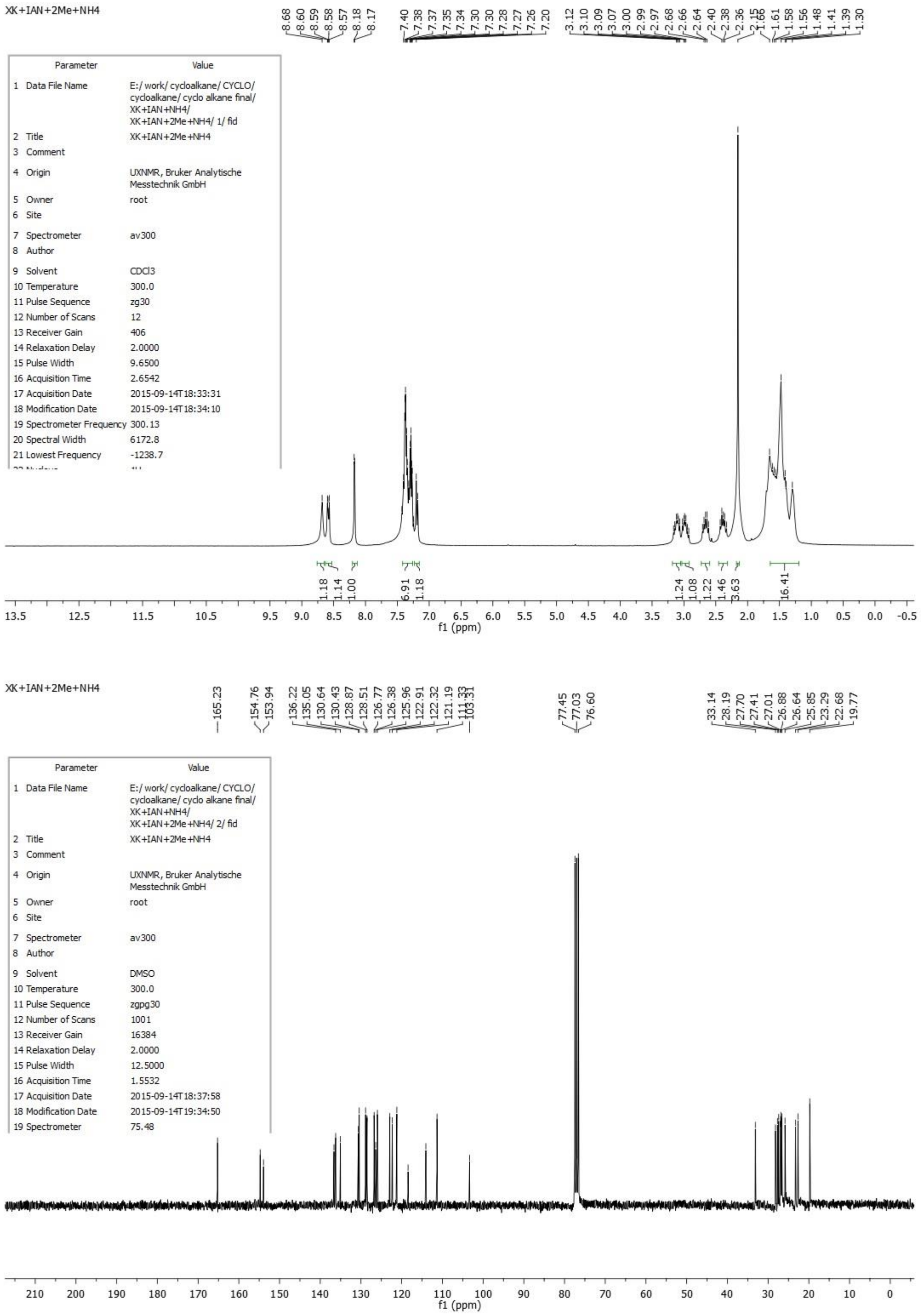

Figure 9: ^{1}H and ^{13}C NMR spectra of **7j**

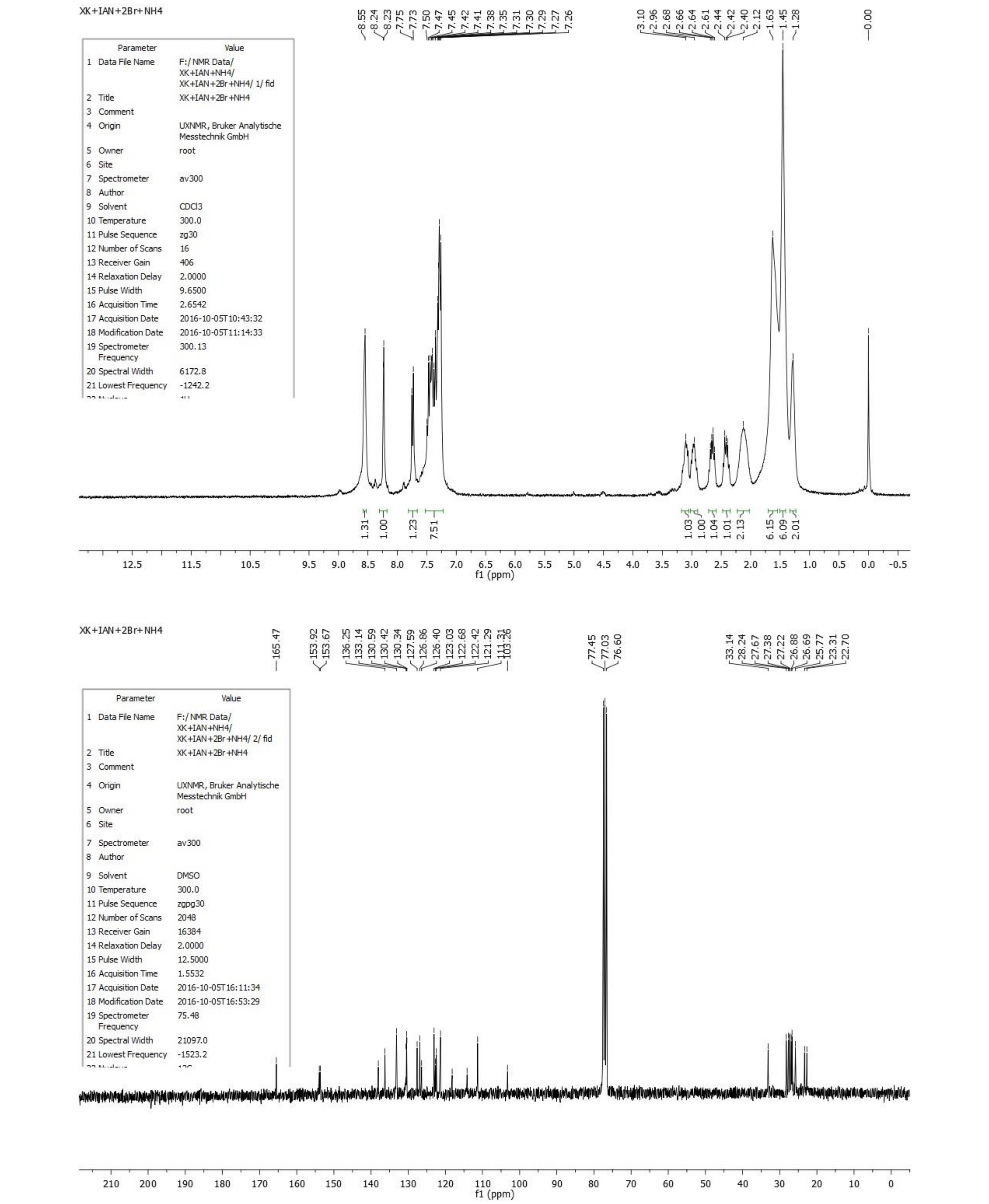

Figure 10: ^{1}H and ^{13}C NMR spectra of **7l**

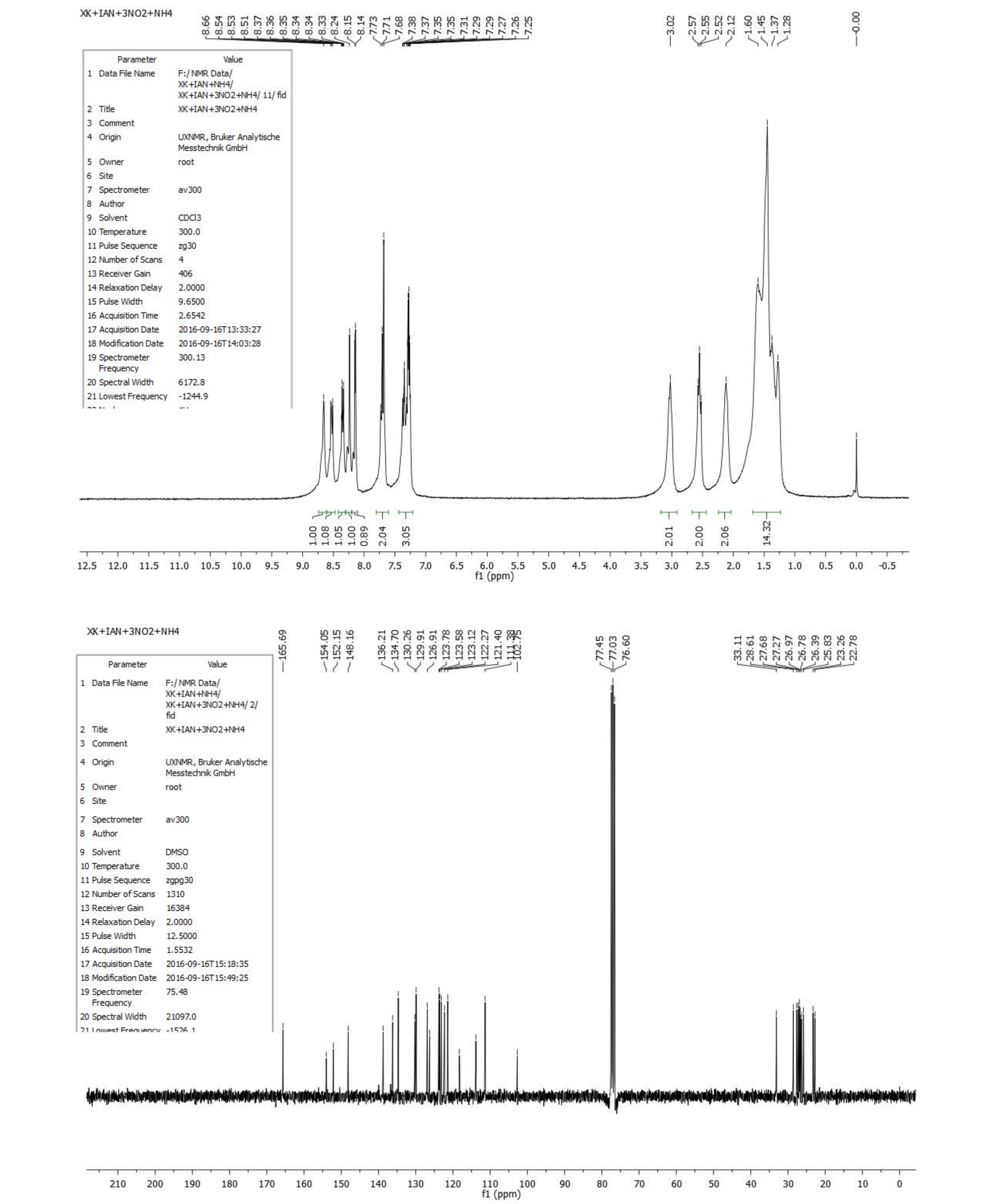

Figure 11: ^{1}H and ^{13}C NMR spectra of **7m**

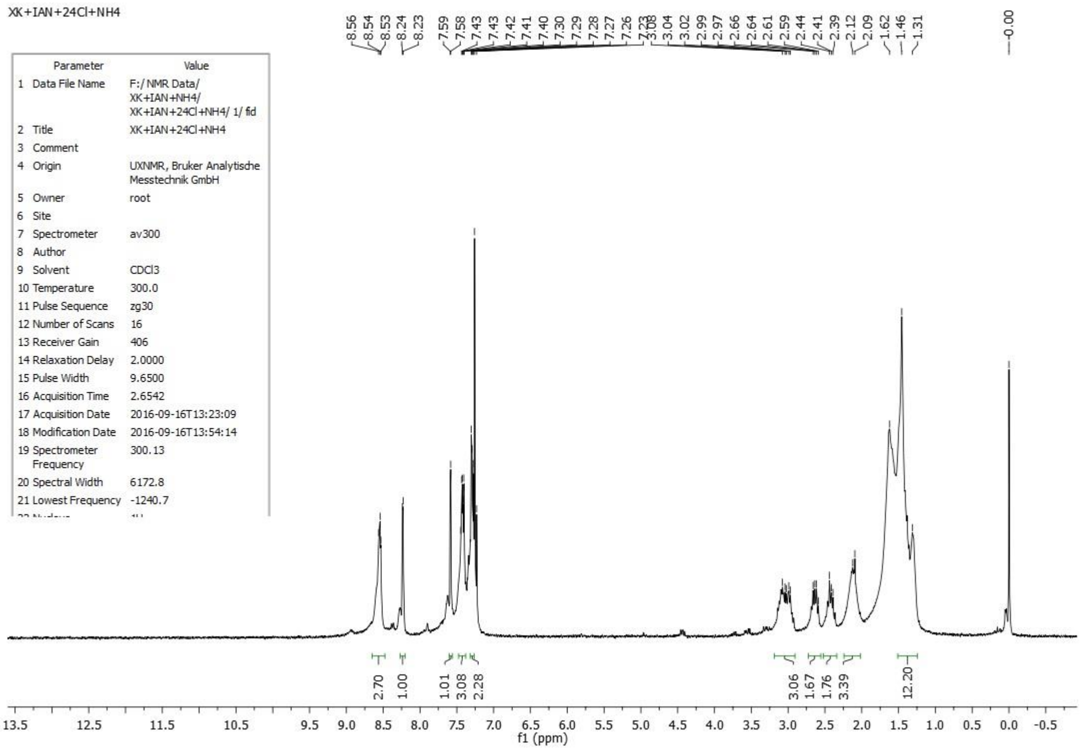

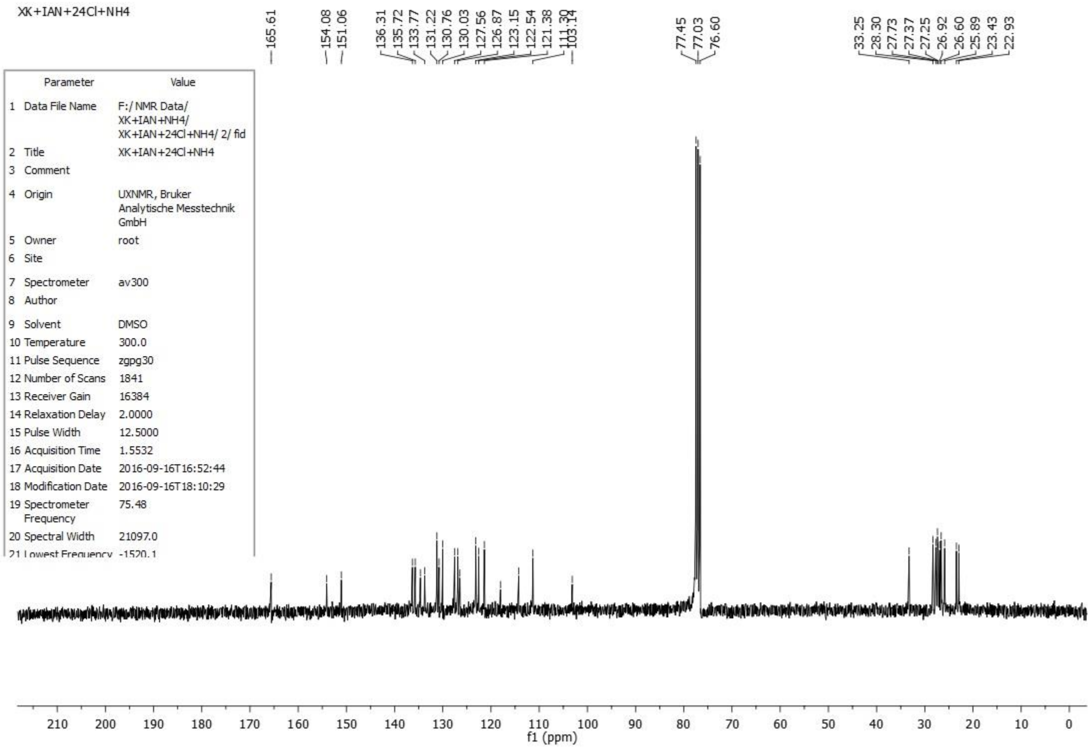

Figure 12: ^{1}H and ^{13}C NMR spectra of **7n**

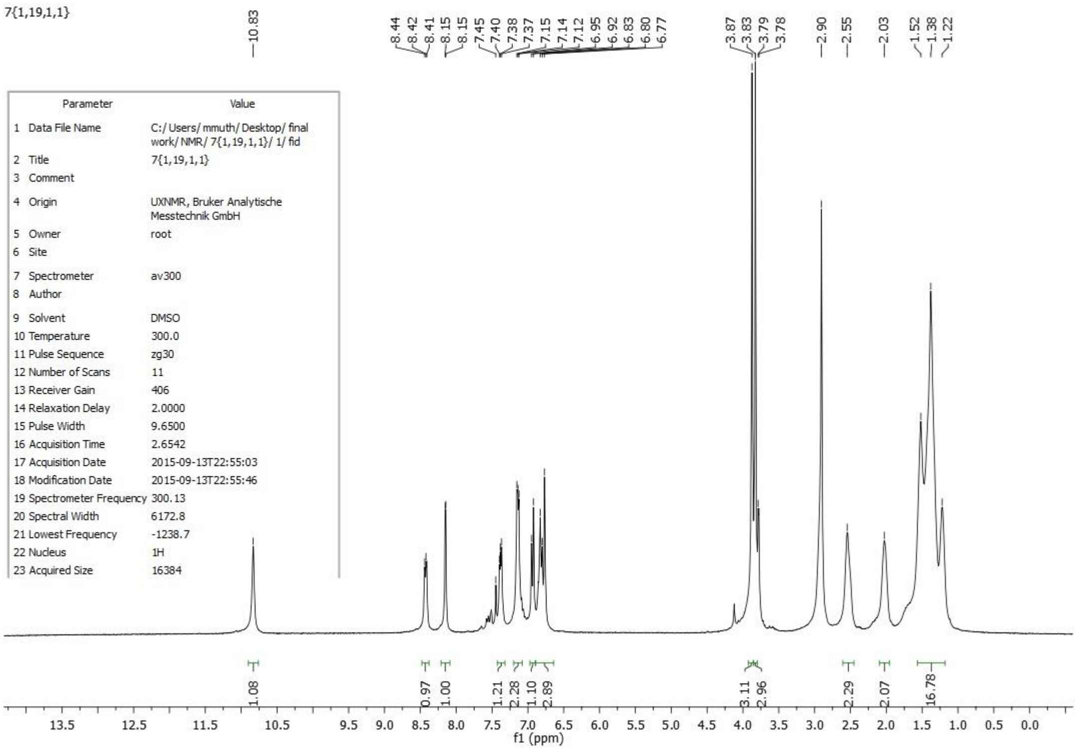

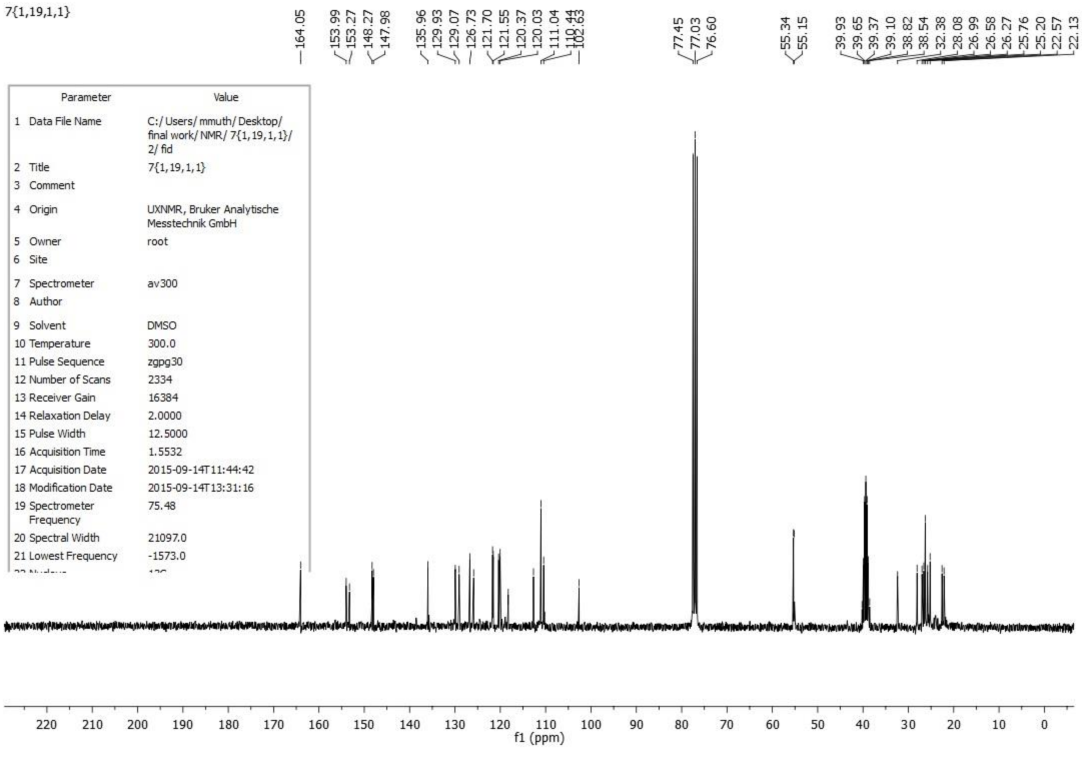

Figure 13: ^{1}H and ^{13}C NMR spectra of **7s**

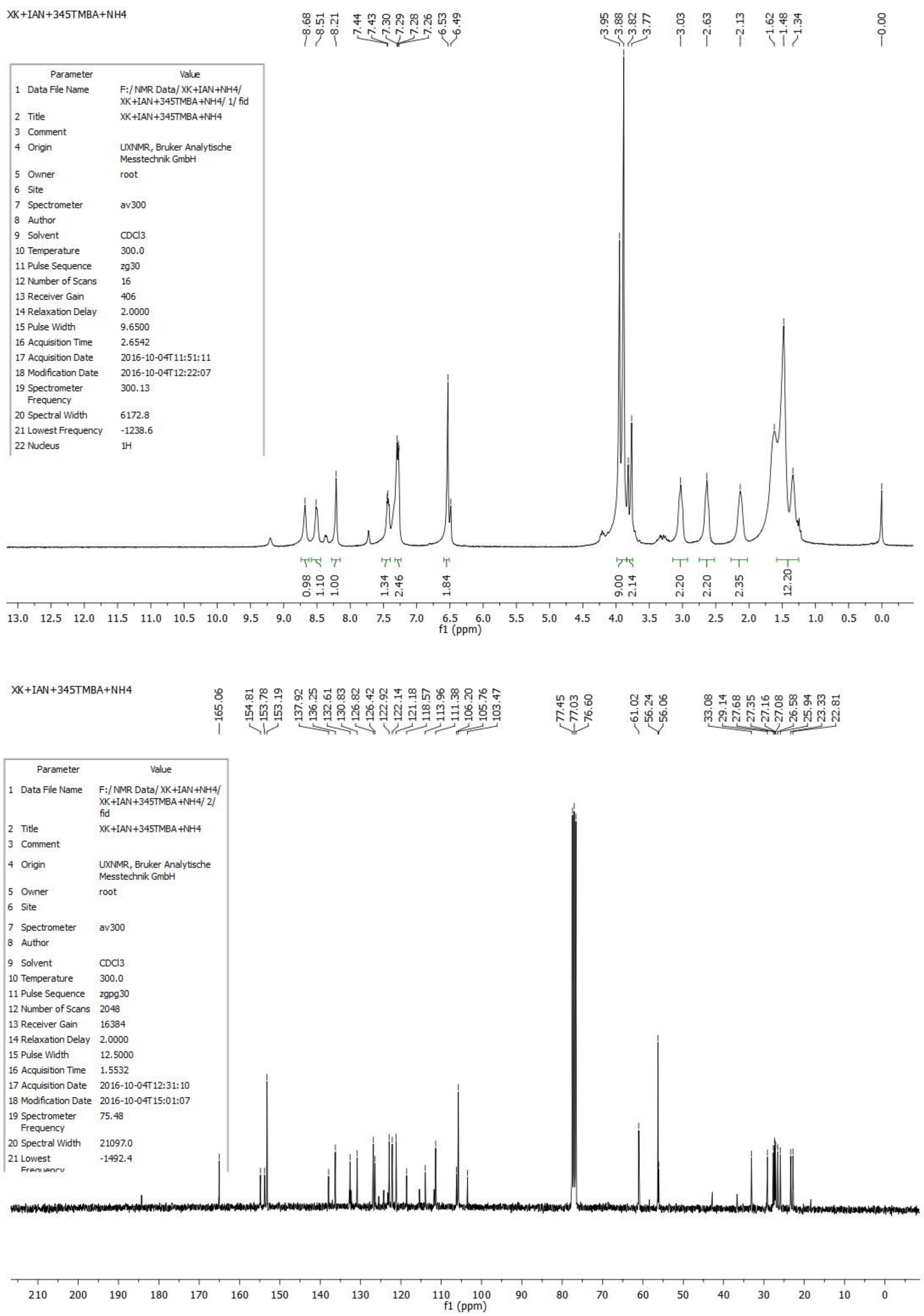

Figure 14: ^{1}H and ^{13}C NMR spectra of **7t**

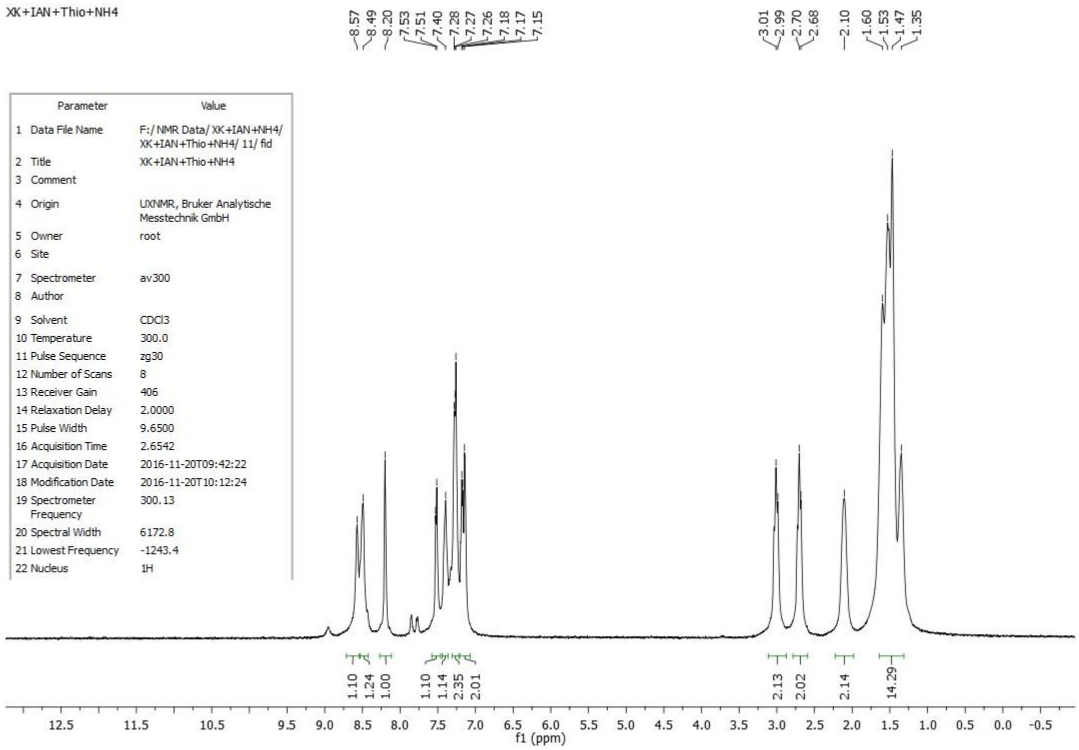

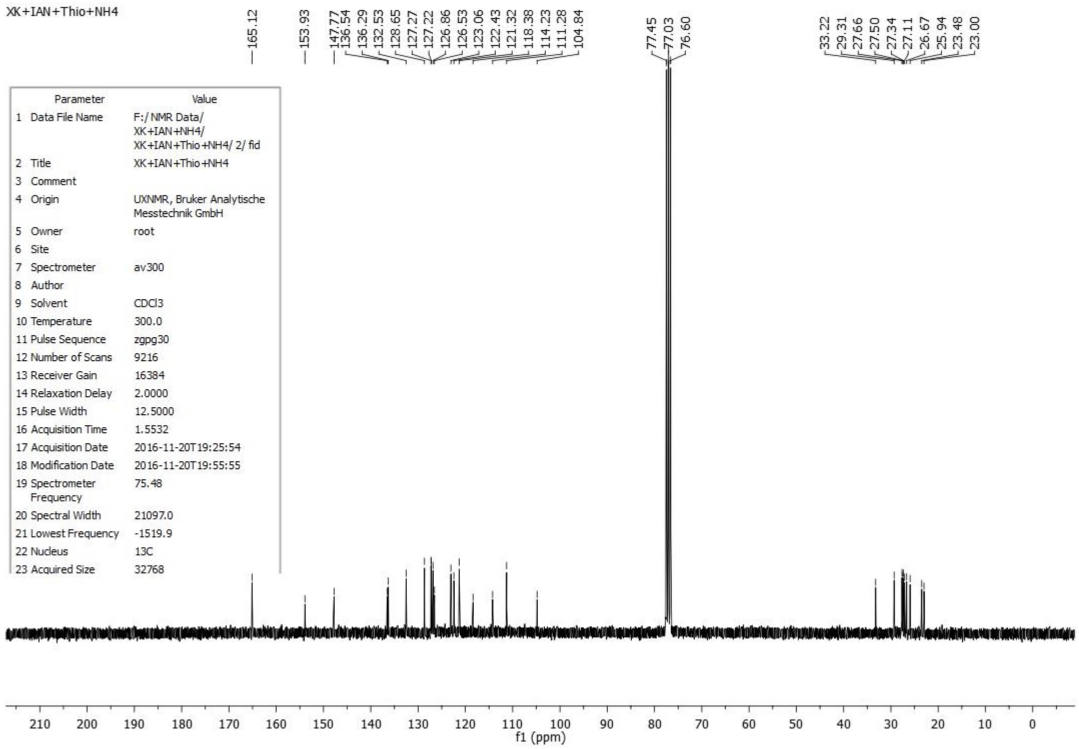

Figure 15: ^{1}H and ^{13}C NMR spectra of **7u**

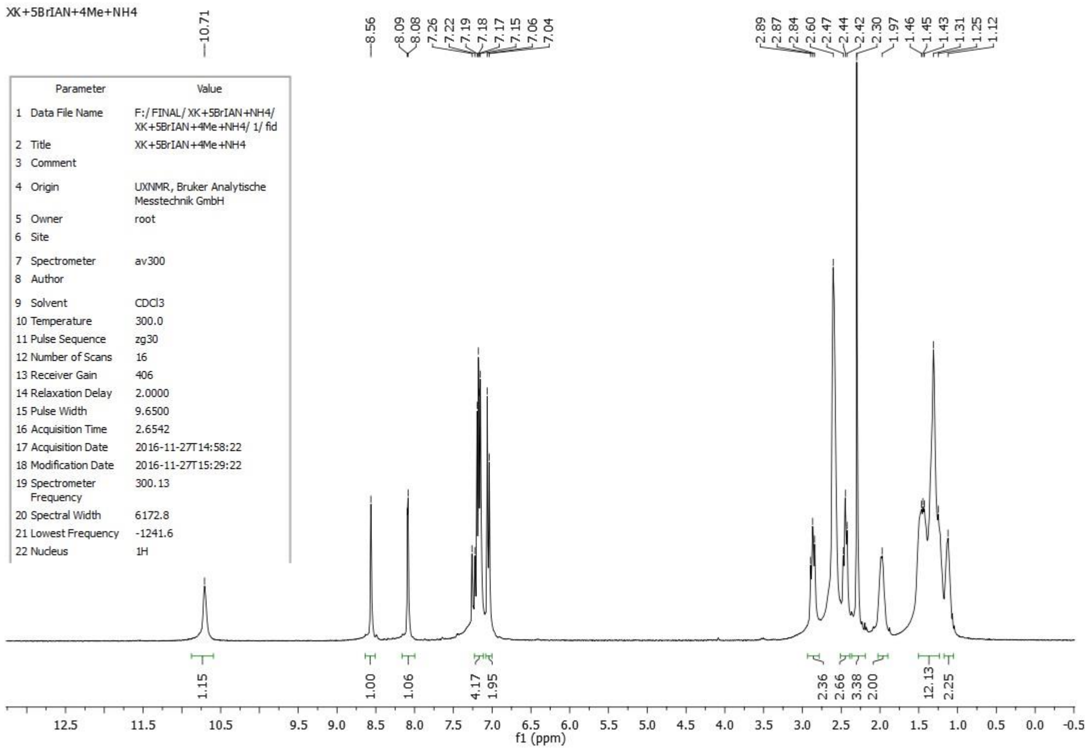

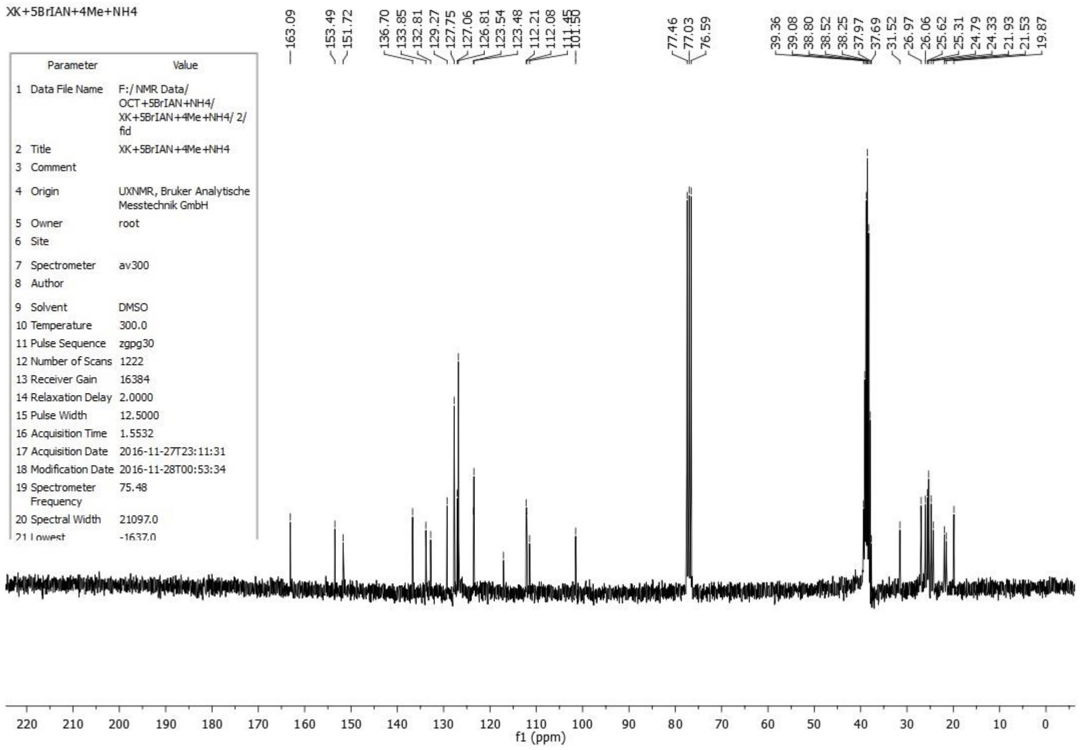

Figure 16: ^{1}H and ^{13}C NMR spectra of **14b**

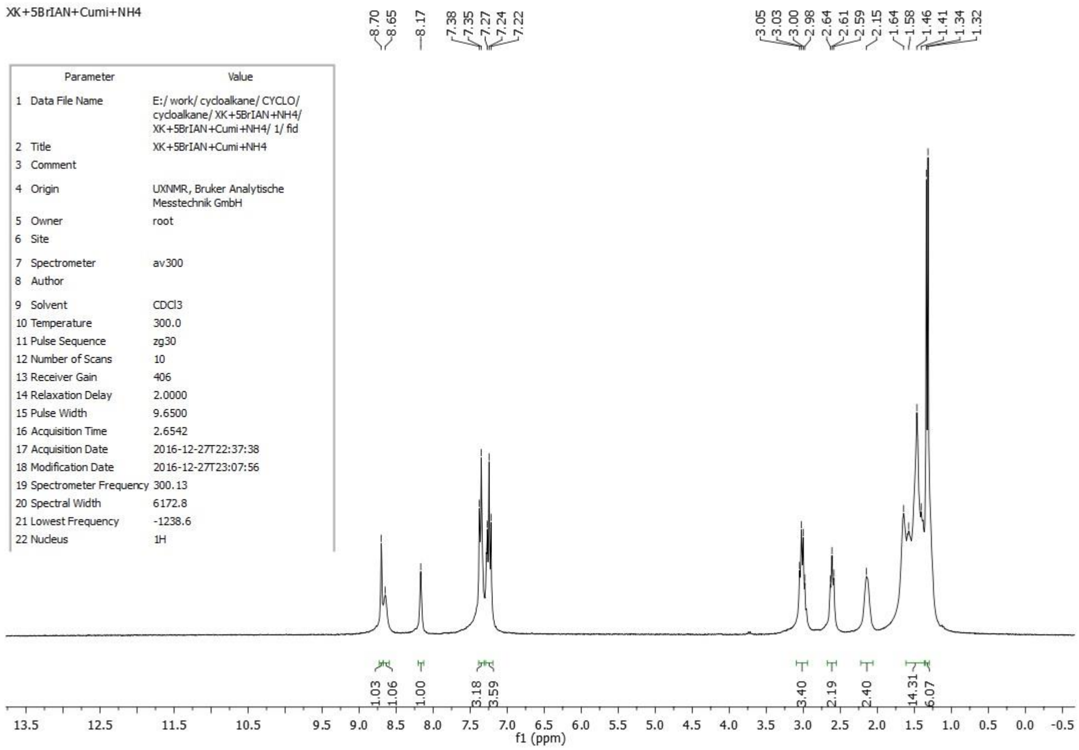

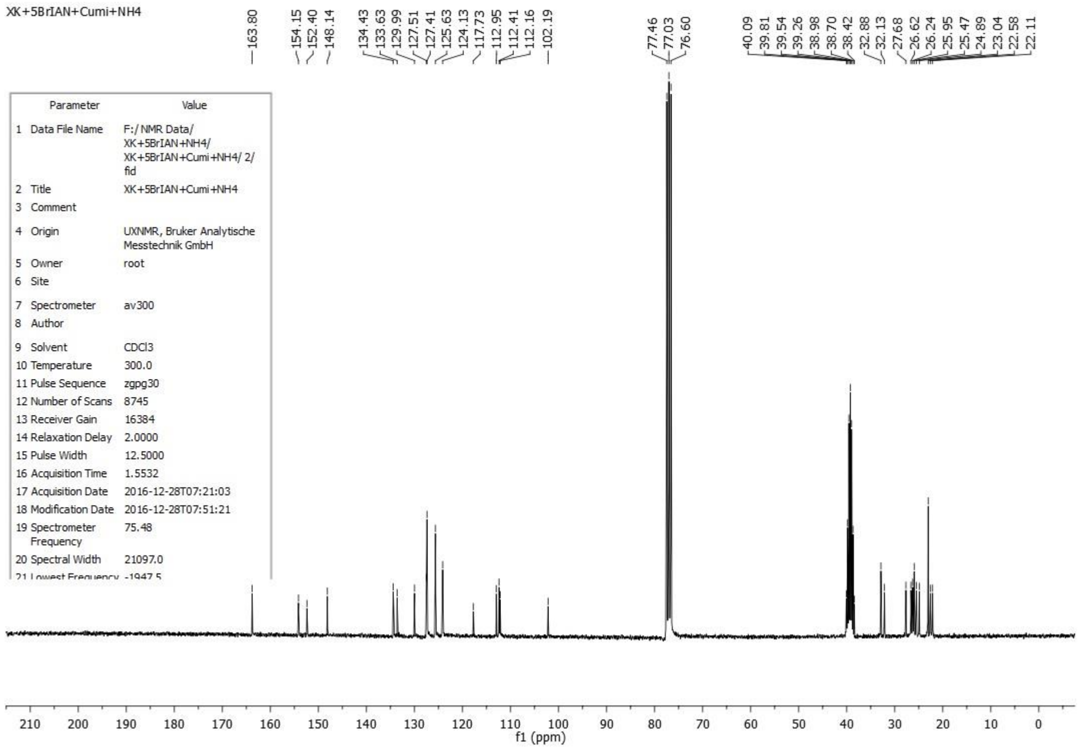

Figure 17: ^{1}H and ^{13}C NMR spectra of **14d**

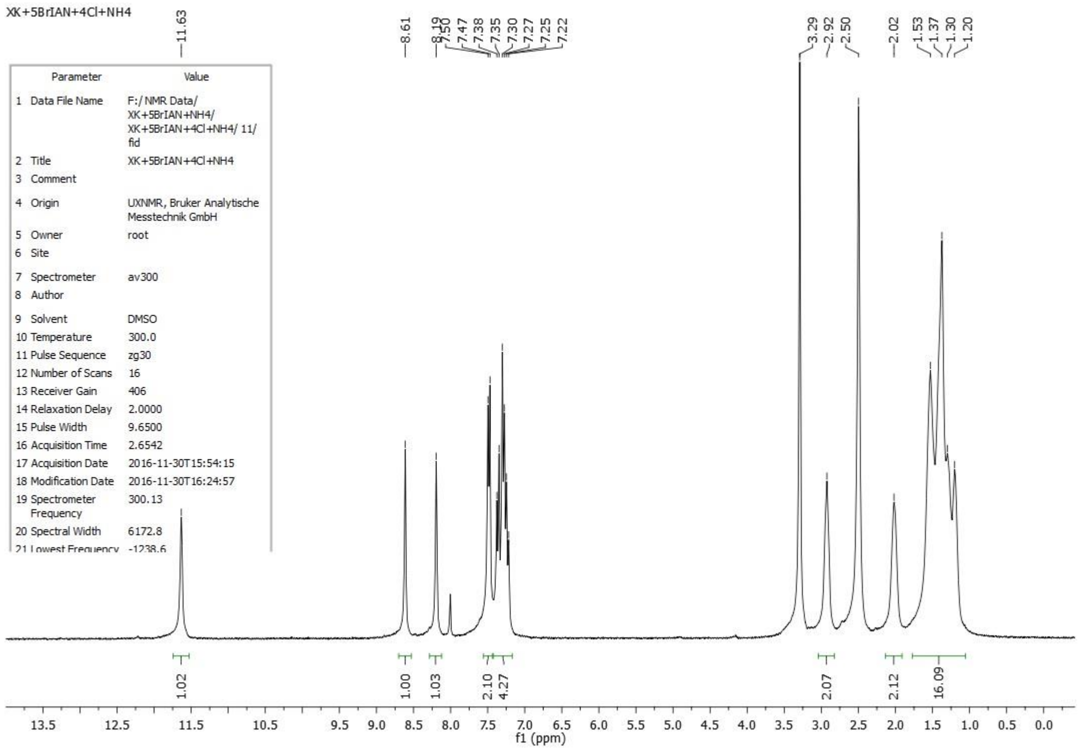

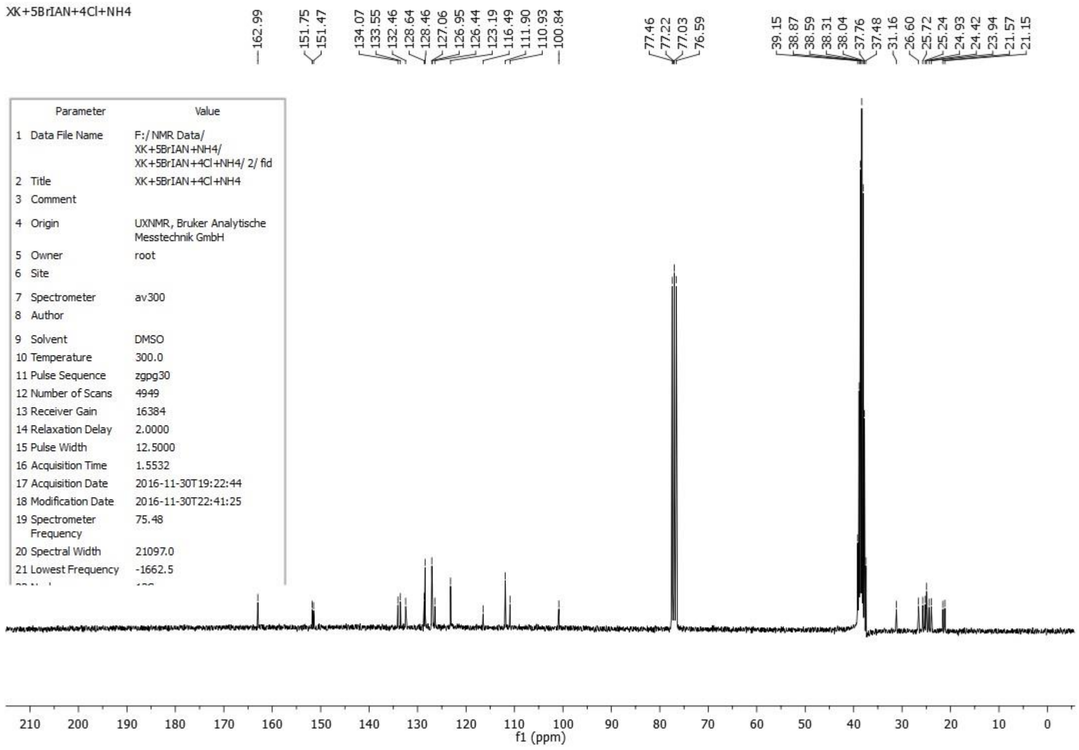

Figure 18: ^{1}H and ^{13}C NMR spectra of **14f**

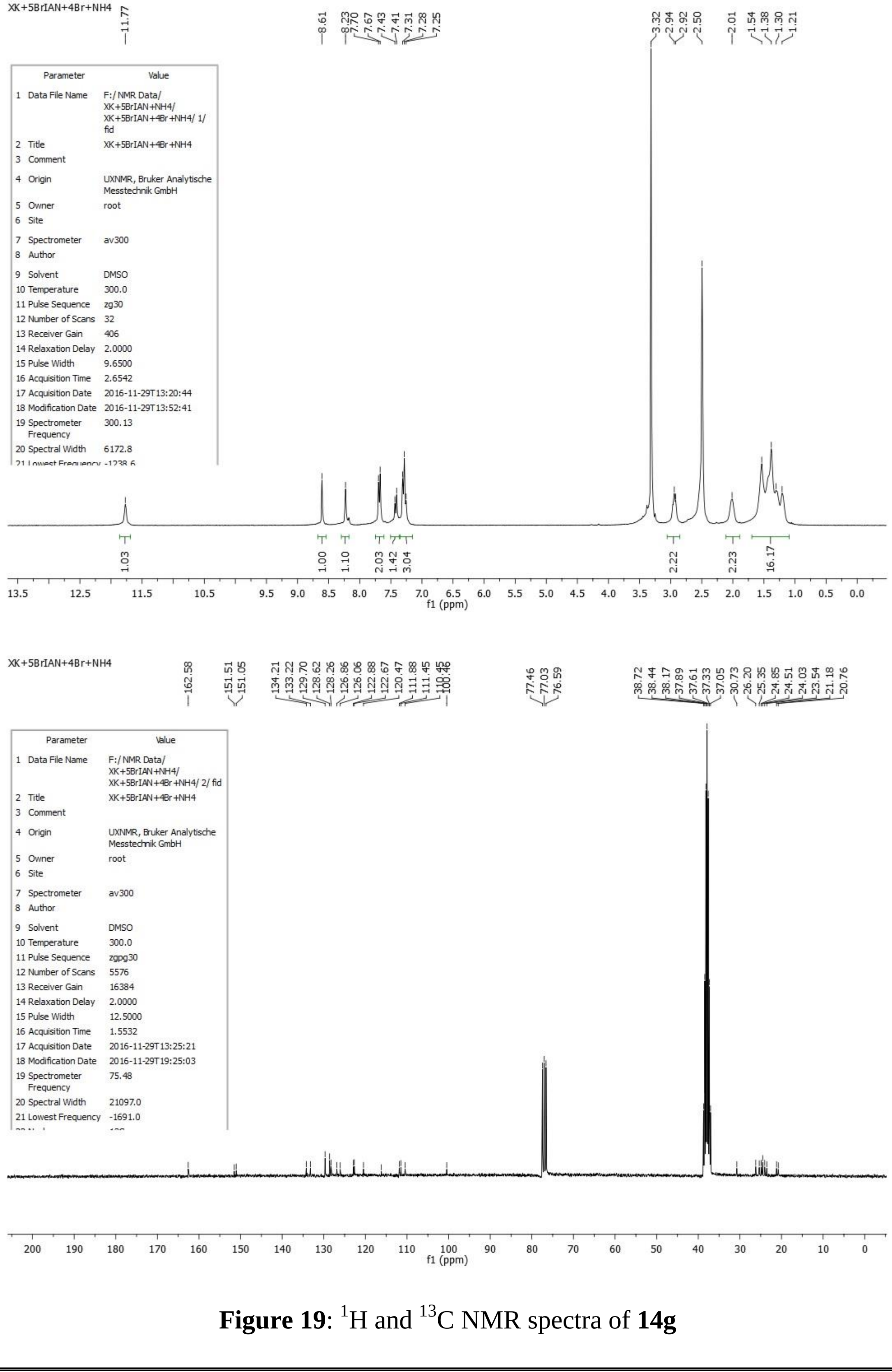

Figure 19: ^{1}H and ^{13}C NMR spectra of **14g**

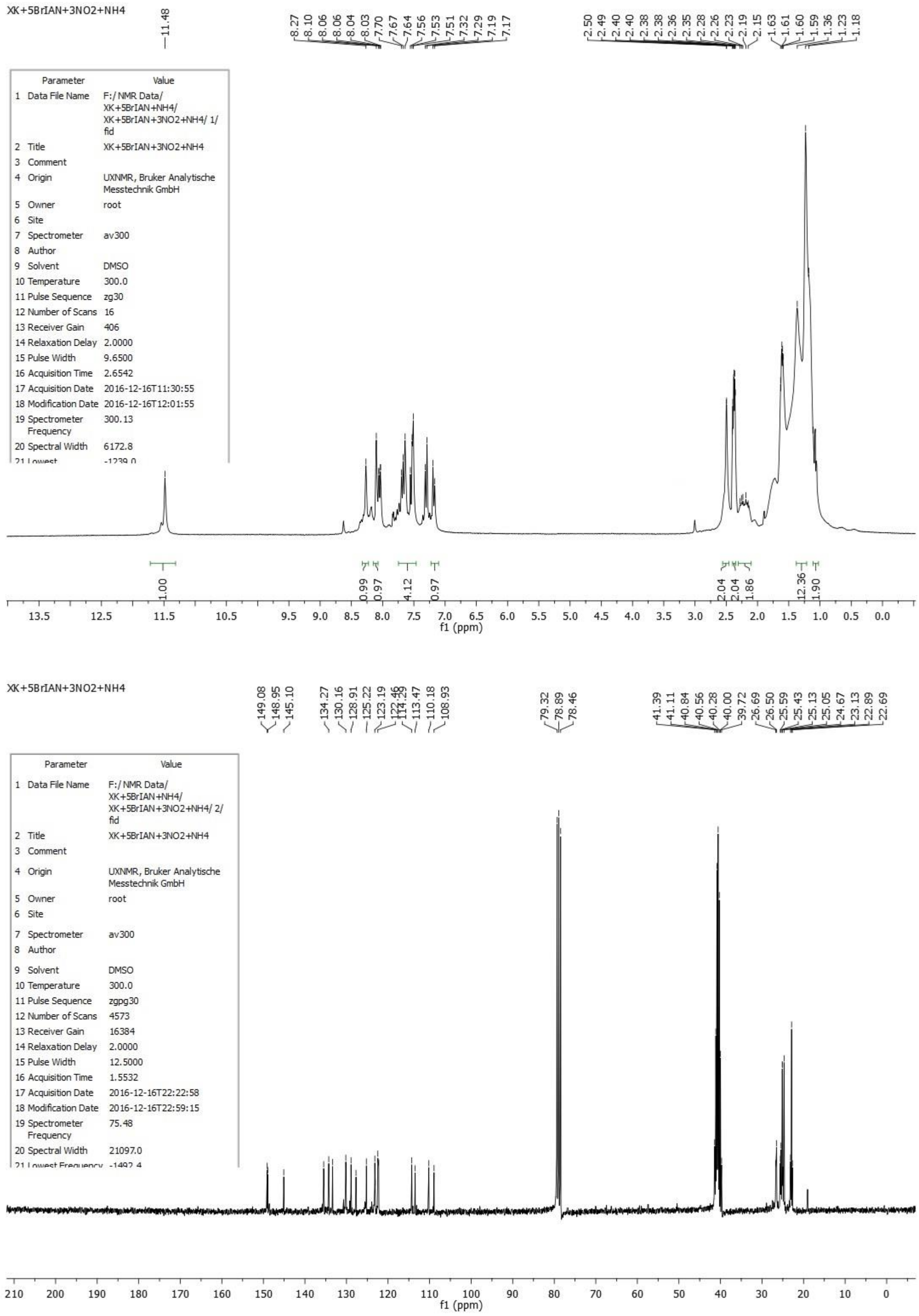

Figure 20: ^{1}H and ^{13}C NMR spectra of **14m**

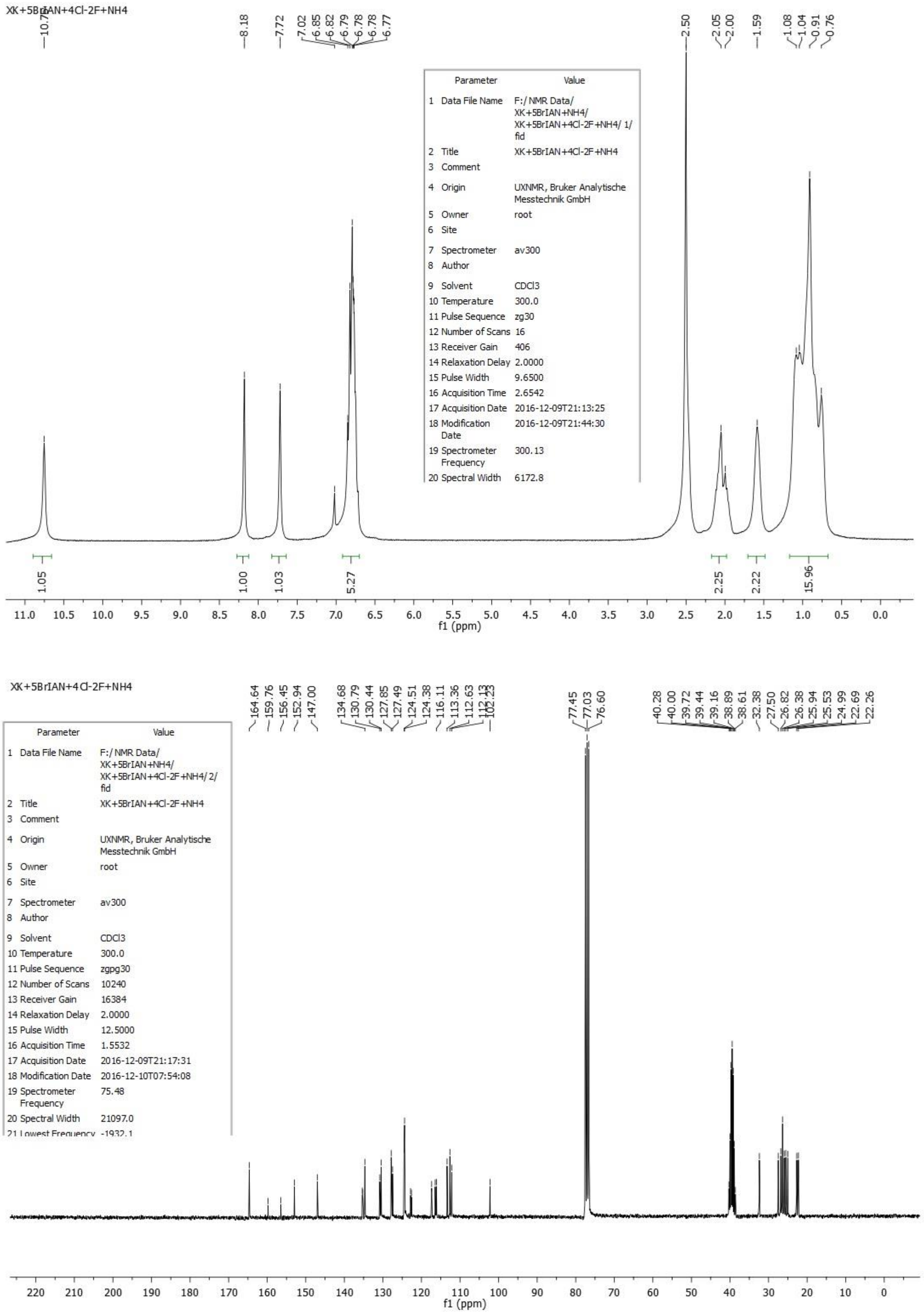

Figure 21: ^{1}H and ^{13}C NMR spectra of **14p**

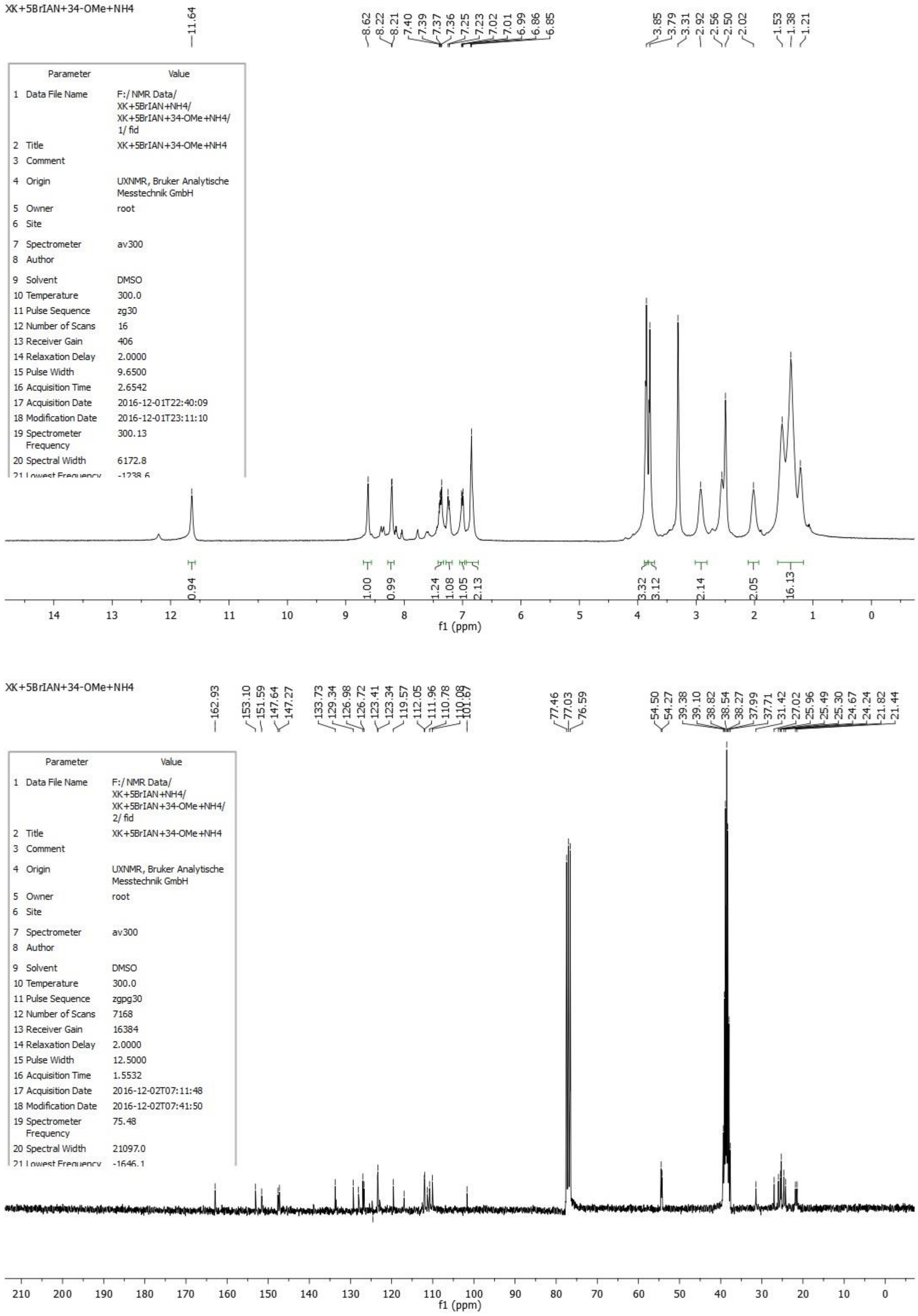

Figure 22: ^{1}H and ^{13}C NMR spectra of **14s**

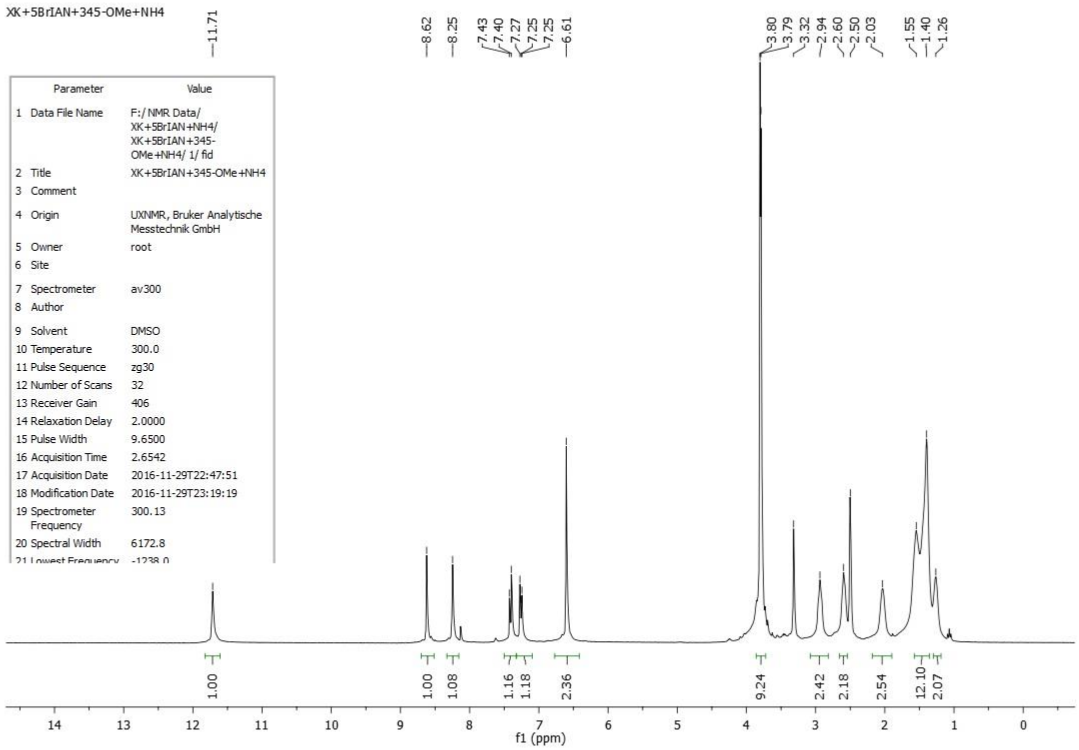

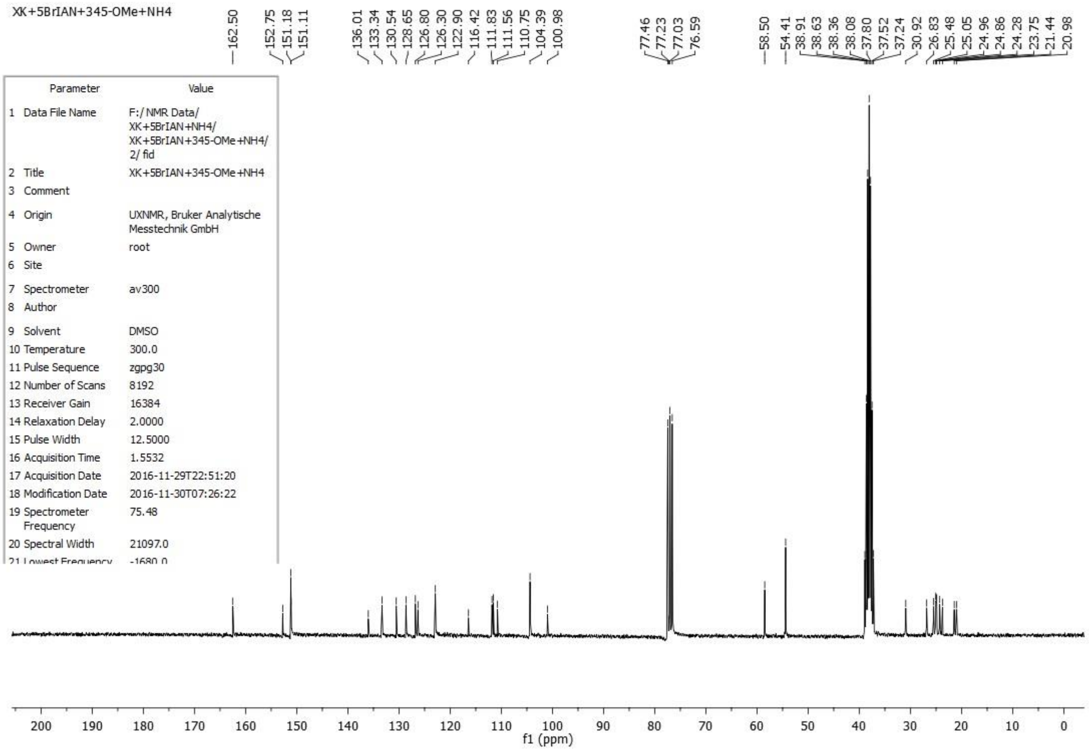

Figure 23: ^{1}H and ^{13}C NMR spectra of **14t**

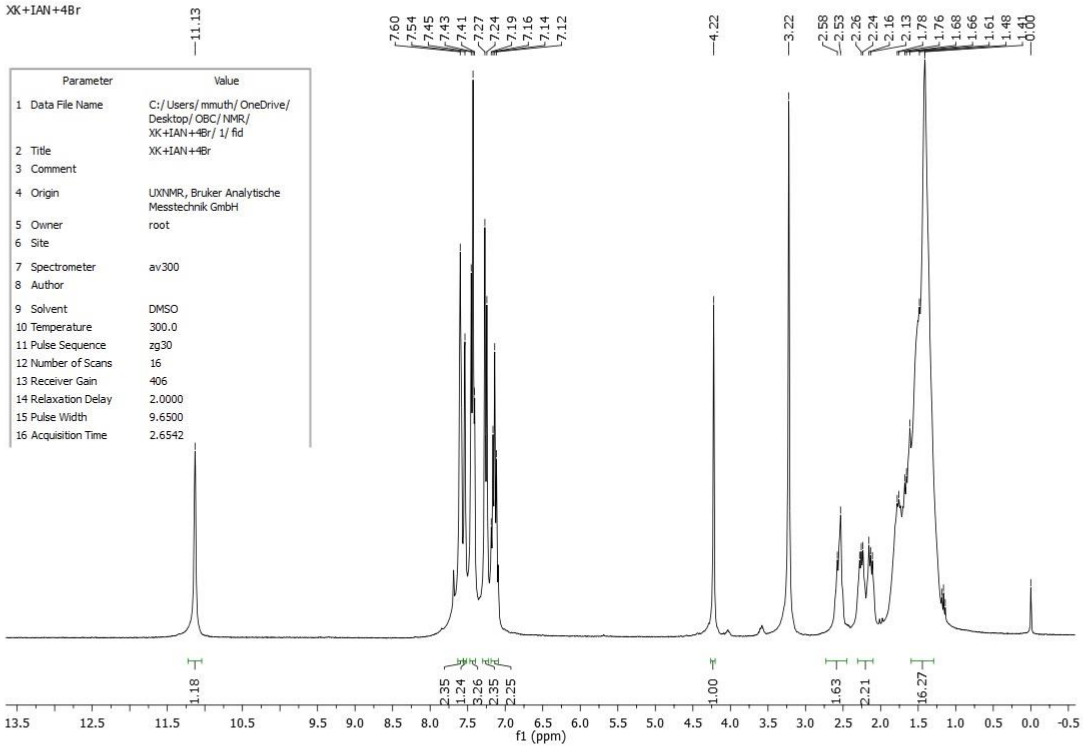

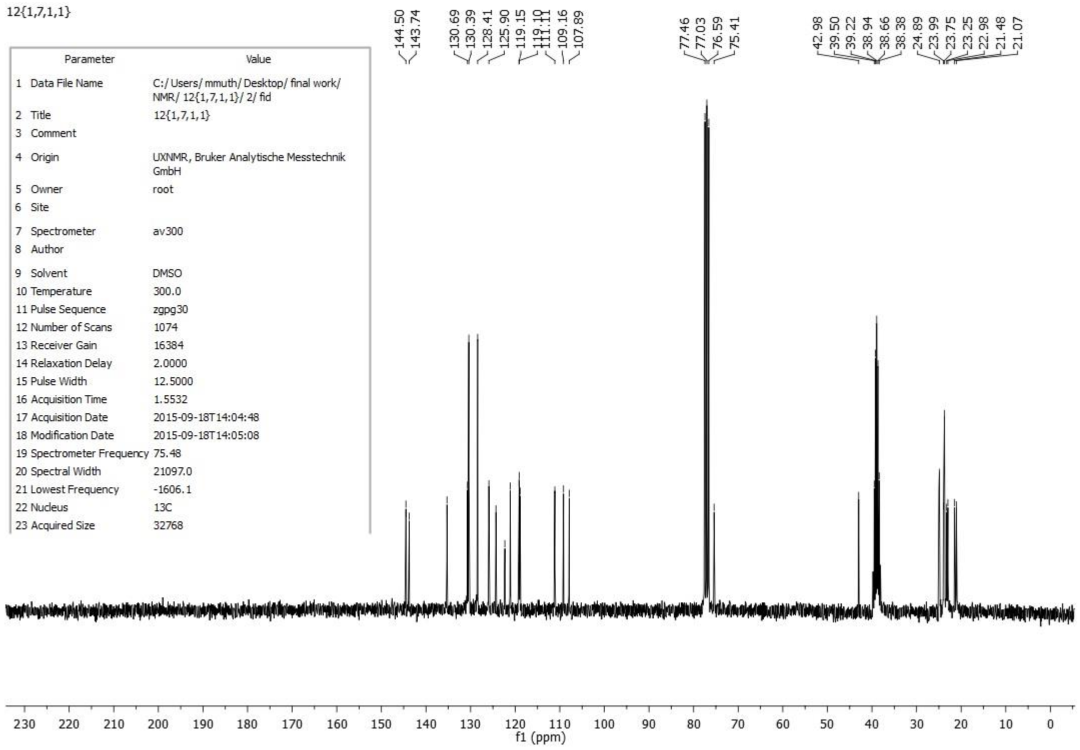

Figure 24: ^{1}H and ^{13}C NMR spectra of **12g**

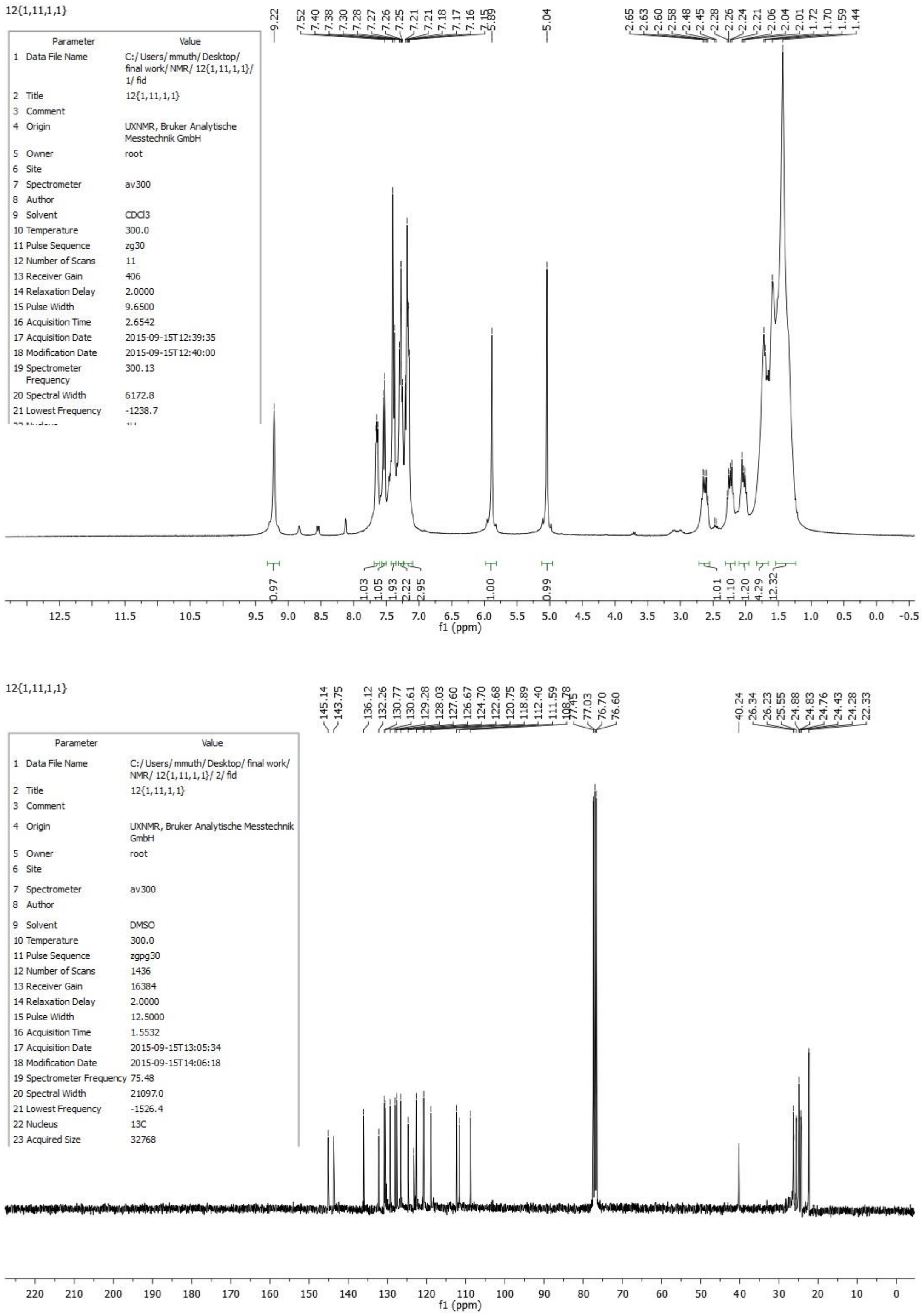

Figure 25: ^{1}H and ^{13}C NMR spectra of **12k**

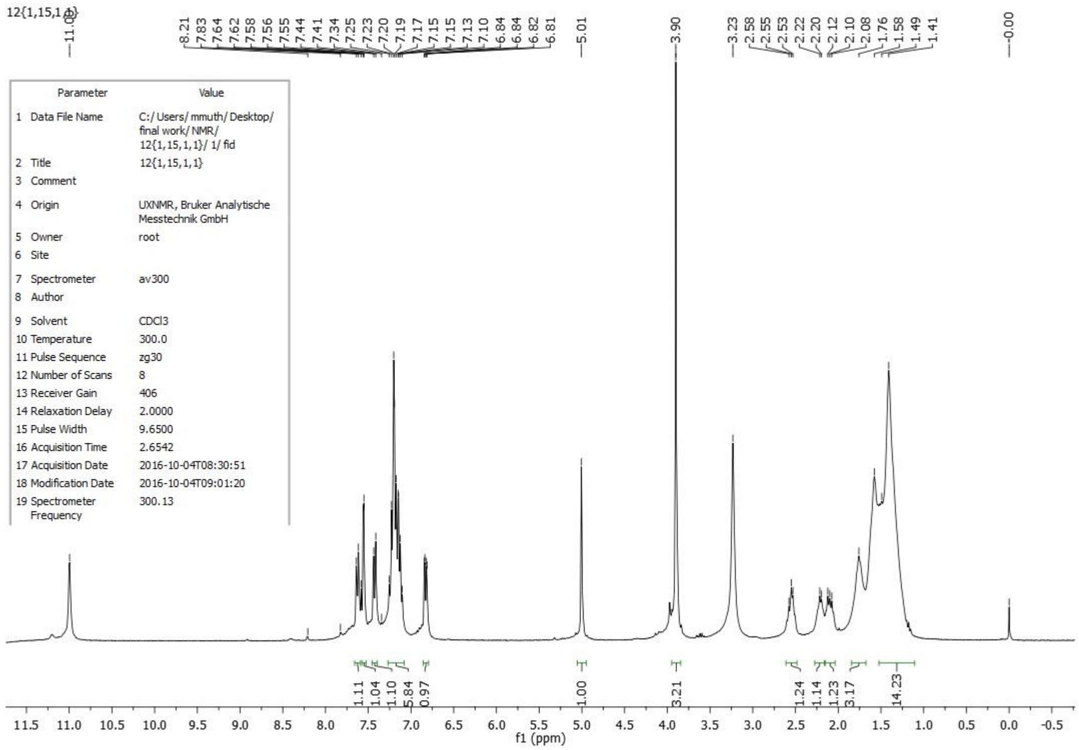

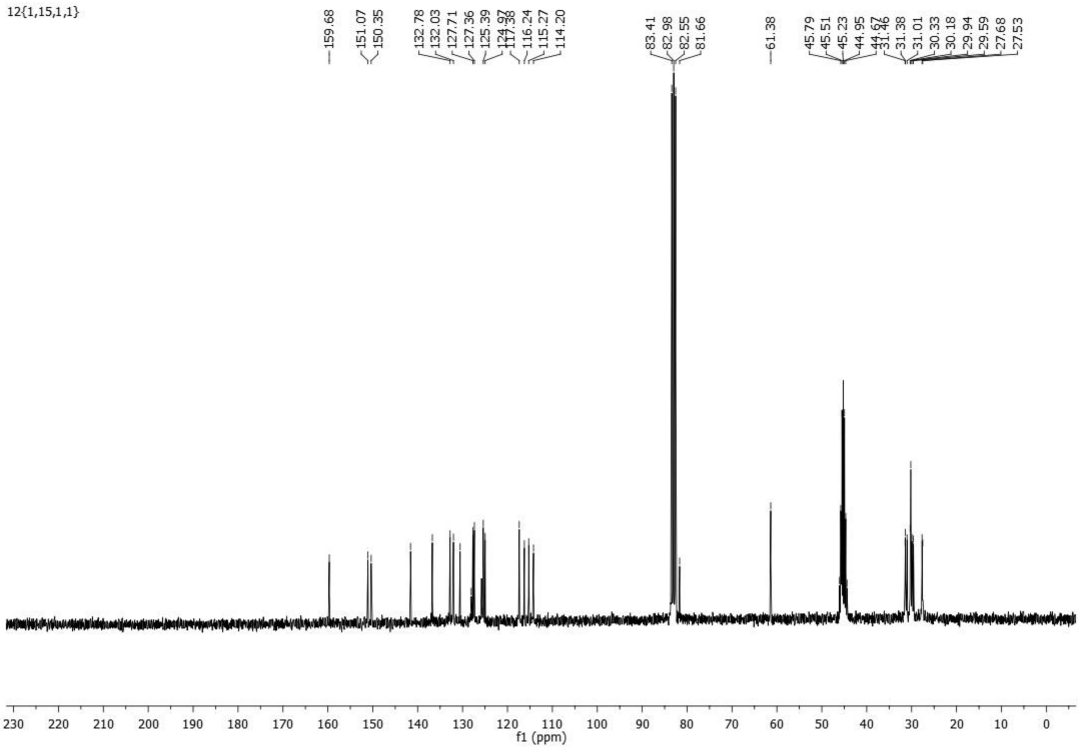

Figure 26: ^{1}H and ^{13}C NMR spectra of **12o**

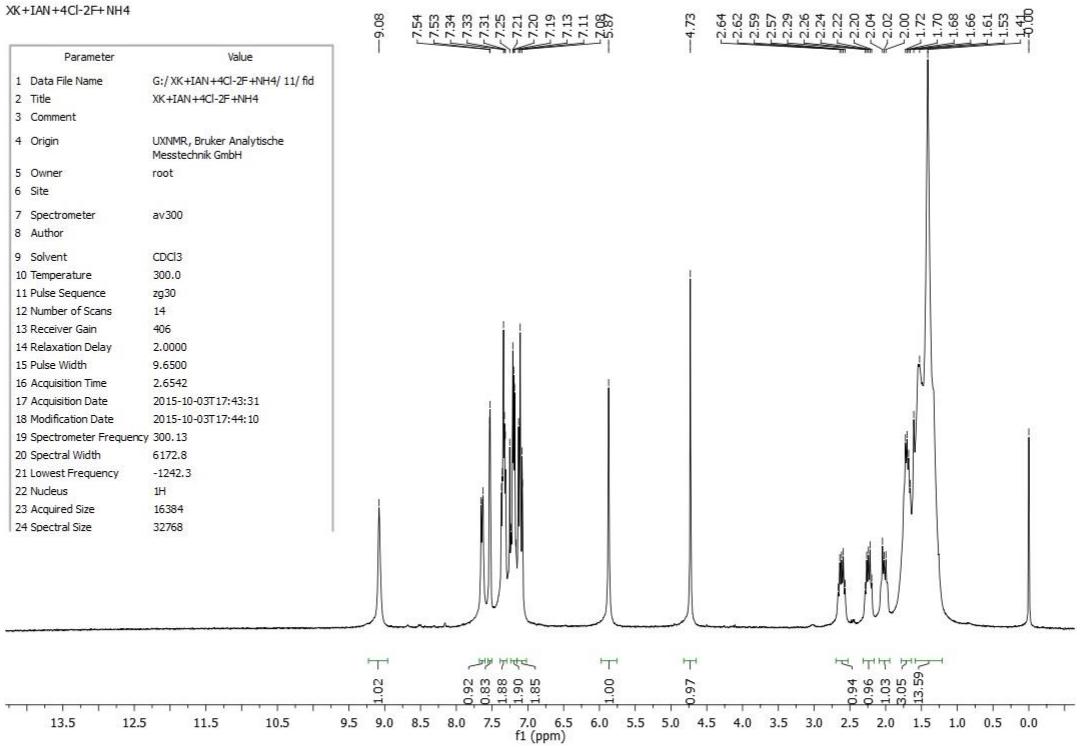

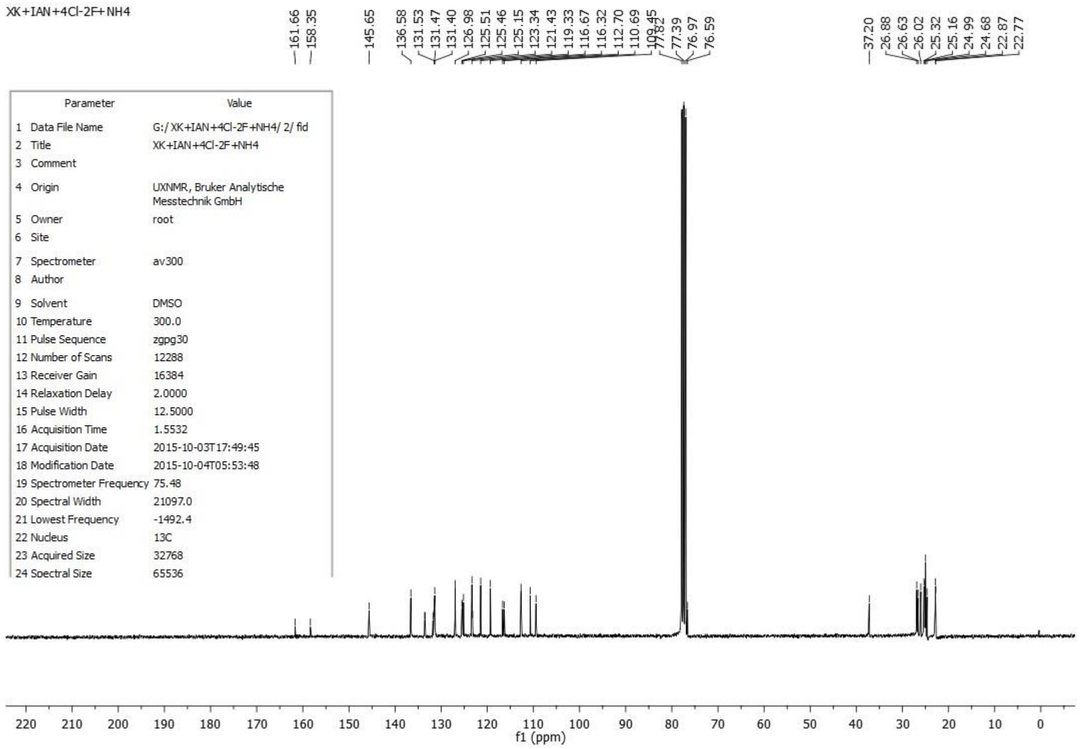

Figure 27: ^{1}H and ^{13}C NMR spectra of **12p**

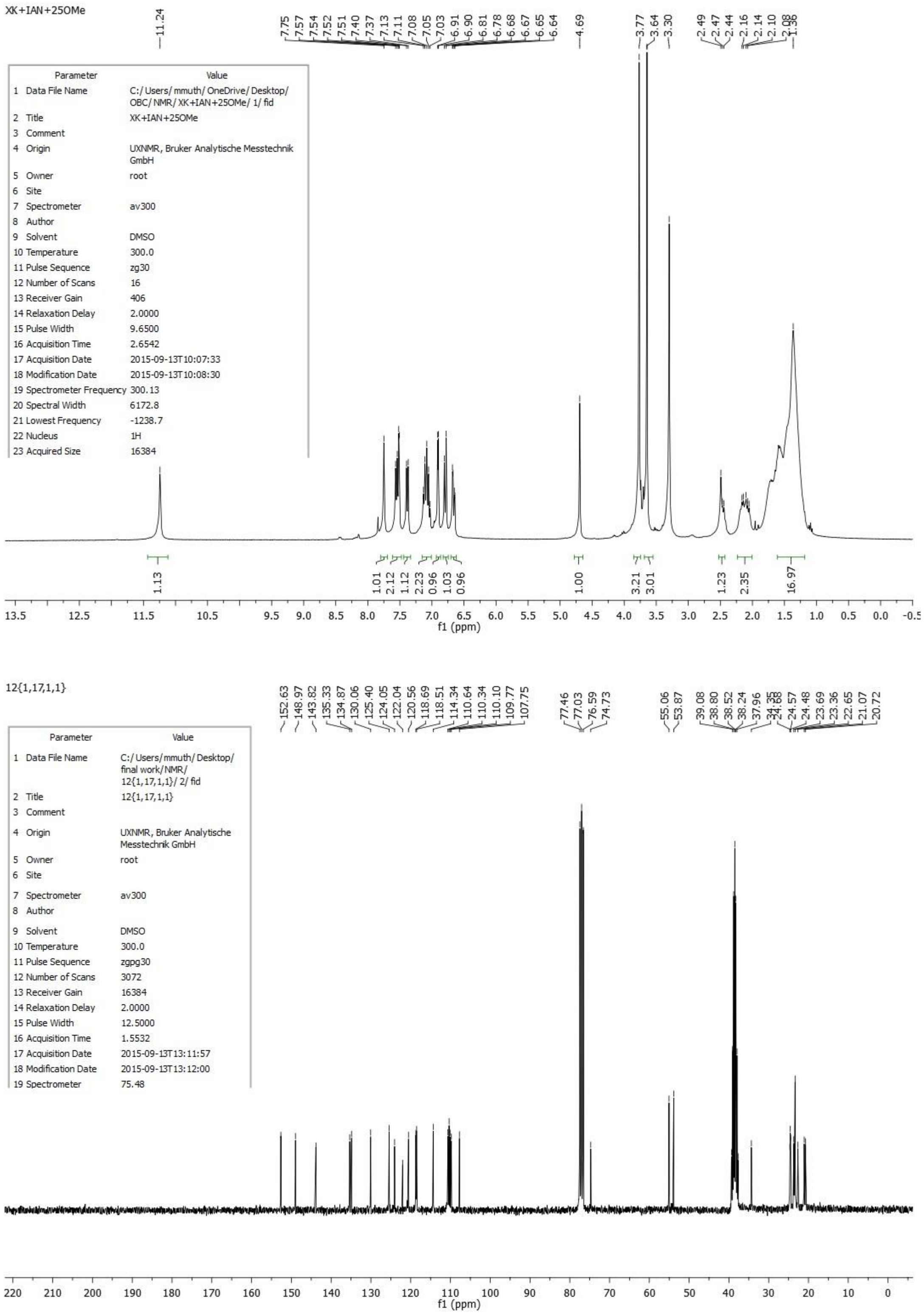

Figure 28: ^{1}H and ^{13}C NMR spectra of **12q**

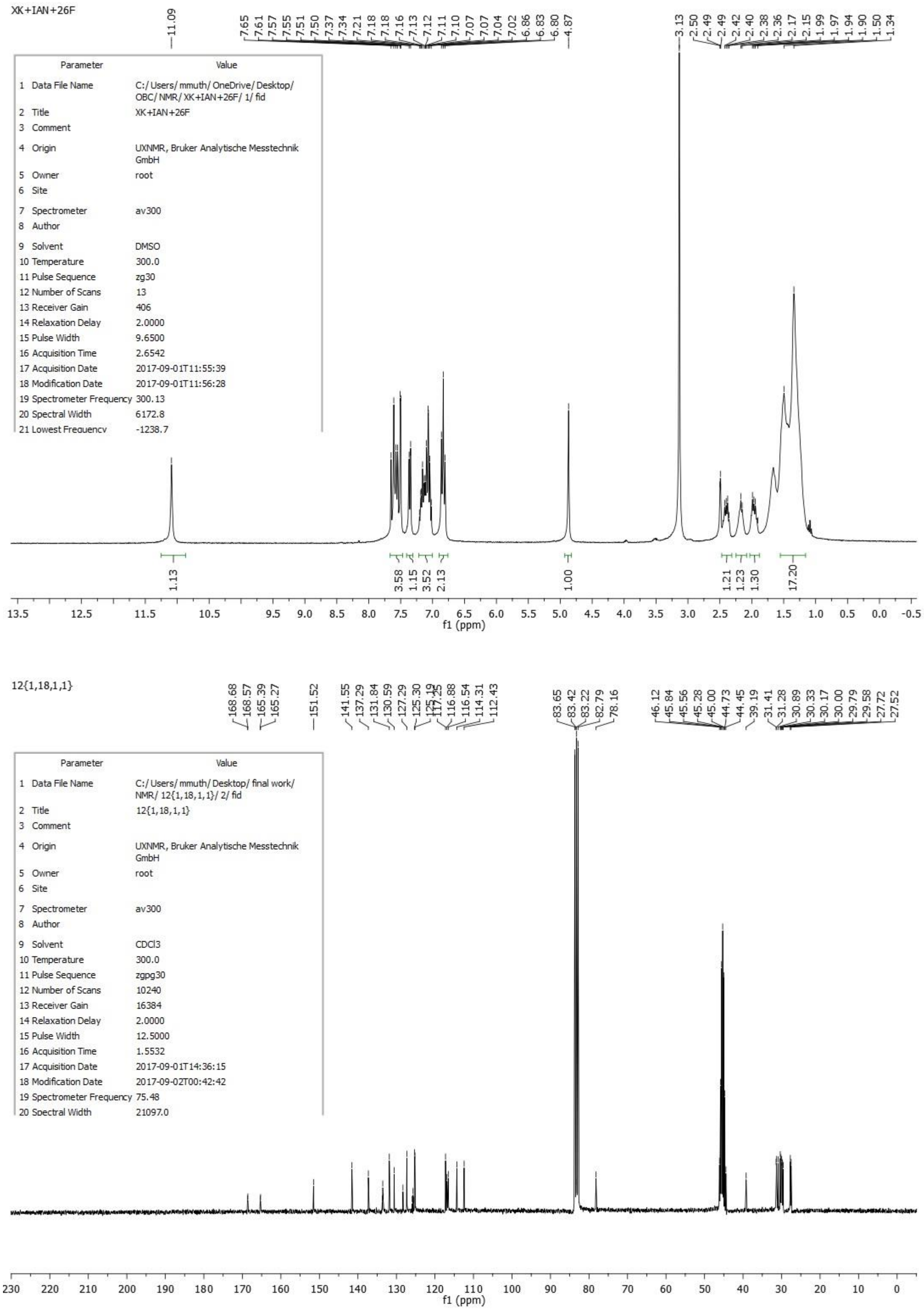

Figure 29: ^{1}H and ^{13}C NMR spectra of **12r**

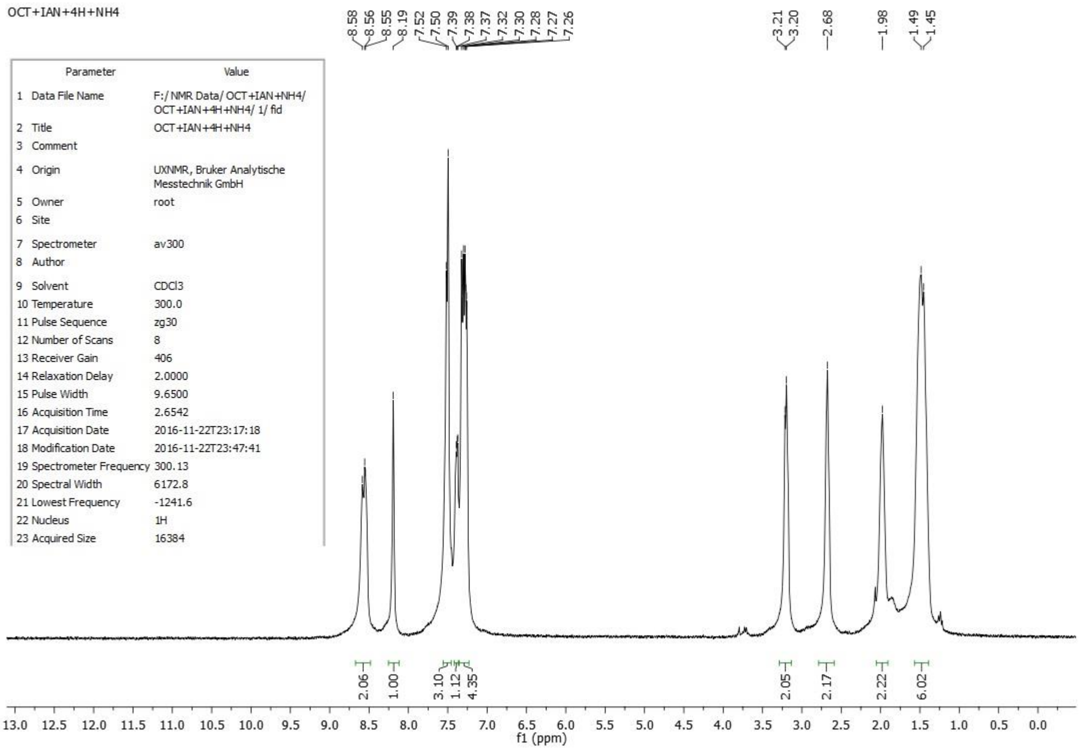

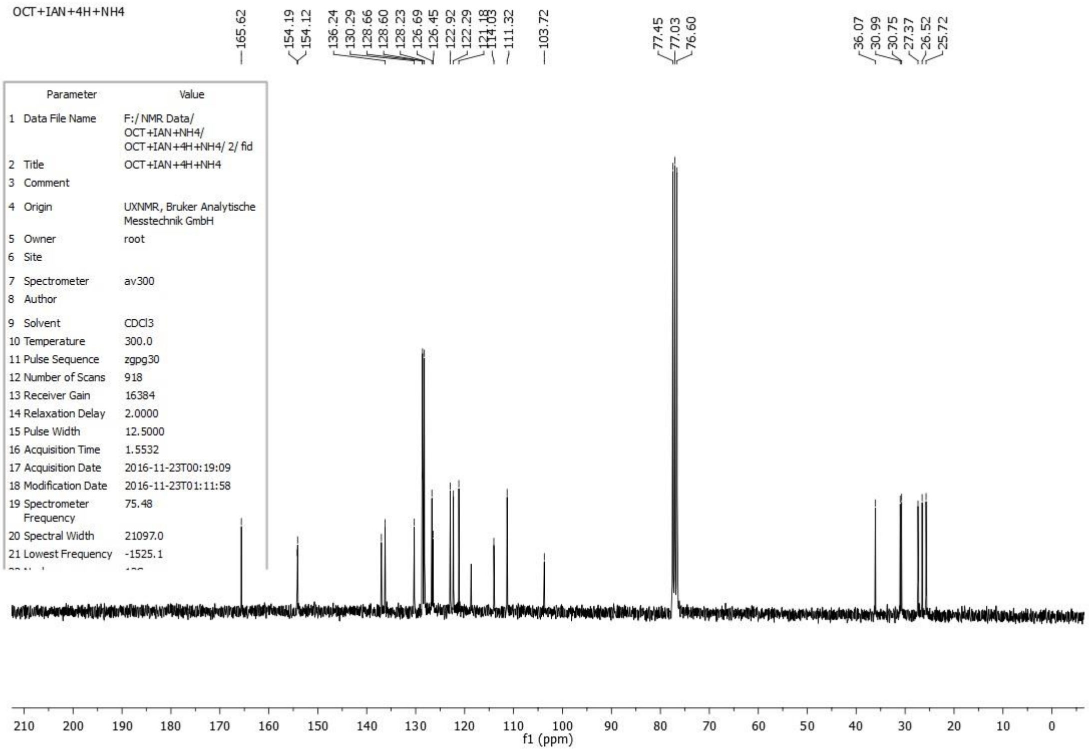

Figure 30: ^{1}H and ^{13}C NMR spectra of **15a**

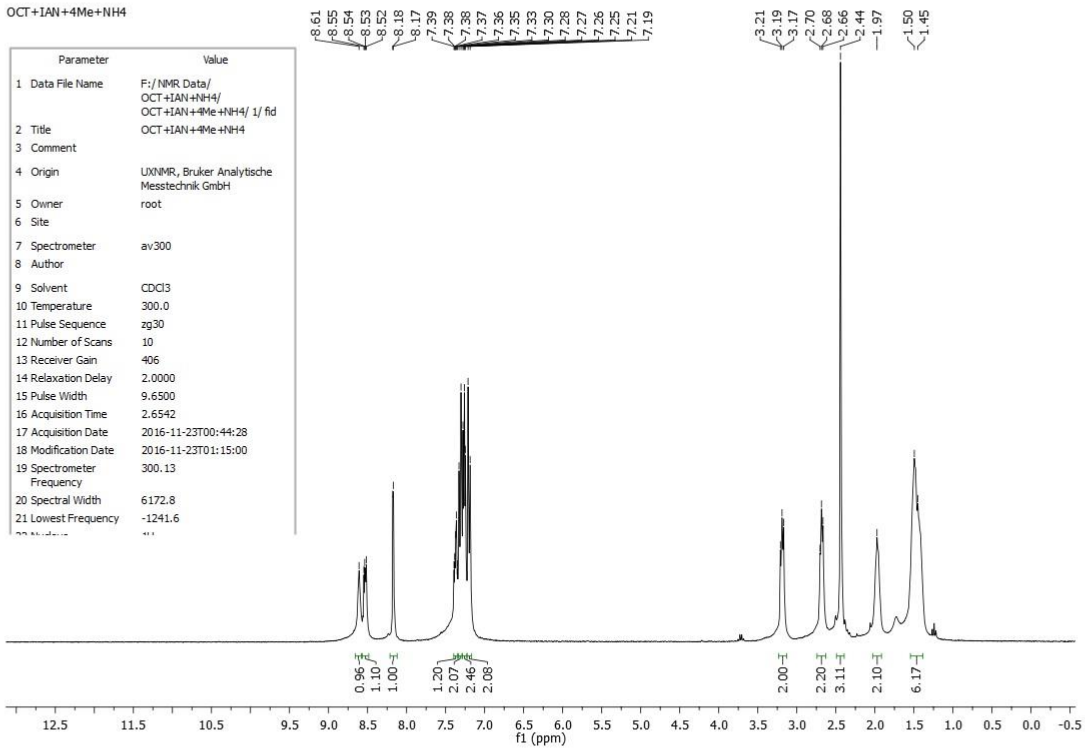

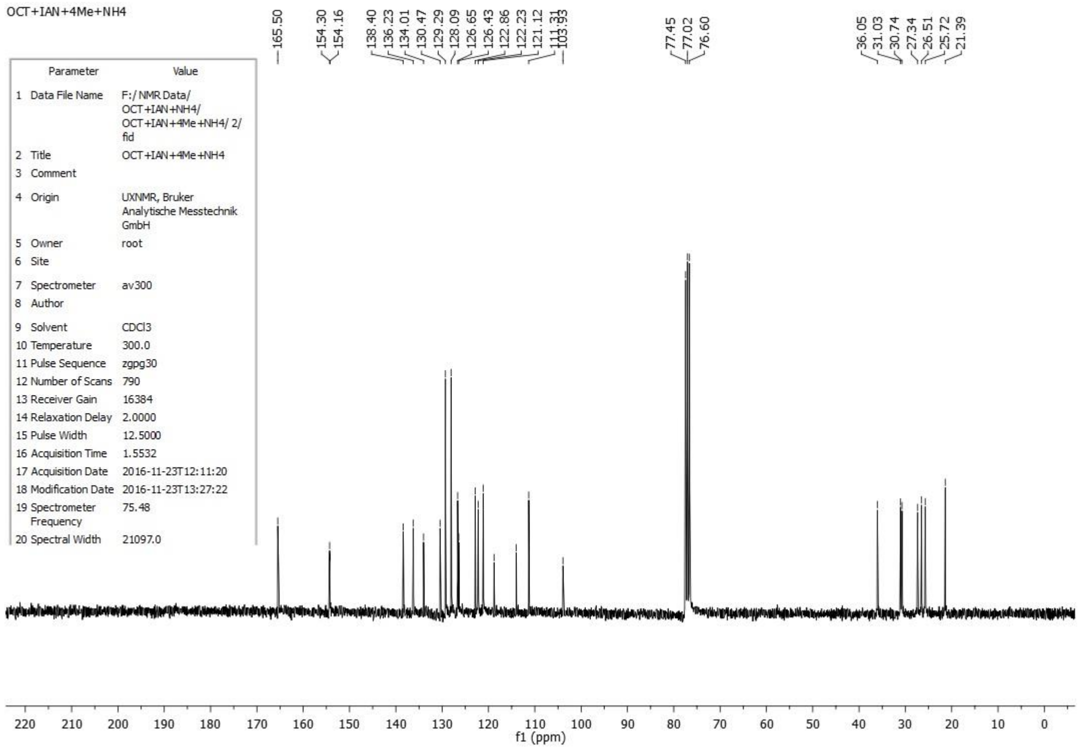

Figure 31: ^{1}H and ^{13}C NMR spectra of **15b**

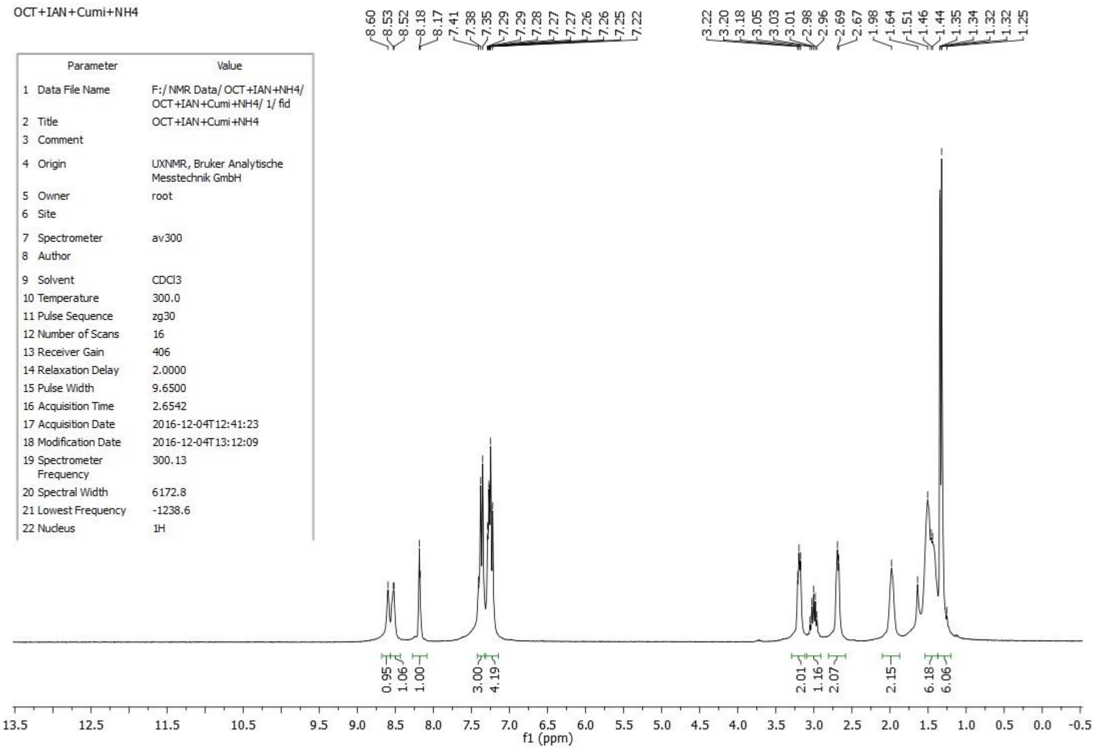

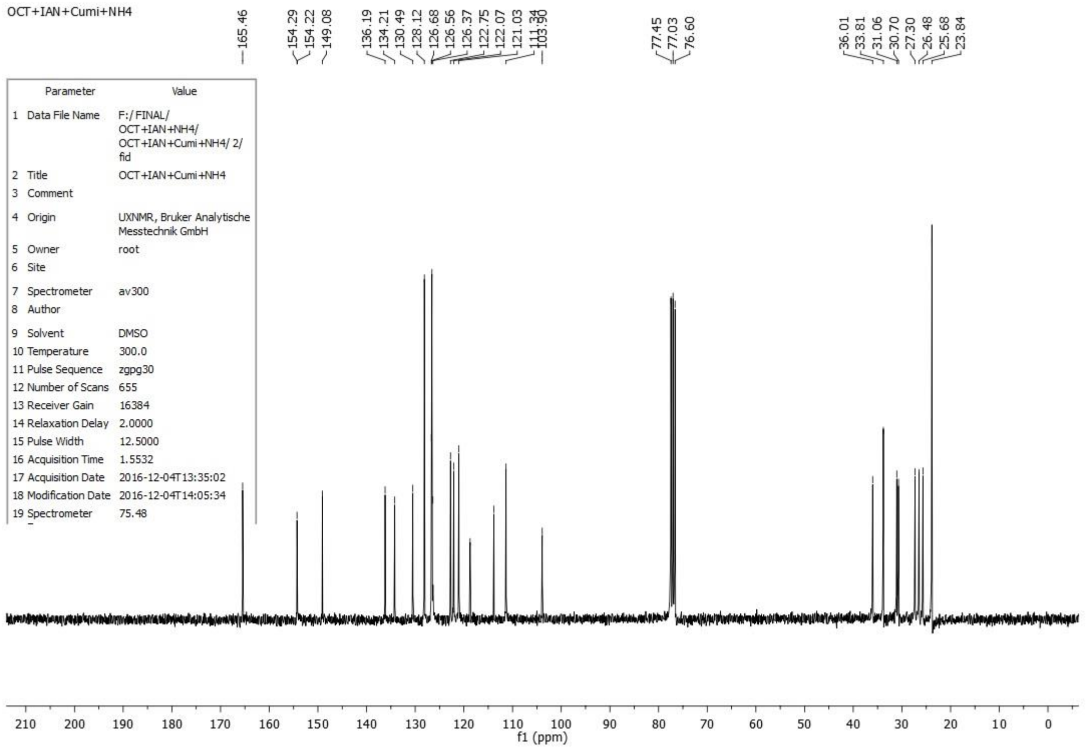

Figure 32: ^{1}H and ^{13}C NMR spectra of **15d**

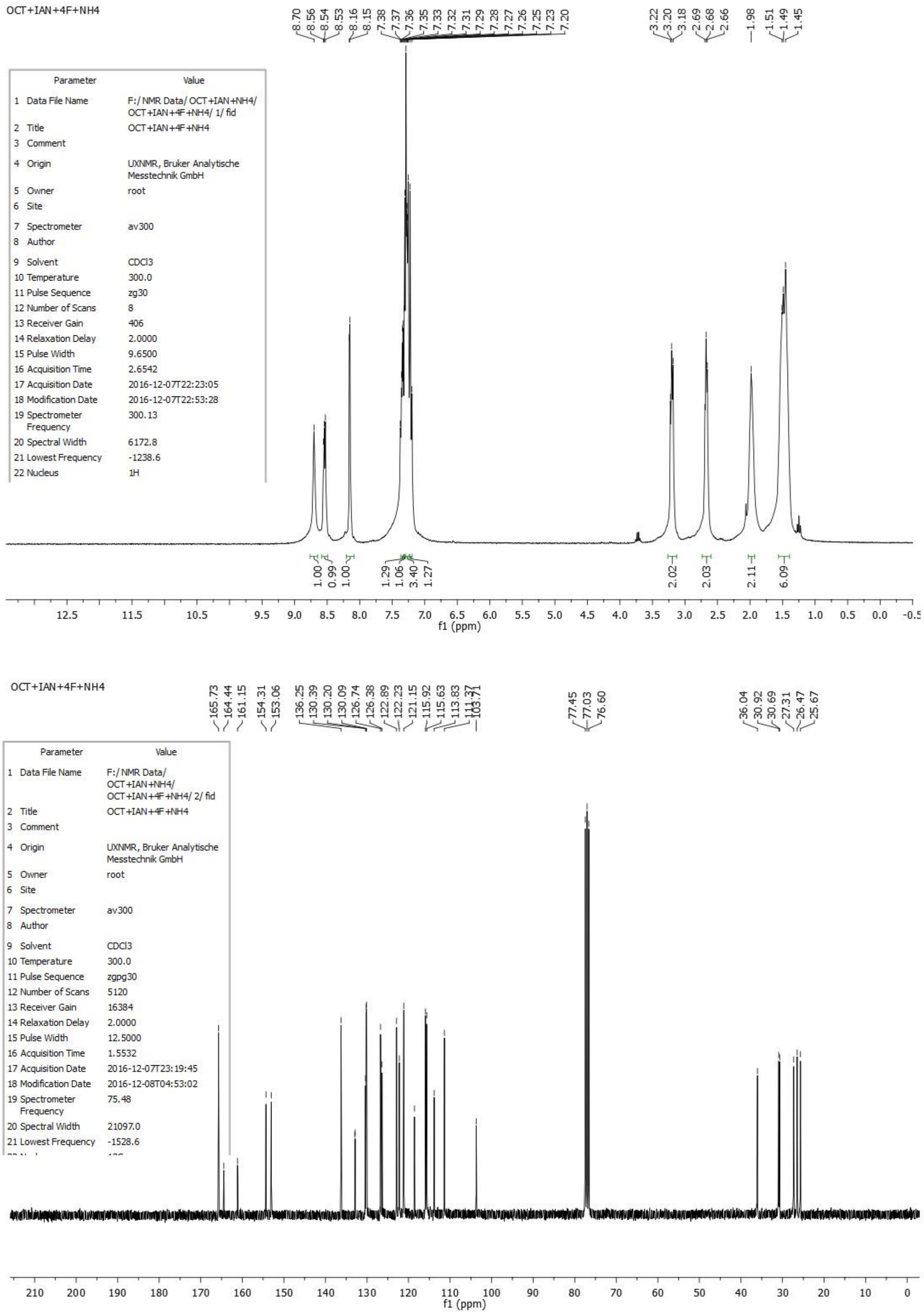

Figure 33: ^{1}H and ^{13}C NMR spectra of **15e**

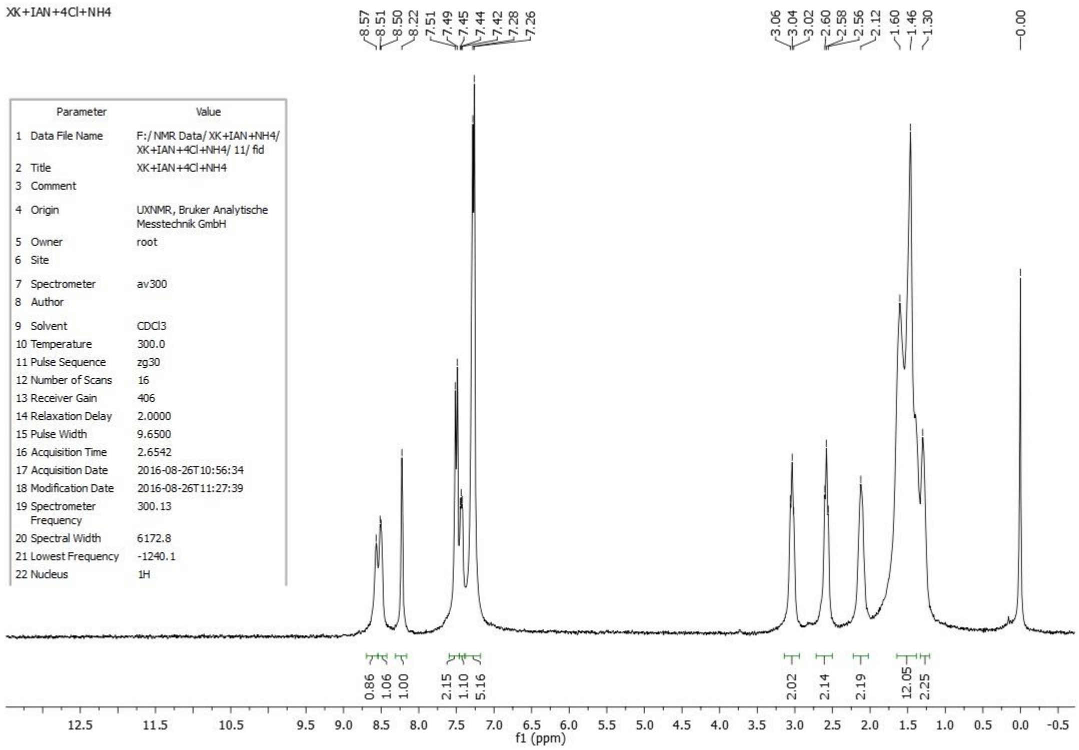

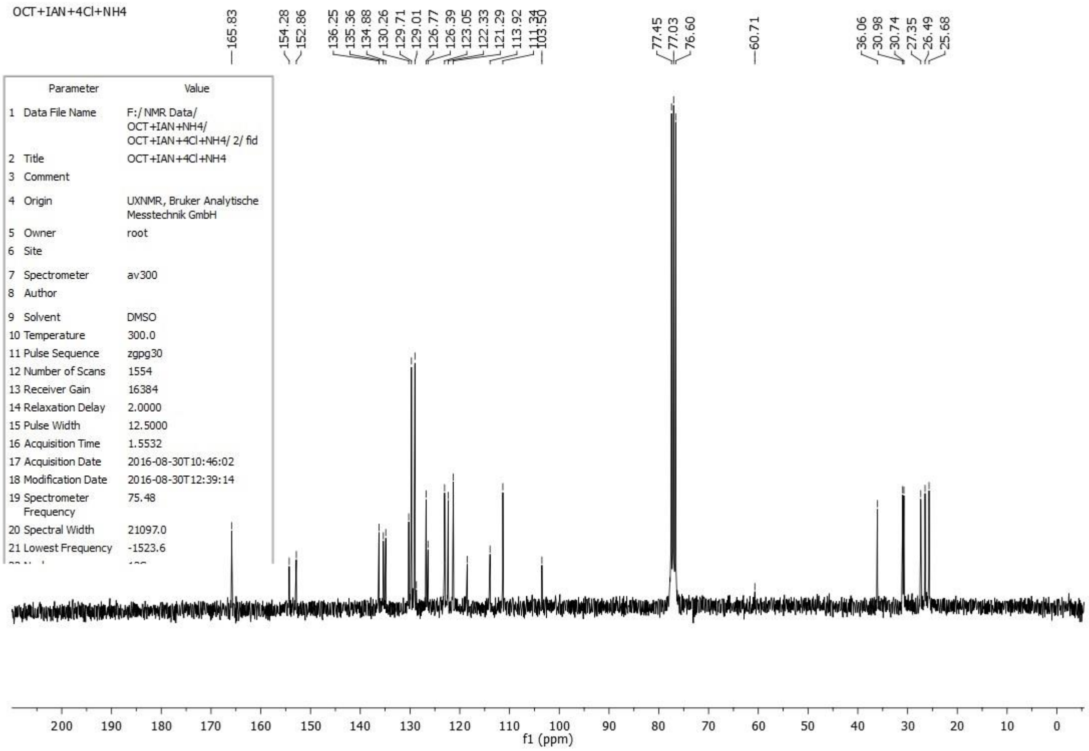

Figure 34: ^{1}H and ^{13}C NMR spectra of **15f**

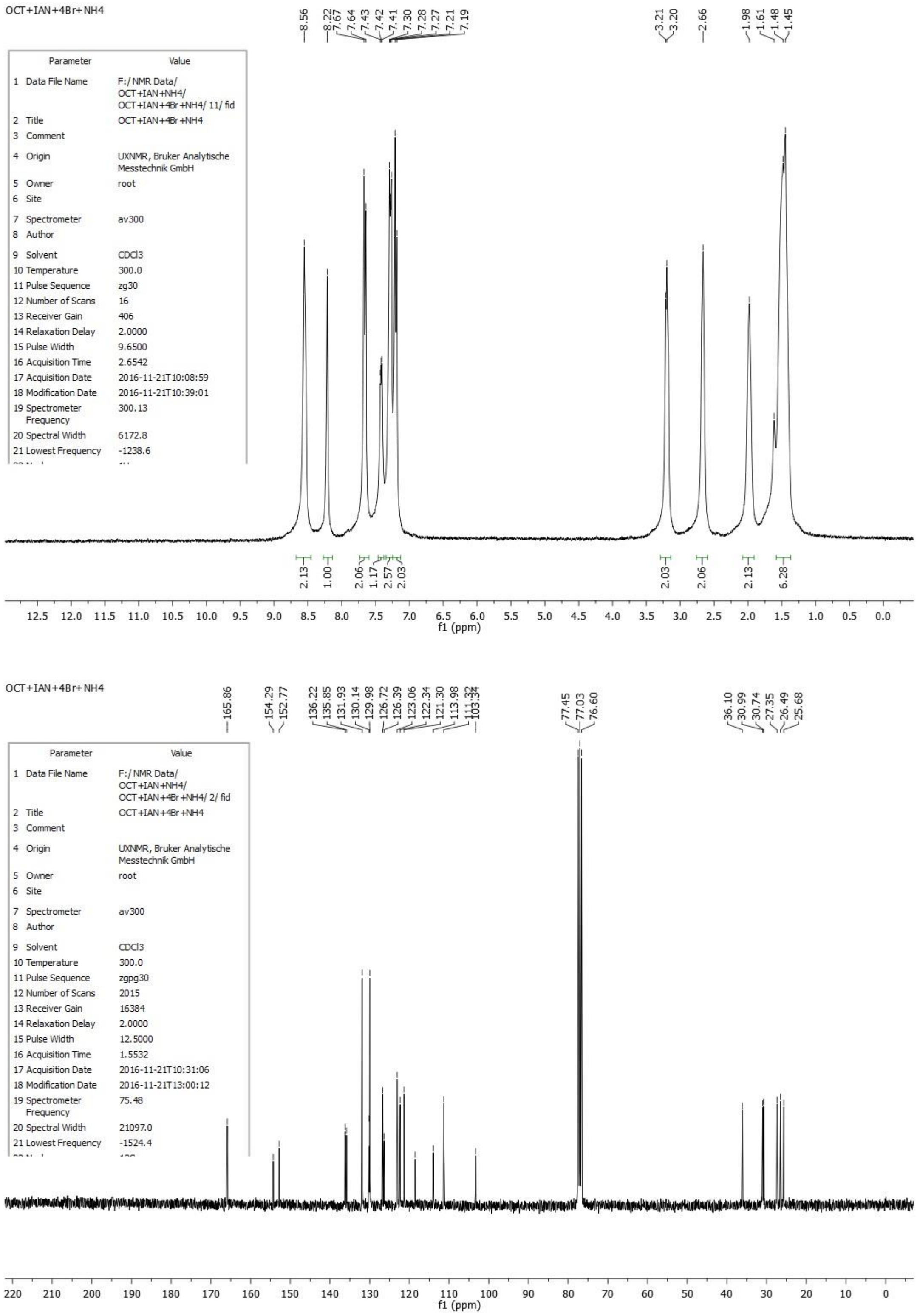

Figure 35: ^{1}H and ^{13}C NMR spectra of **15g**

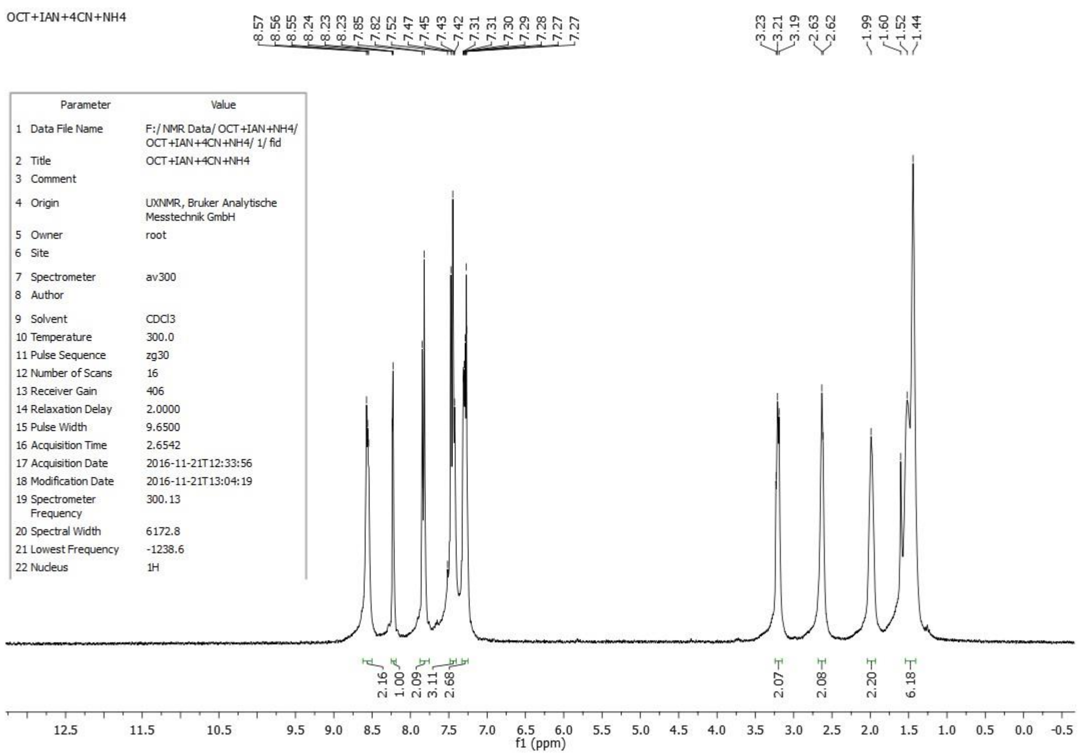

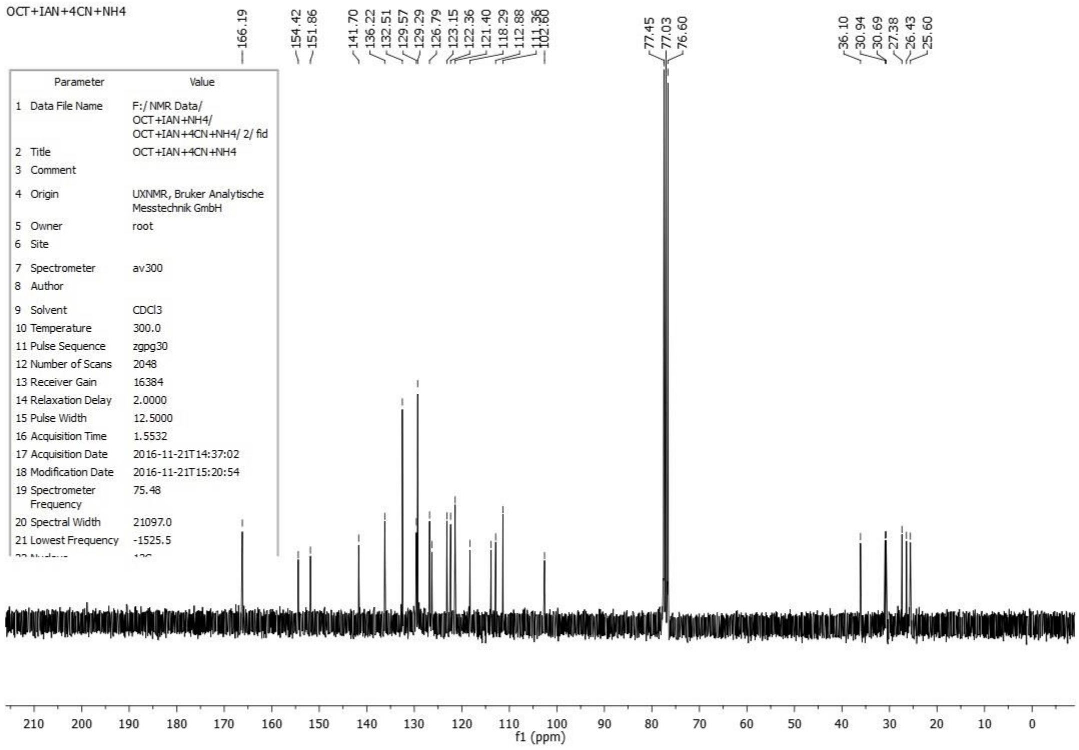

Figure 36: ^{1}H and ^{13}C NMR spectra of **15h**

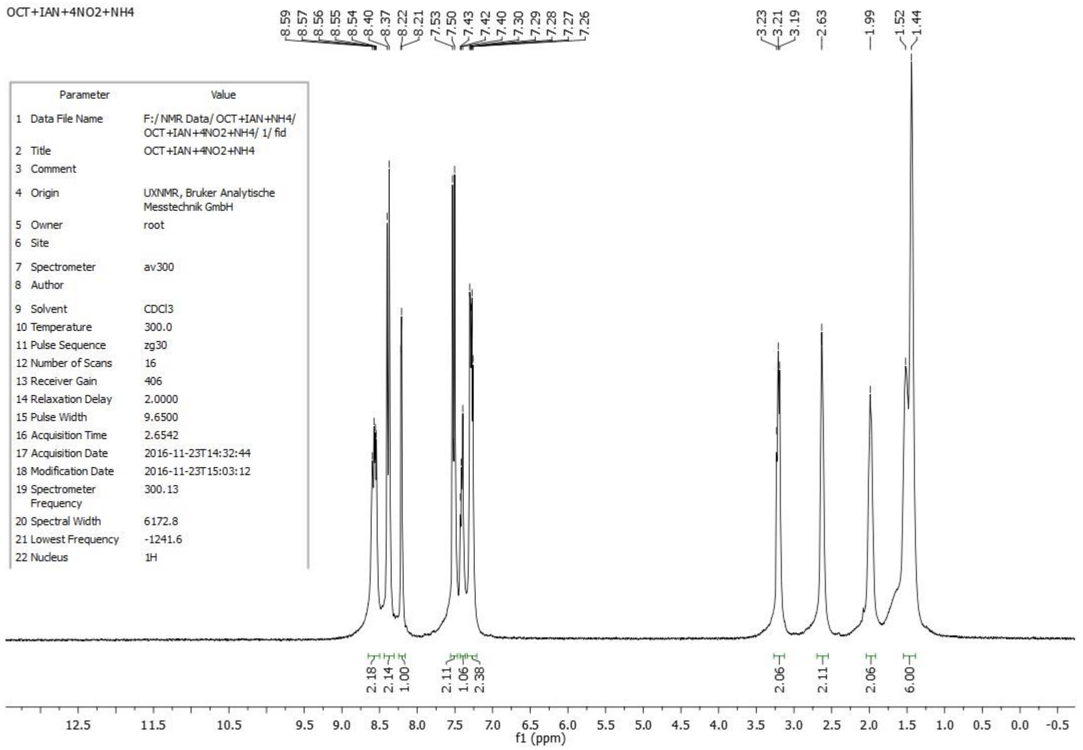

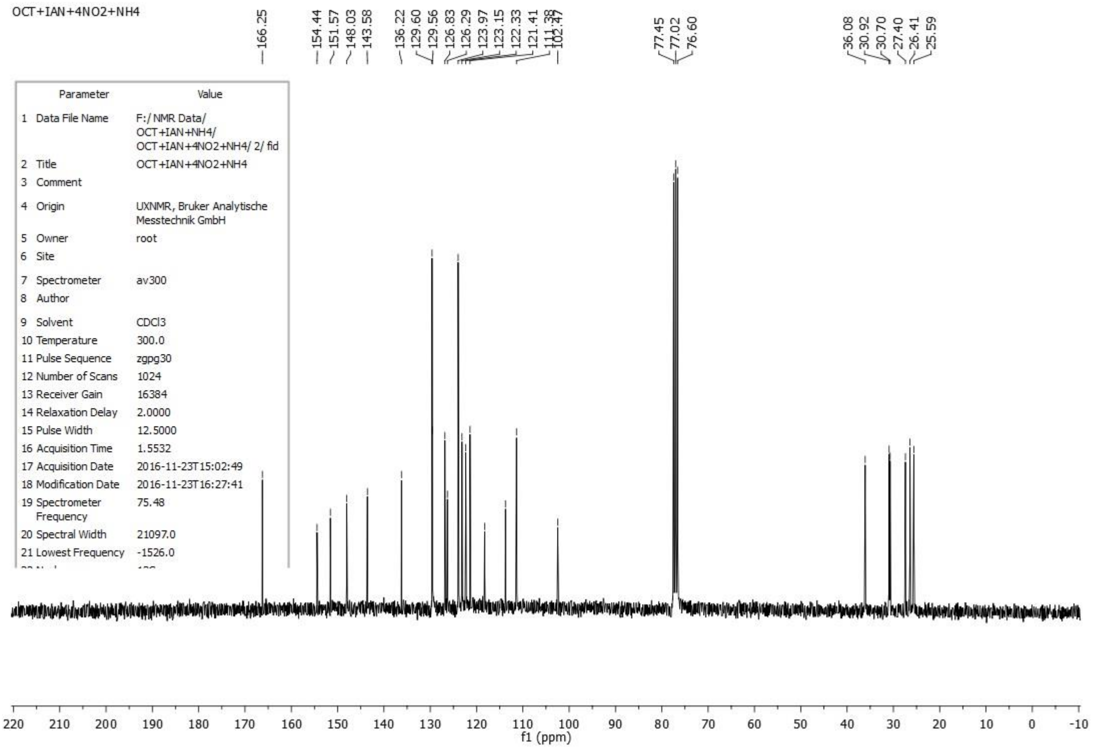

Figure 37: ^{1}H and ^{13}C NMR spectra of **15i**

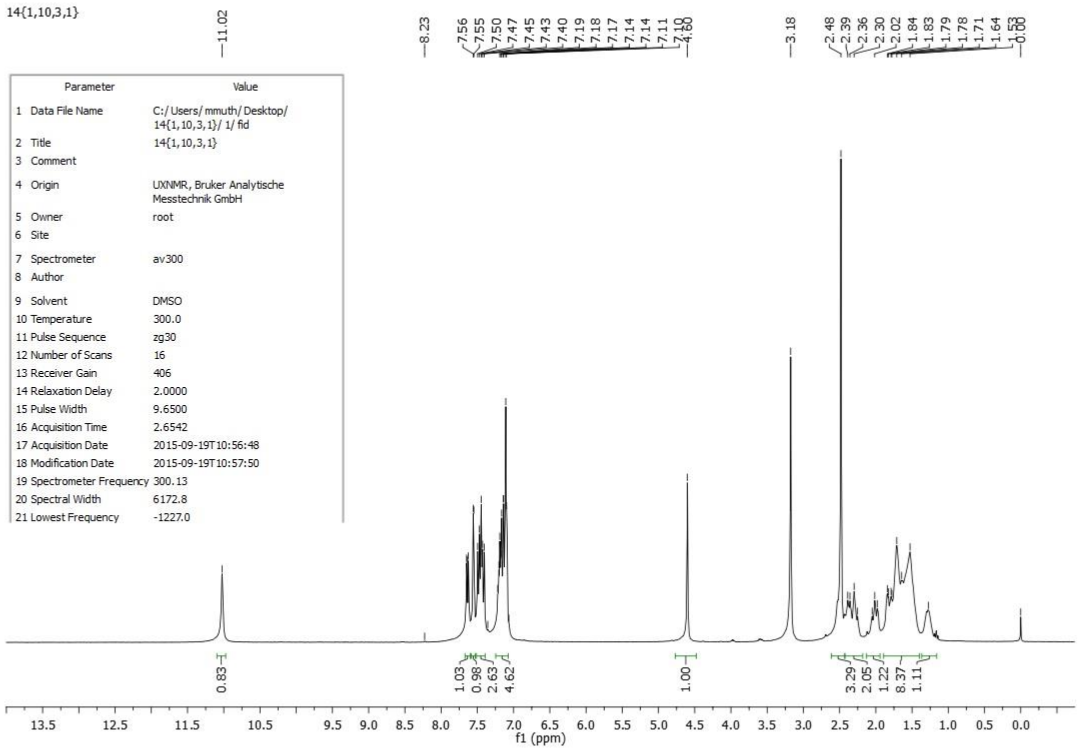

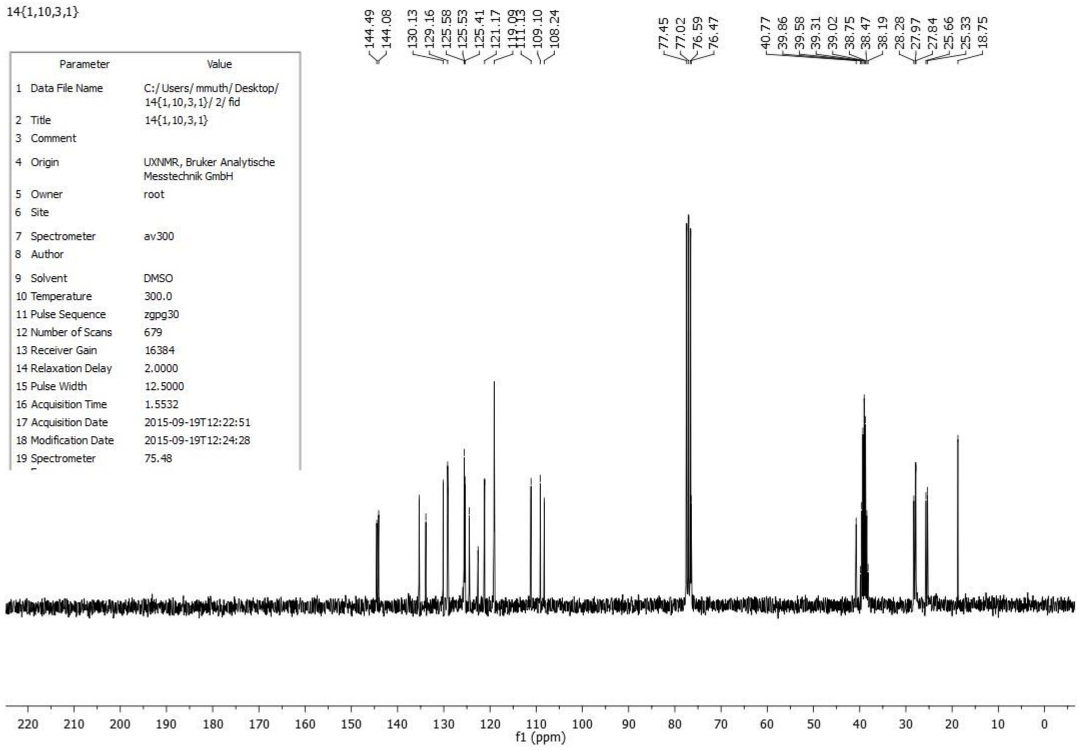

Figure 38: ^{1}H and ^{13}C NMR spectra of **15j**

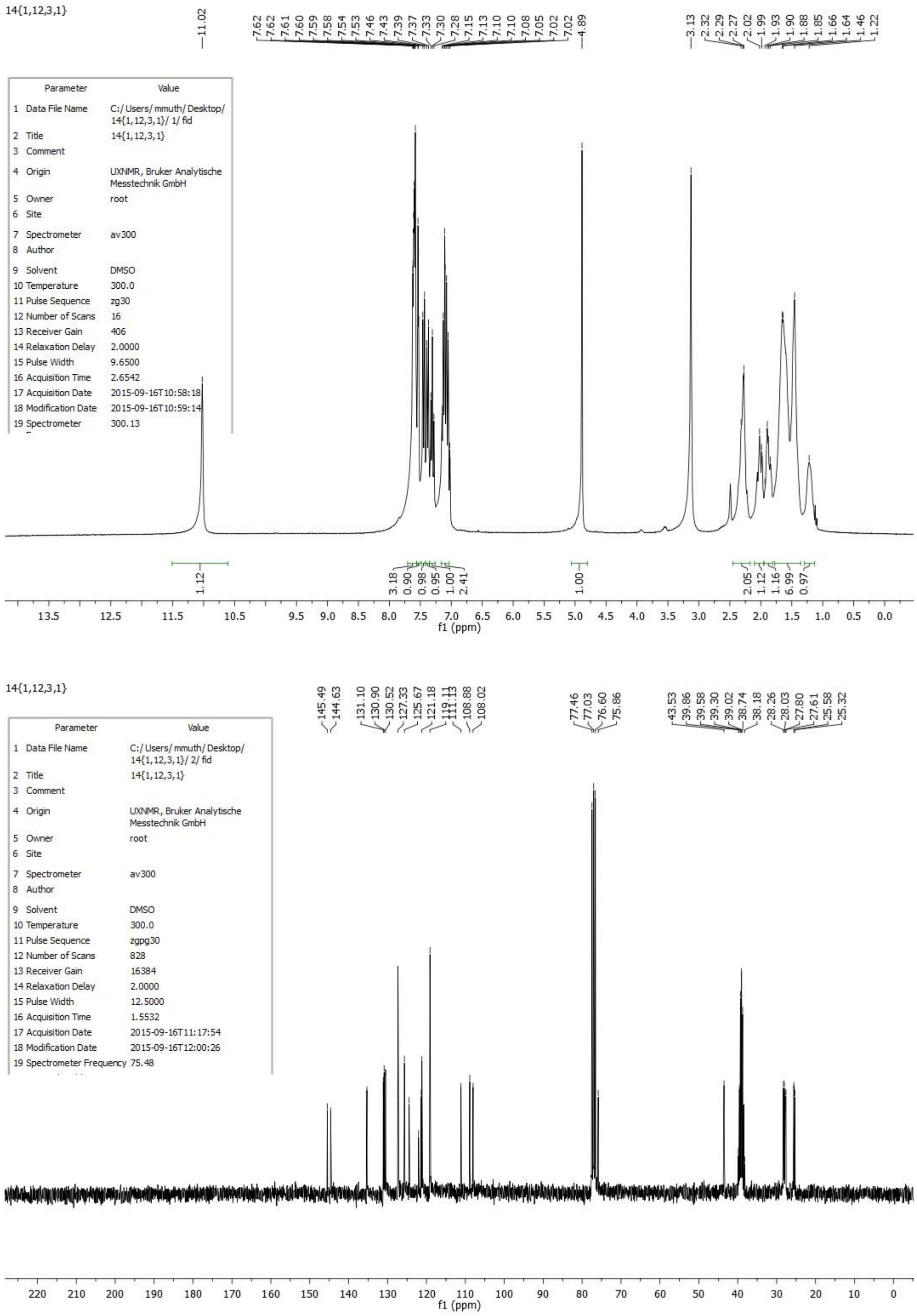

Figure 39: ^{1}H and ^{13}C NMR spectra of **15l**

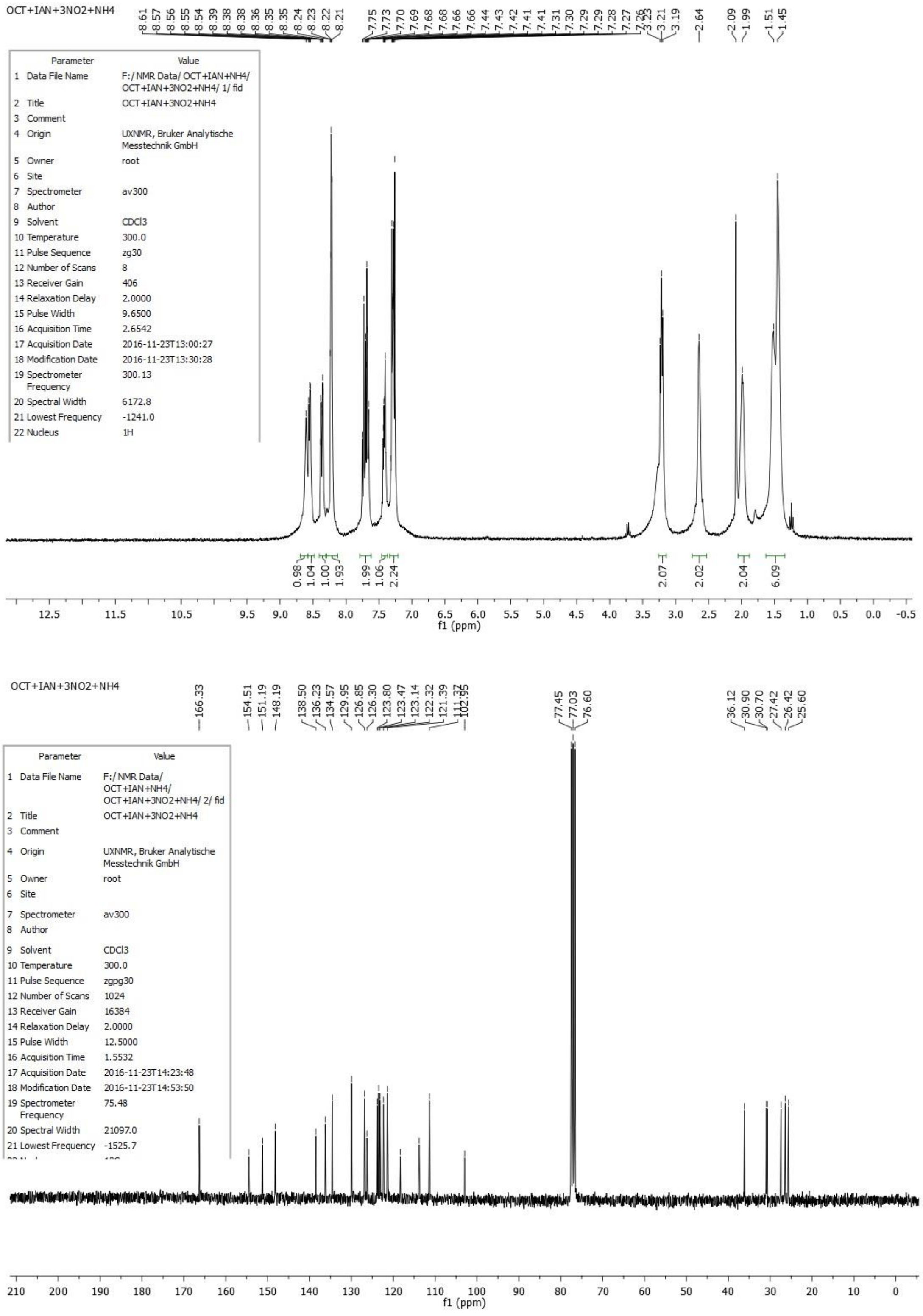

Figure 40: ^{1}H and ^{13}C NMR spectra of **15m**

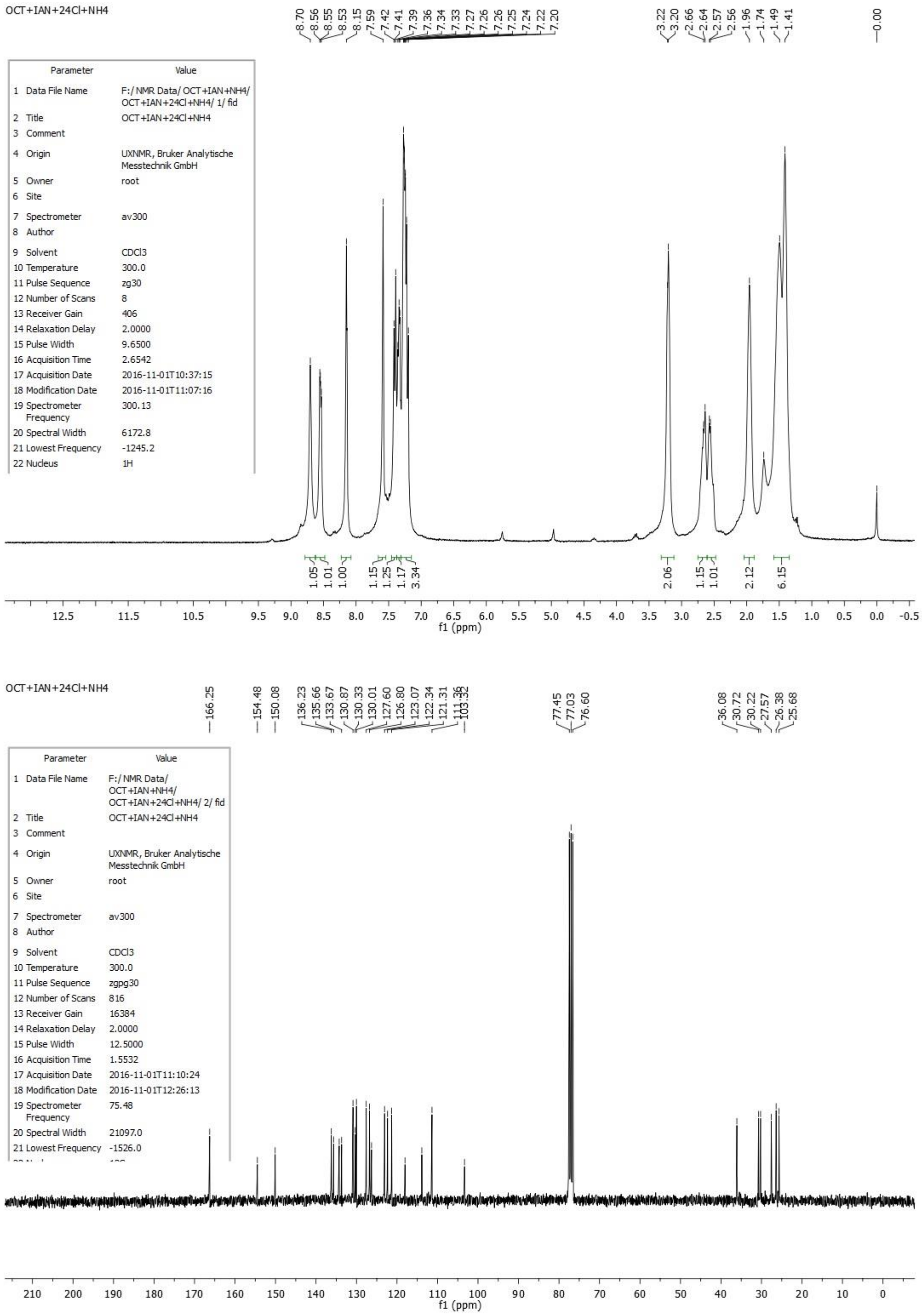

Figure 41: ^{1}H and ^{13}C NMR spectra of **15n**

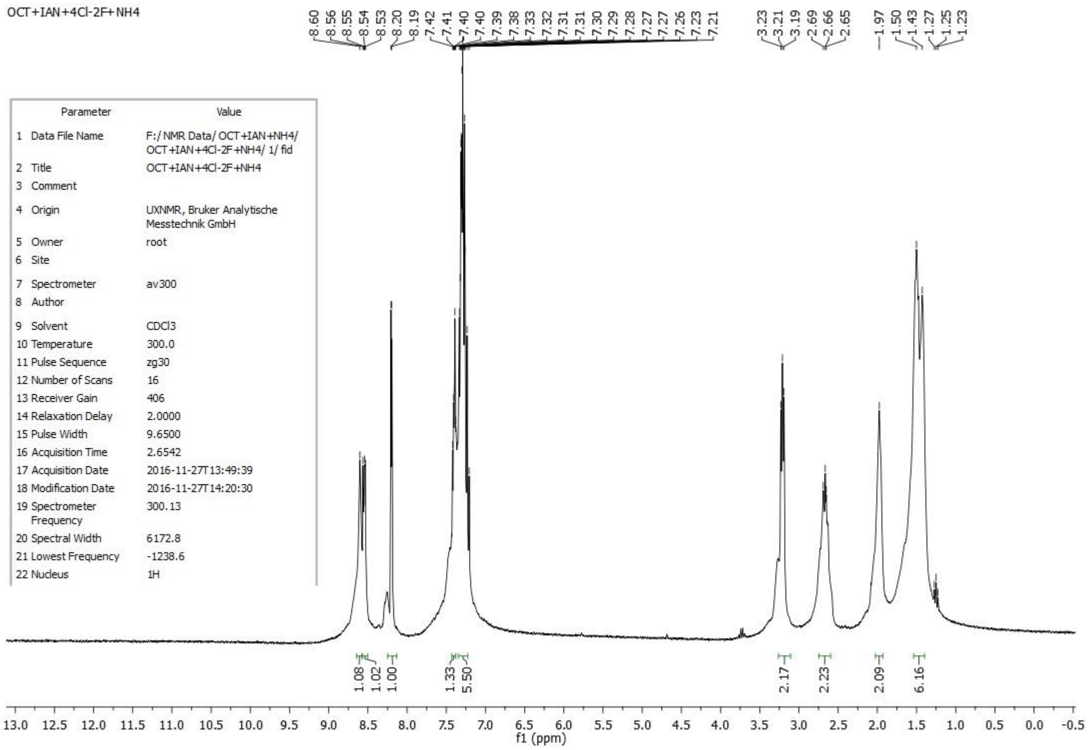

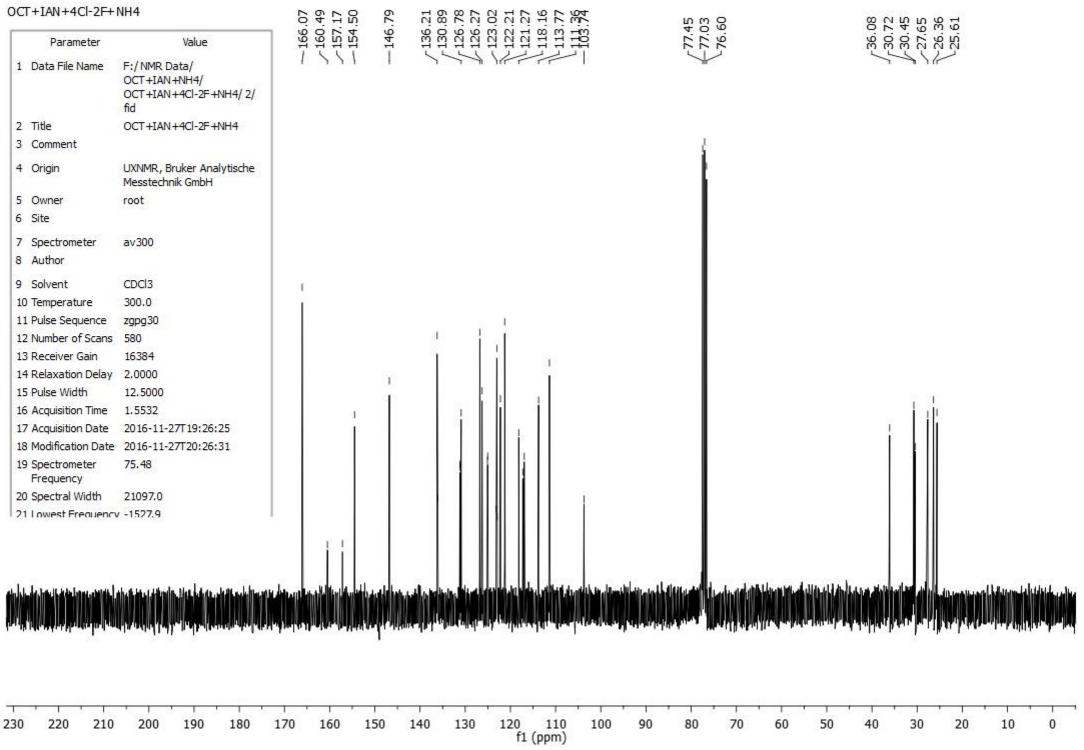

Figure 42: ^{1}H and ^{13}C NMR spectra of **15p**

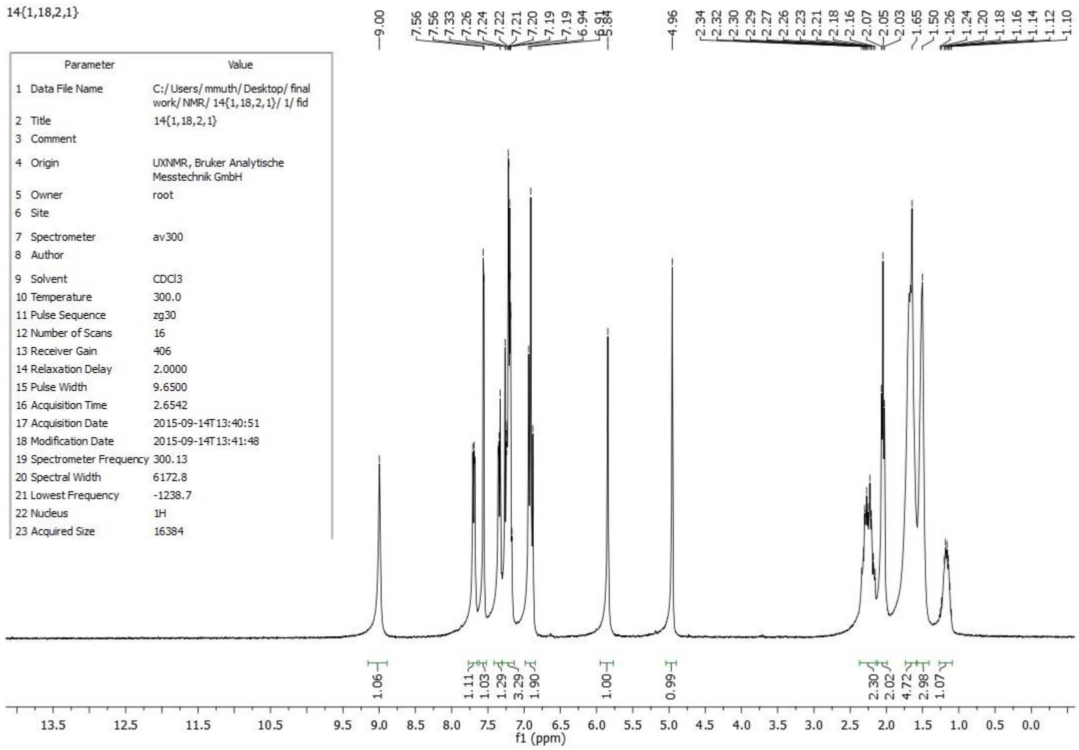

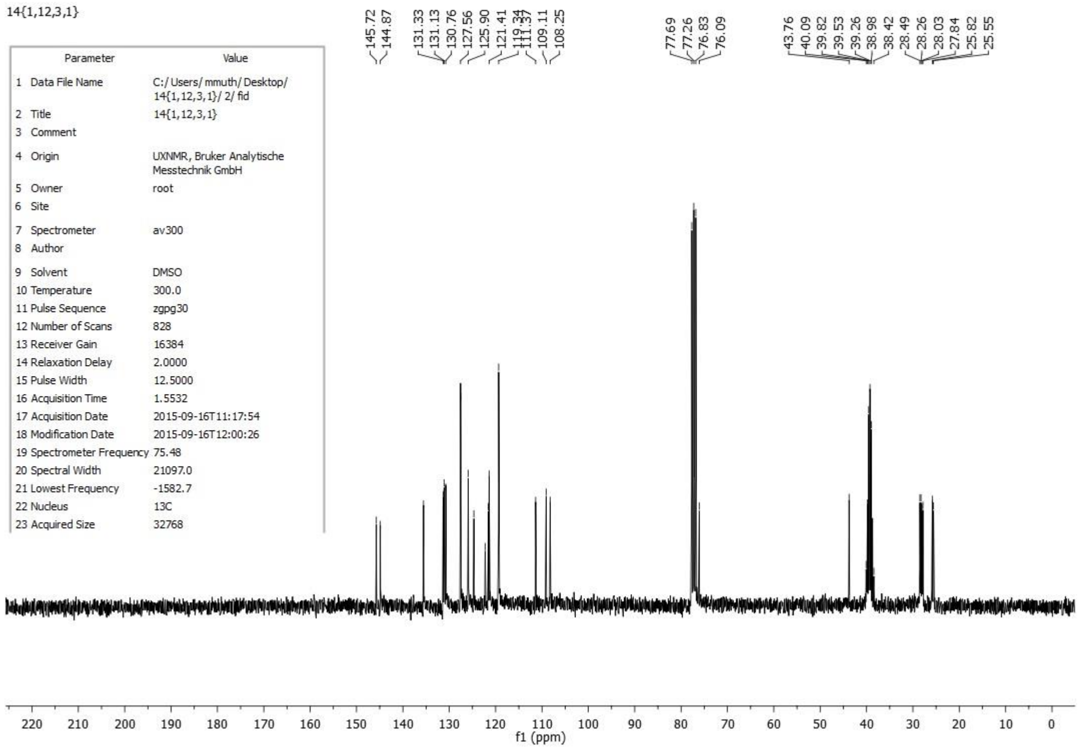

Figure 43: ^{1}H and ^{13}C NMR spectra of **15r**

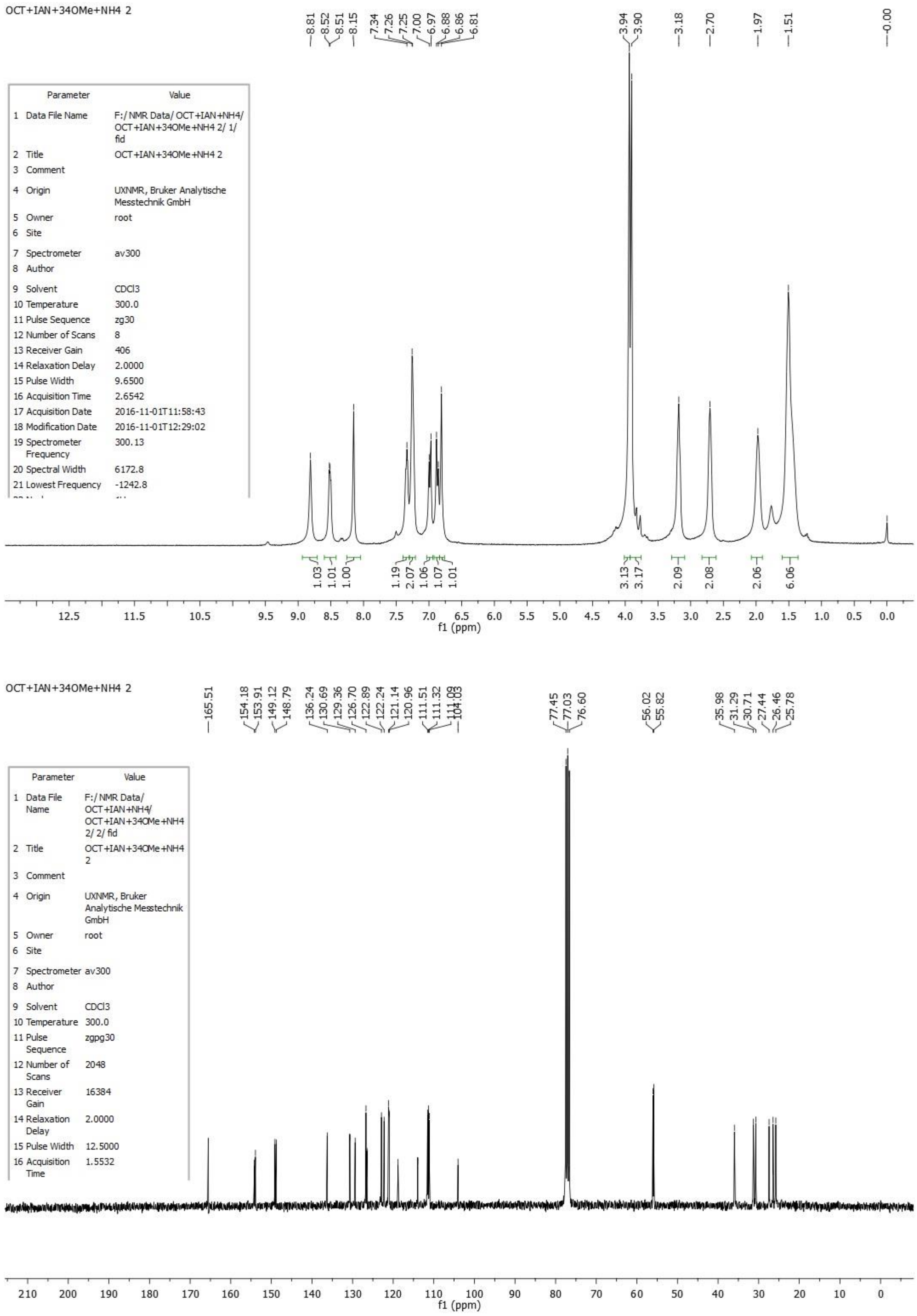

Figure 44: ^{1}H and ^{13}C NMR spectra of **15s**

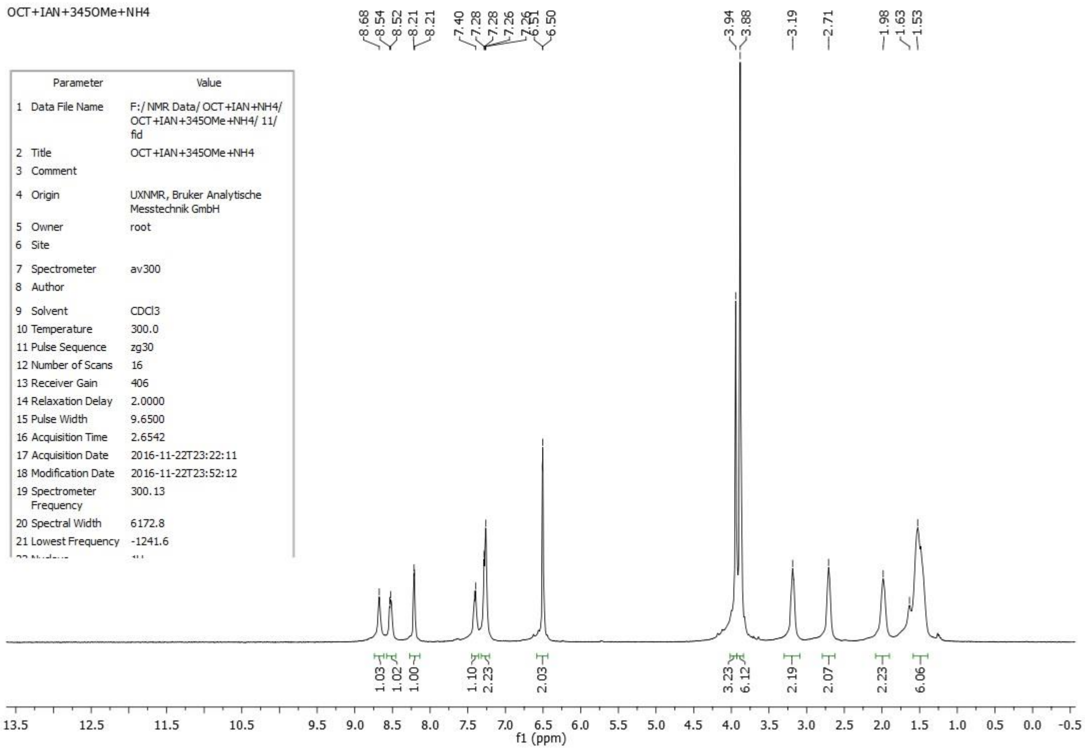

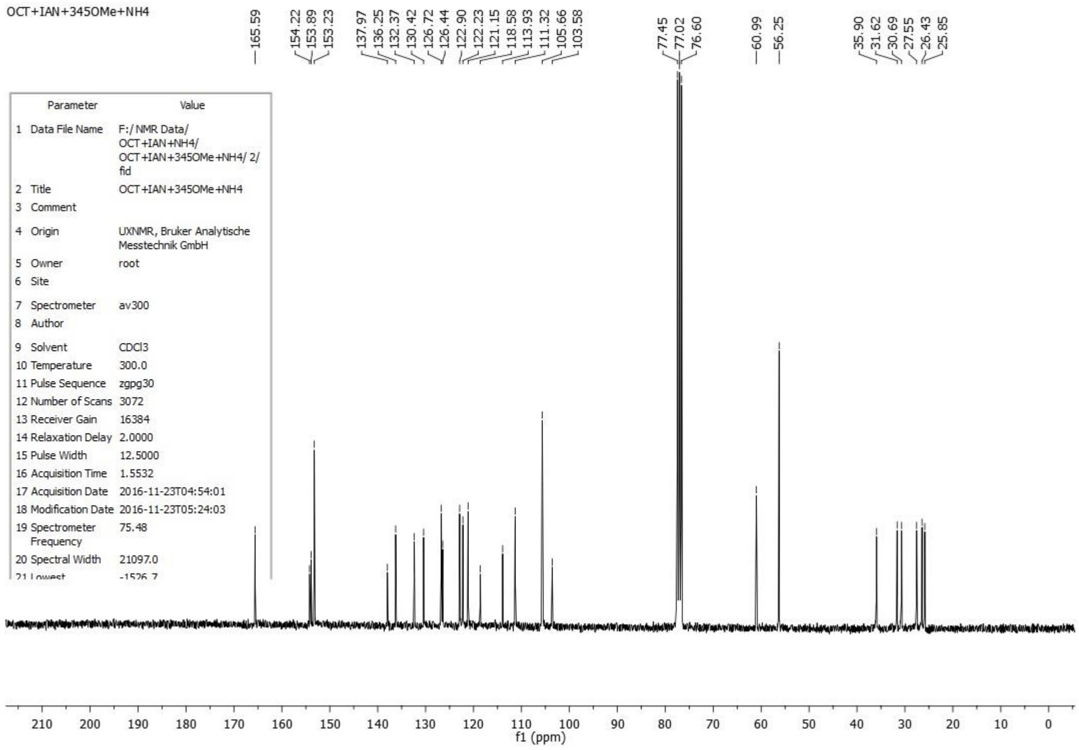

Figure 45: ^{1}H and ^{13}C NMR spectra of **15t**

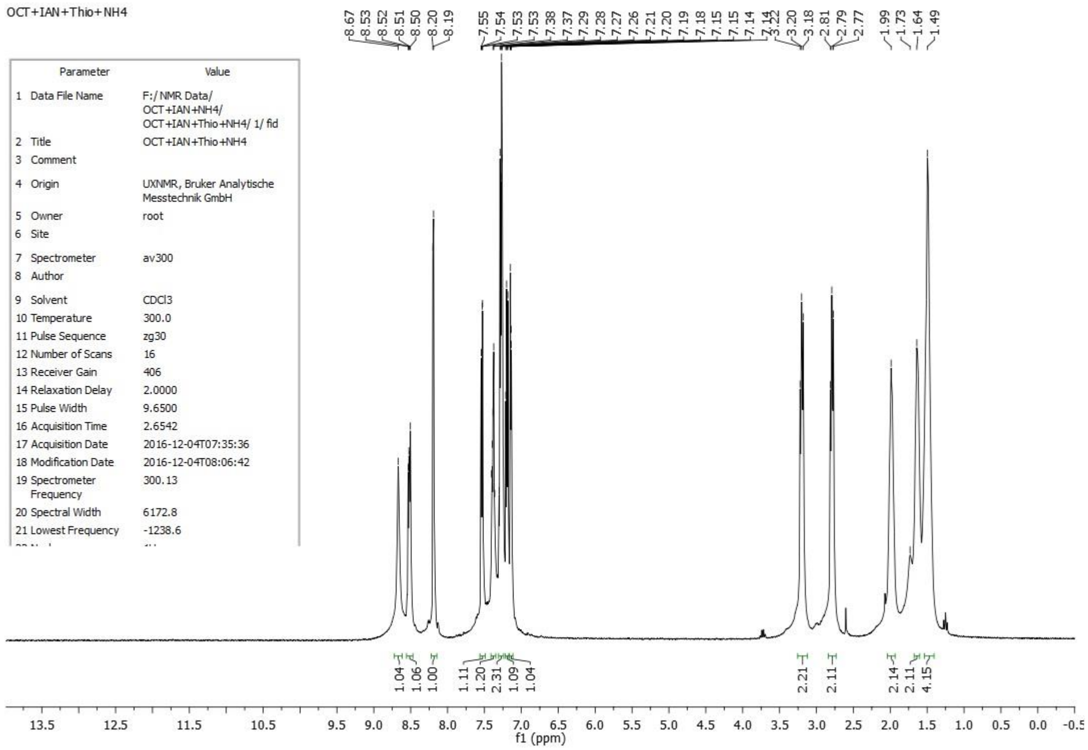

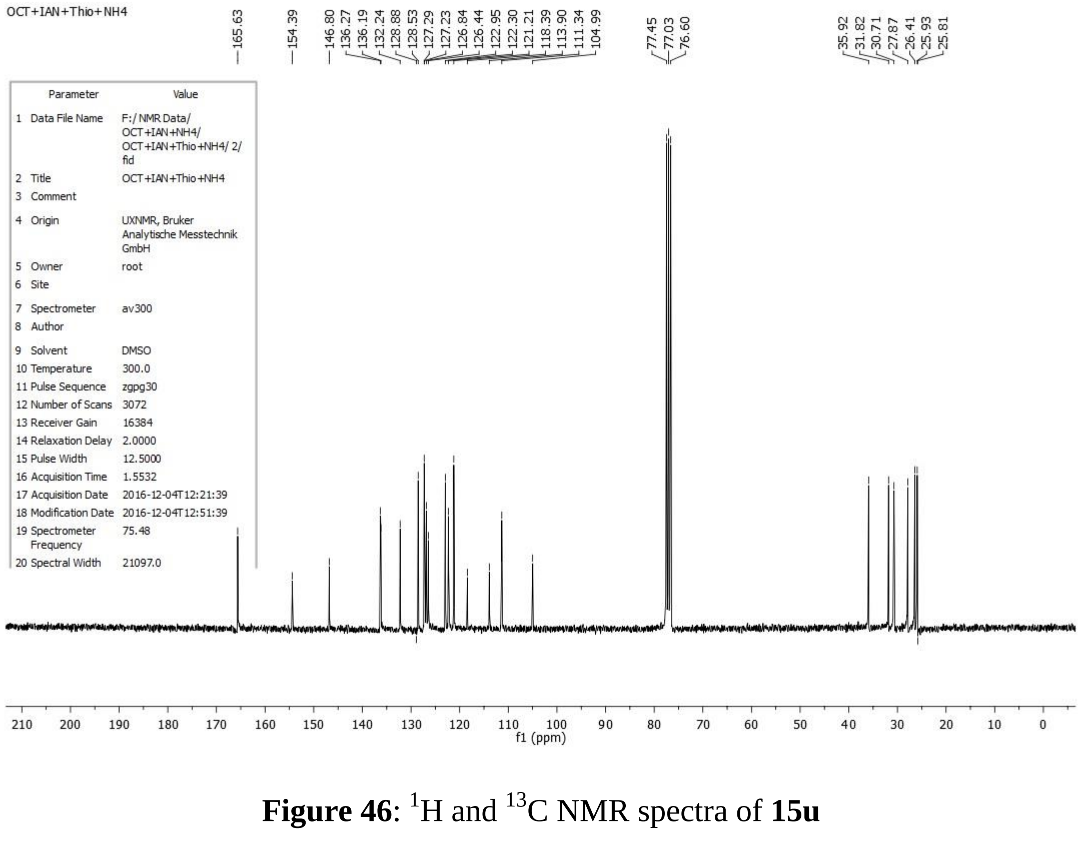

Figure 46: ^{1}H and ^{13}C NMR spectra of **15u**

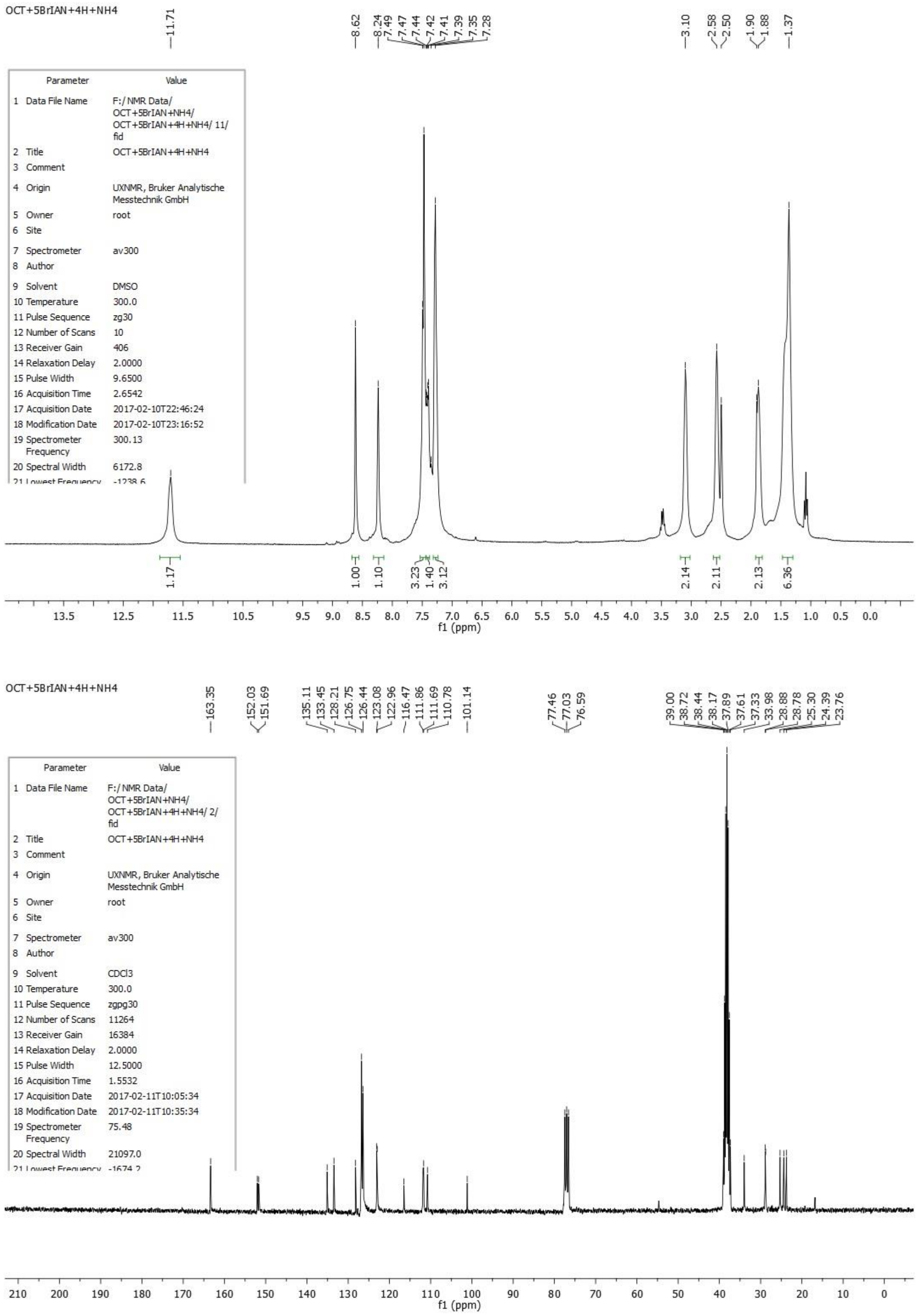

Figure 47: ^{1}H and ^{13}C NMR spectra of **16a**

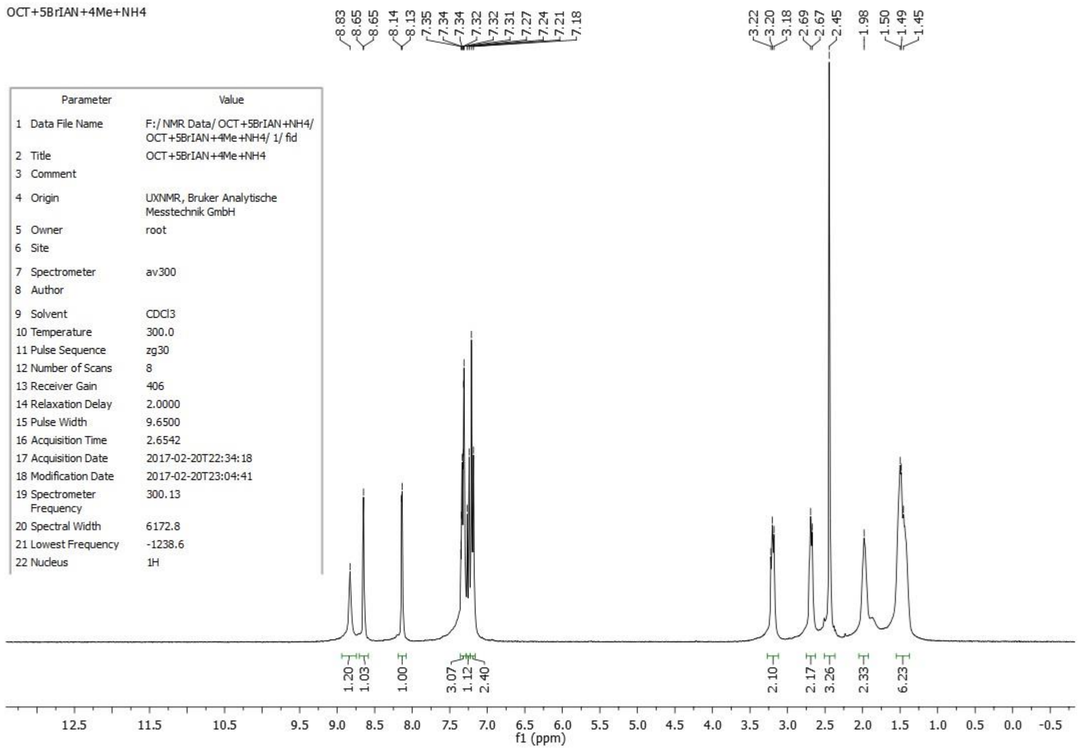

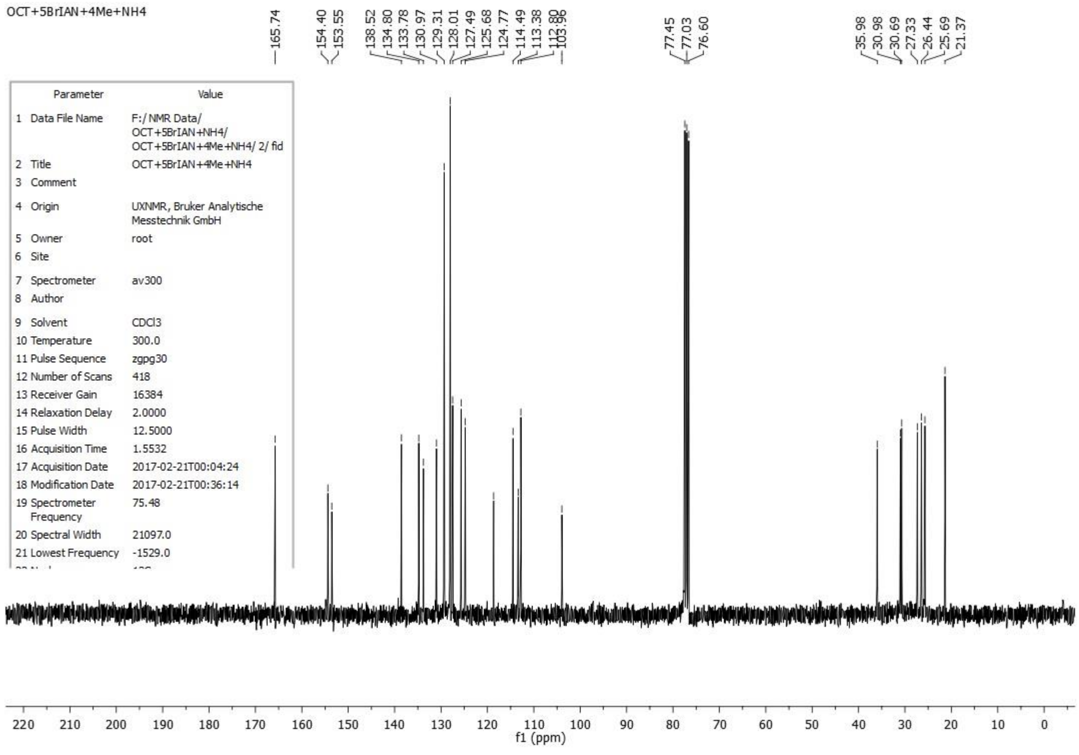

Figure 48: ^{1}H and ^{13}C NMR spectra of **16b**

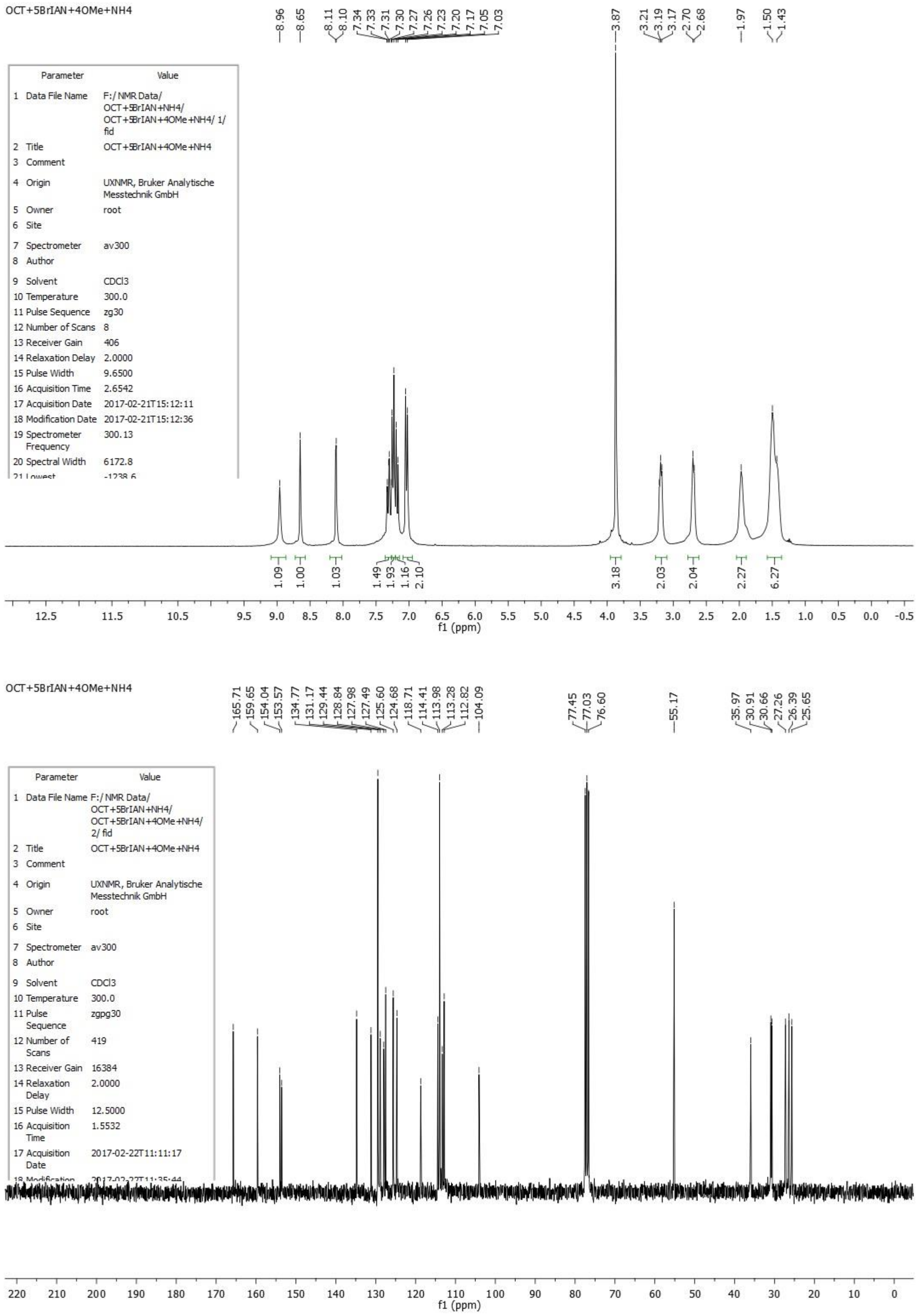

Figure 49: ^{1}H and ^{13}C NMR spectra of **16c**

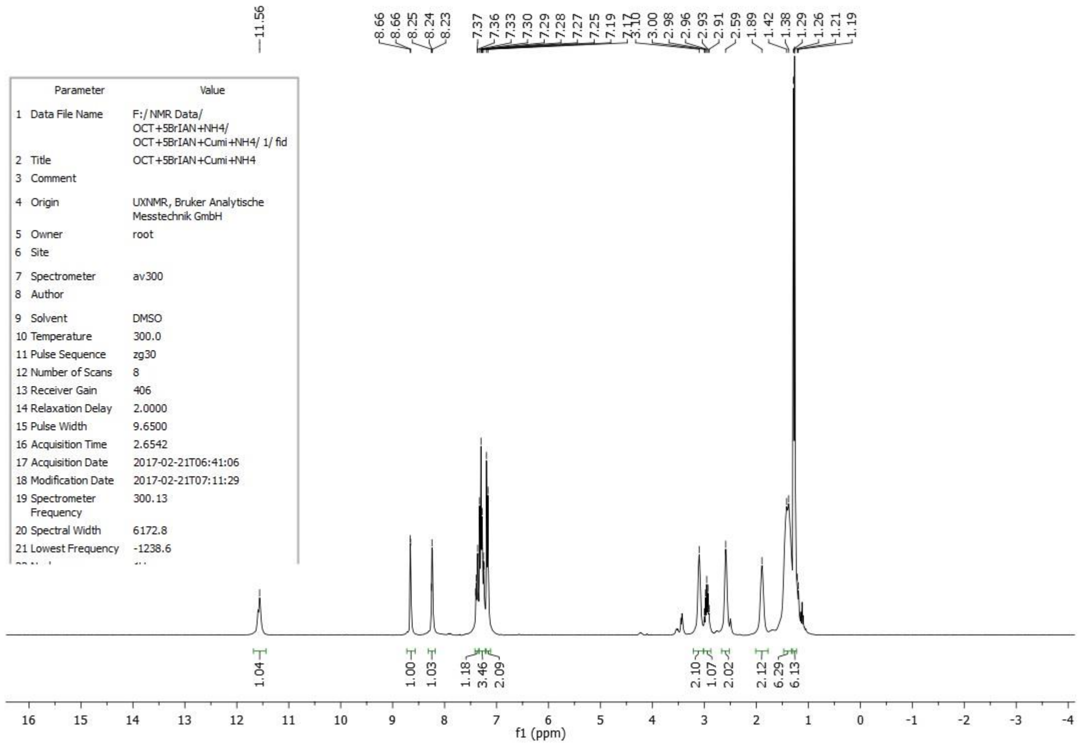

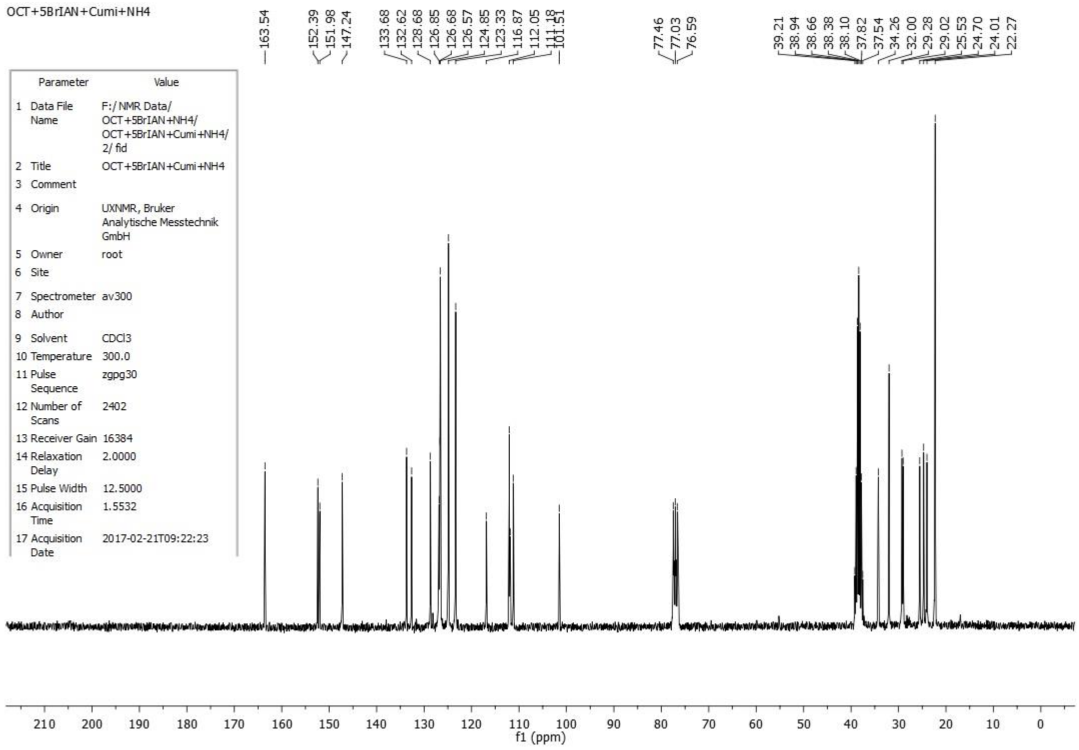

Figure 50: ^{1}H and ^{13}C NMR spectra of **16d**

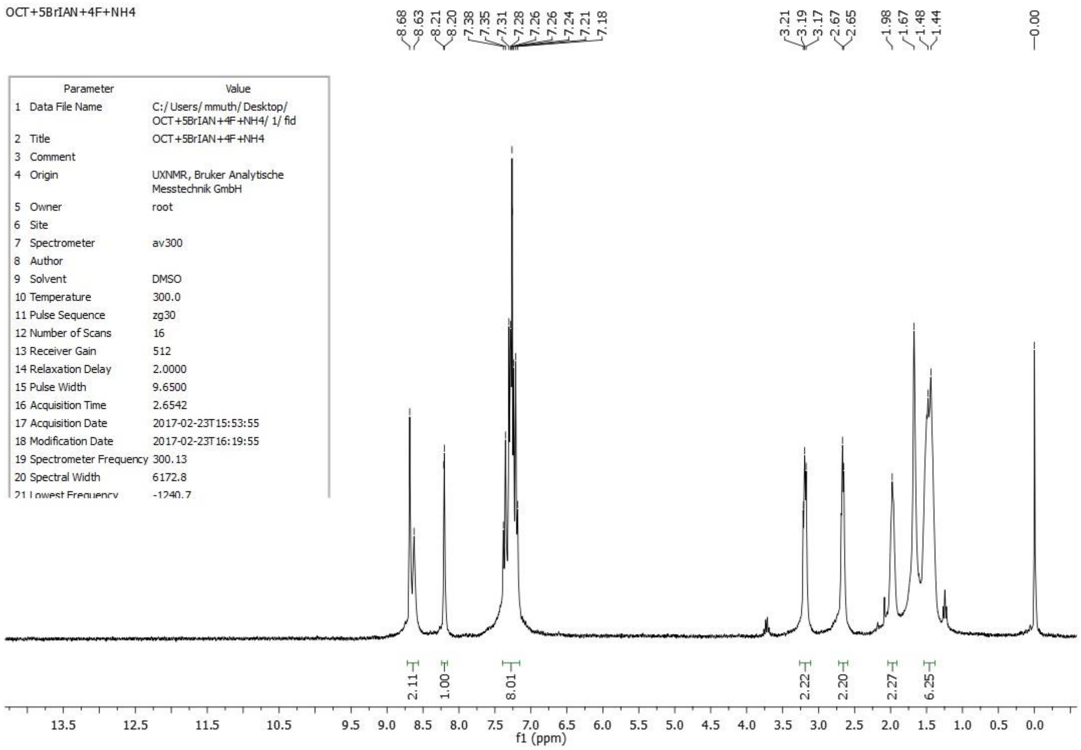

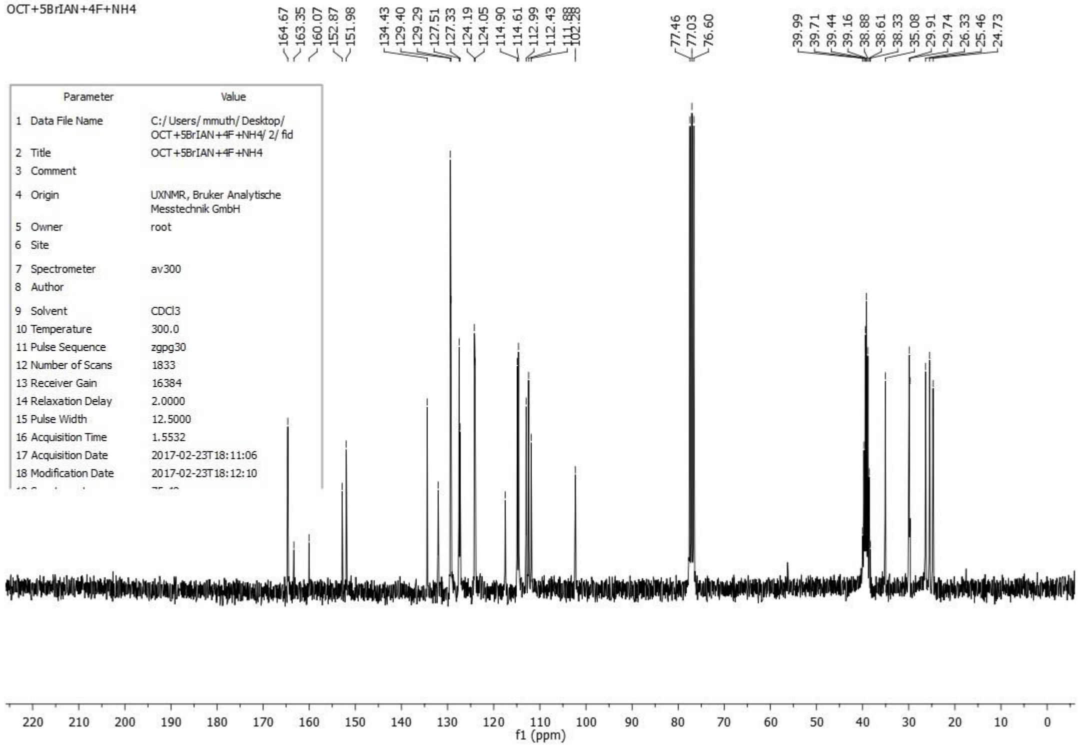

Figure 51: ^{1}H and ^{13}C NMR spectra of **16e**

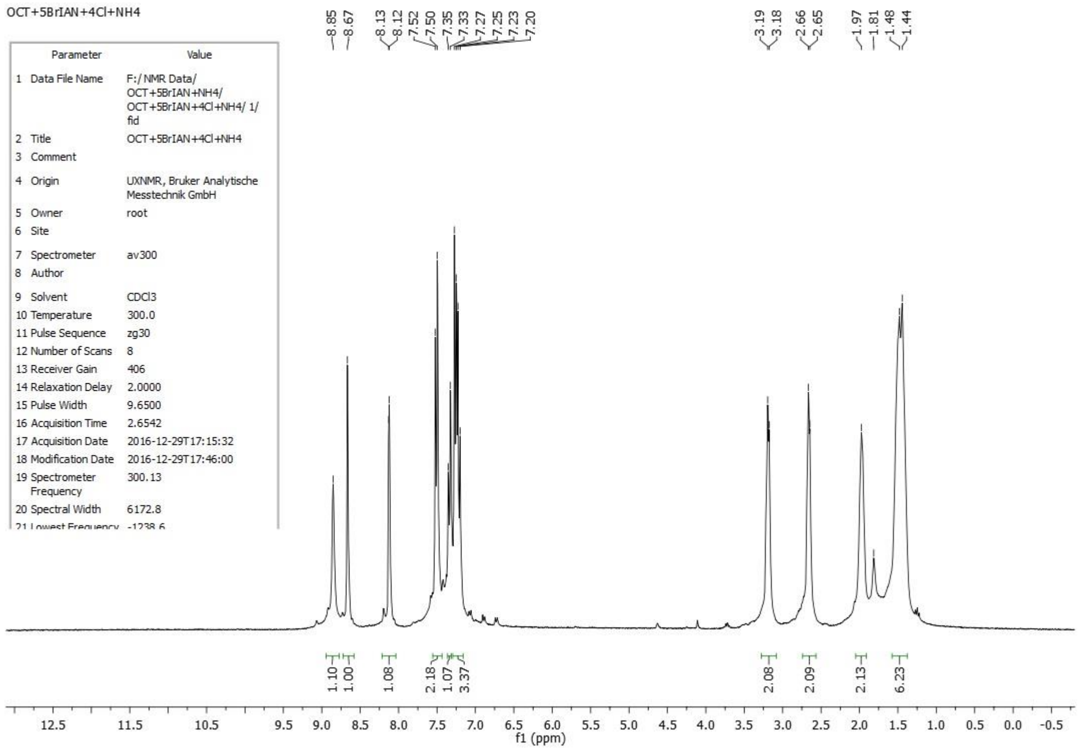

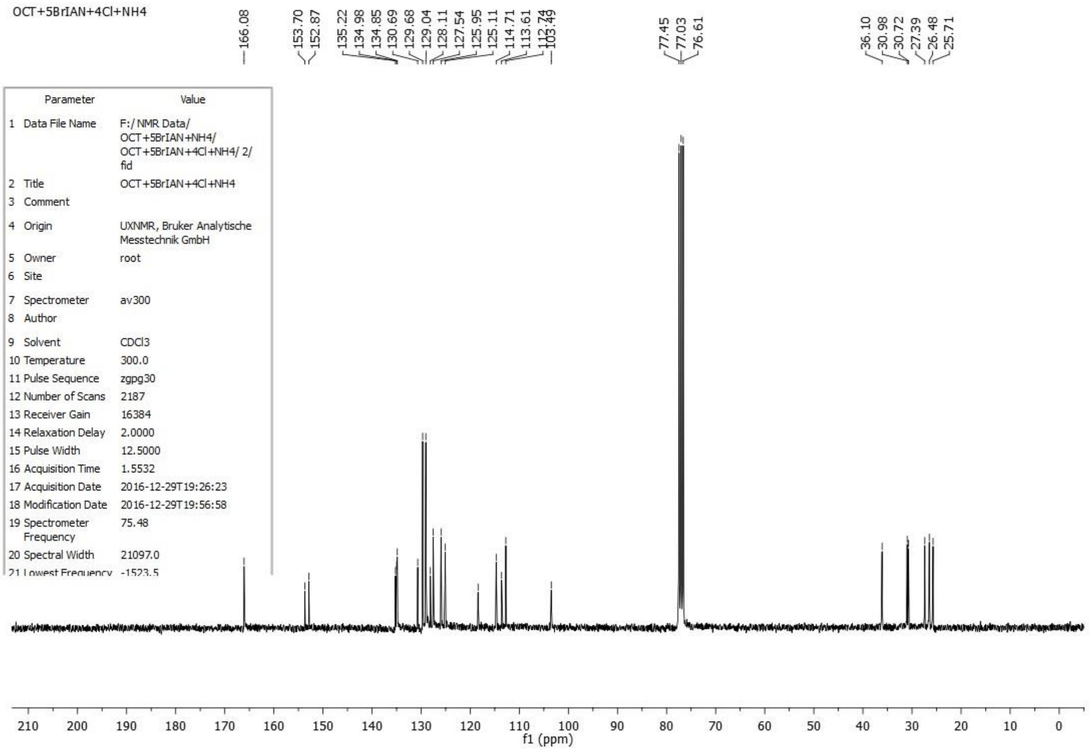

Figure 52: ^{1}H and ^{13}C NMR spectra of **16f**

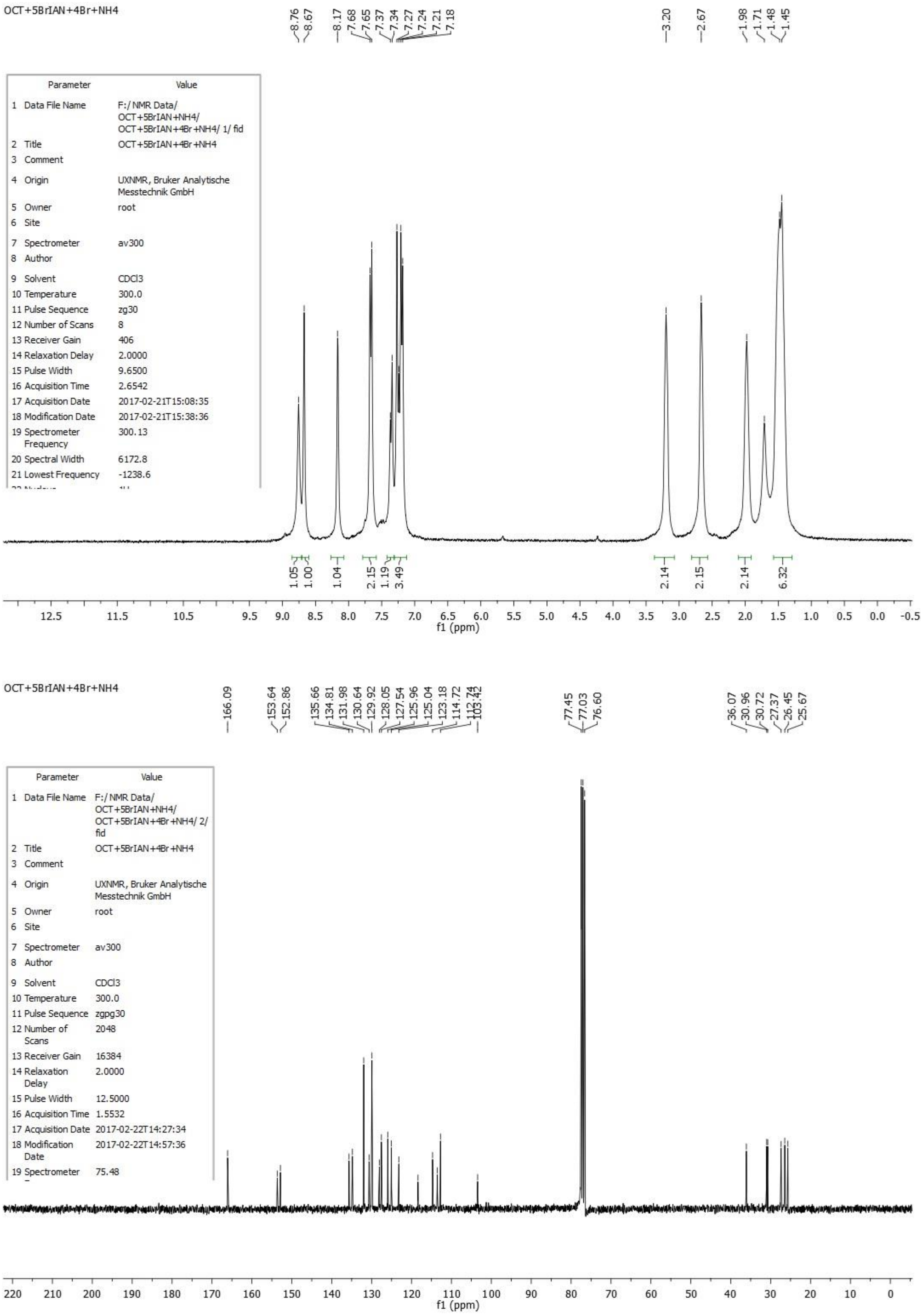

Figure 53: ^{1}H and ^{13}C NMR spectra of **16g**

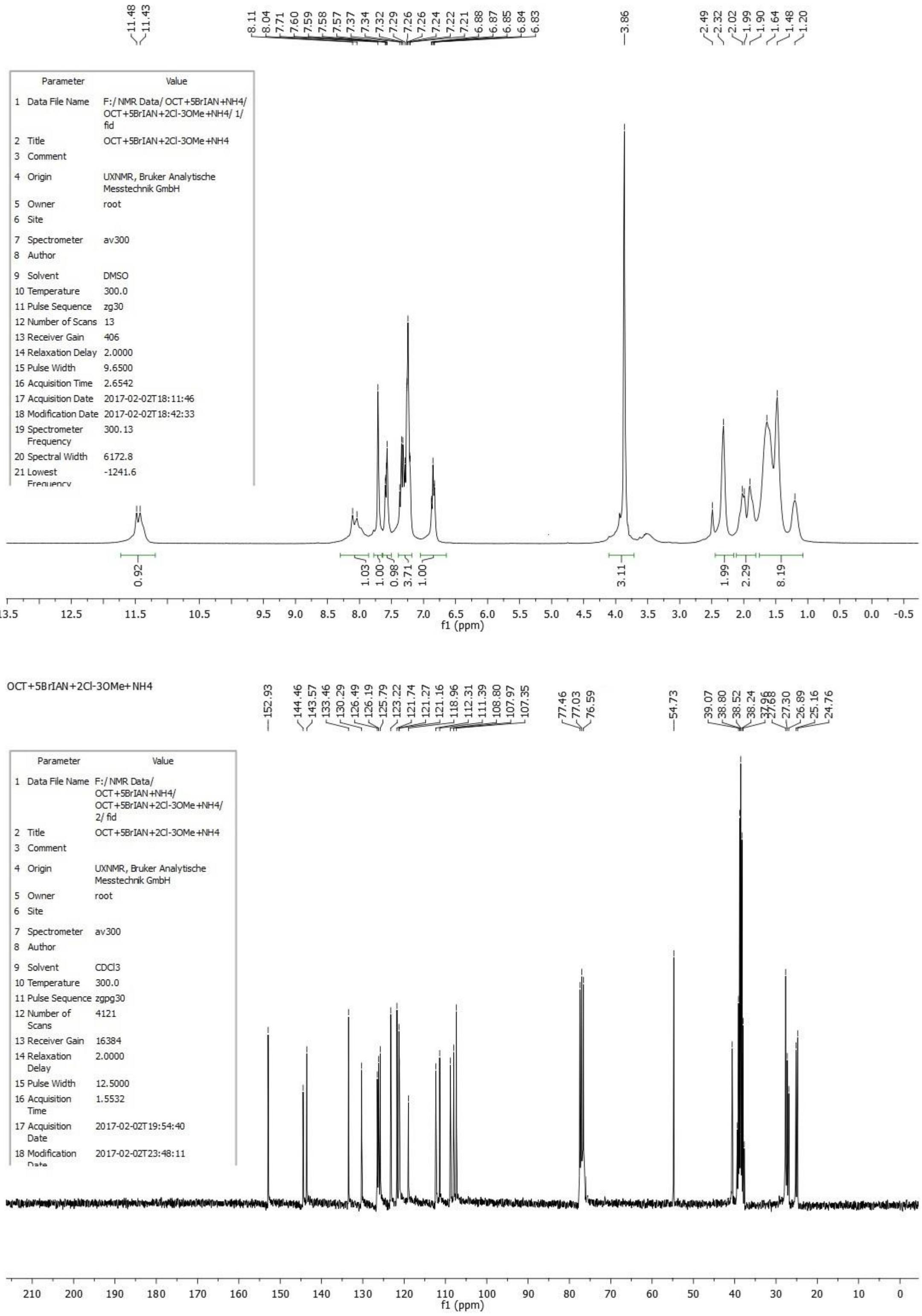

Figure 54: ^{1}H and ^{13}C NMR spectra of **16o**

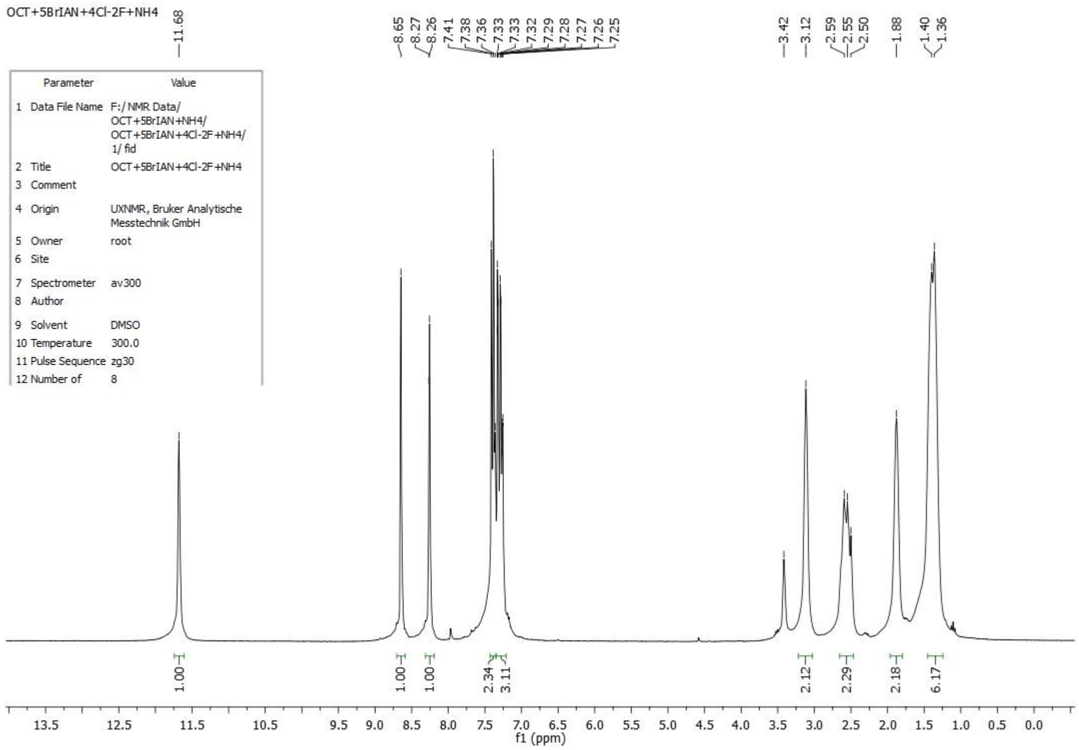

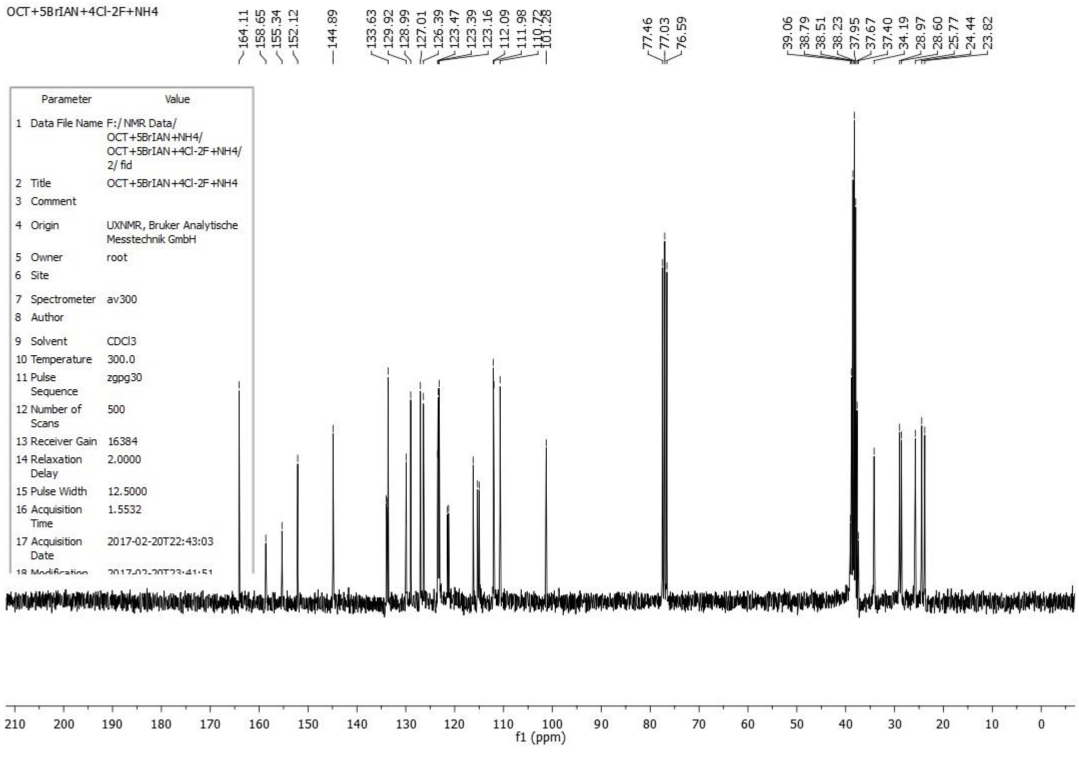

Figure 55: ^{1}H and ^{13}C NMR spectra of **16p**

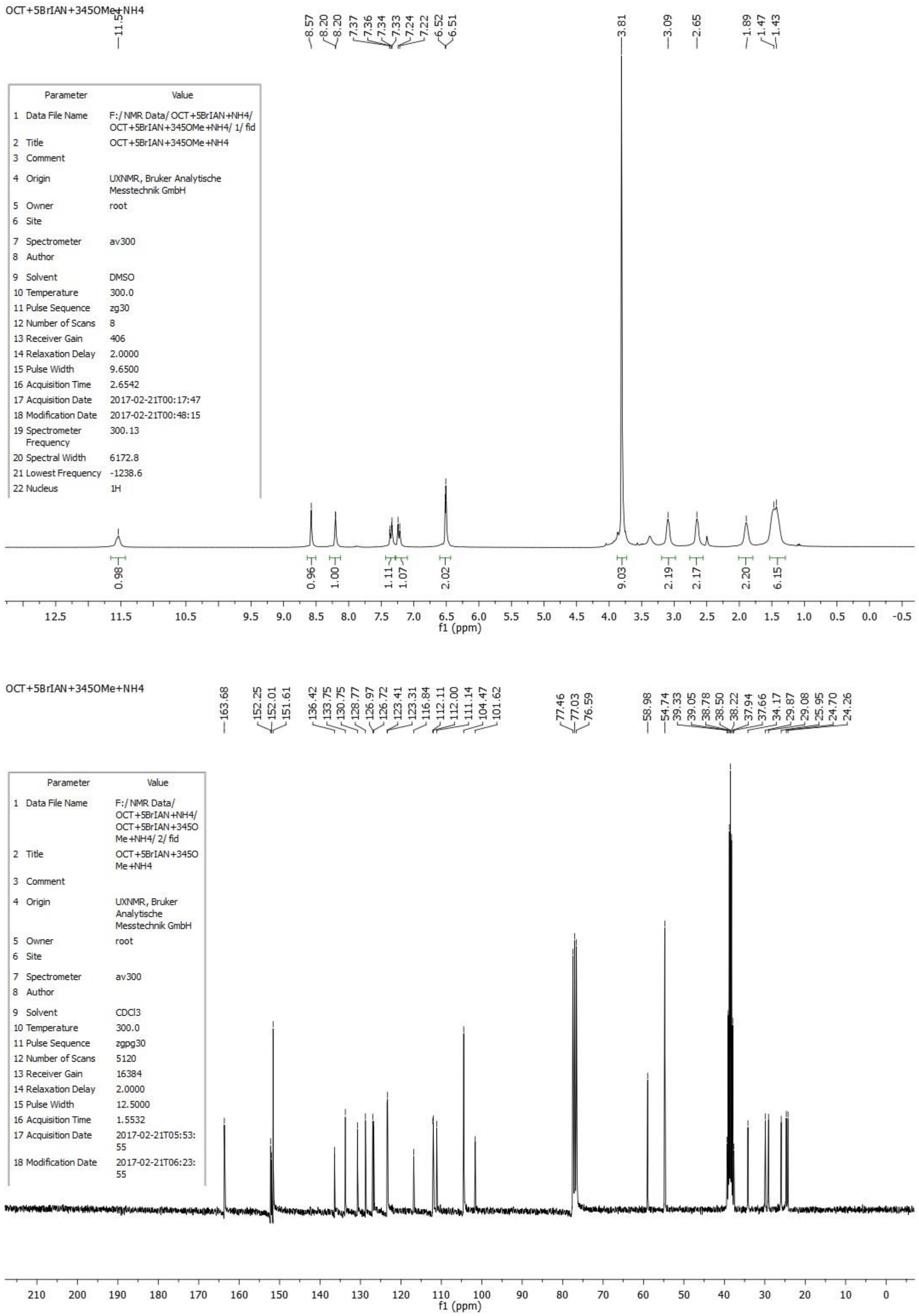

Figure 56: ^{1}H and ^{13}C NMR spectra of **16t**

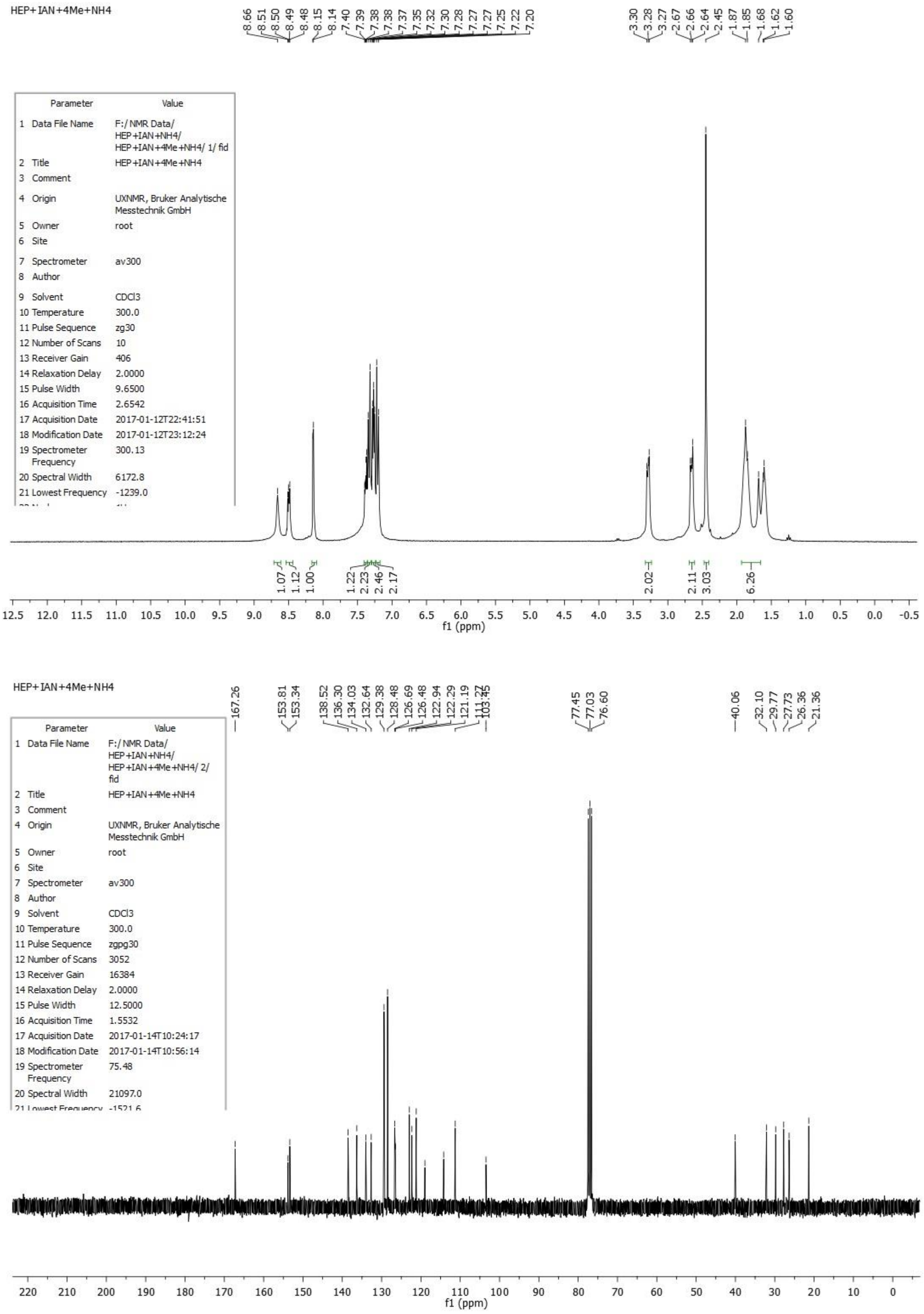

Figure 57: ^{1}H and ^{13}C NMR spectra of **17b**

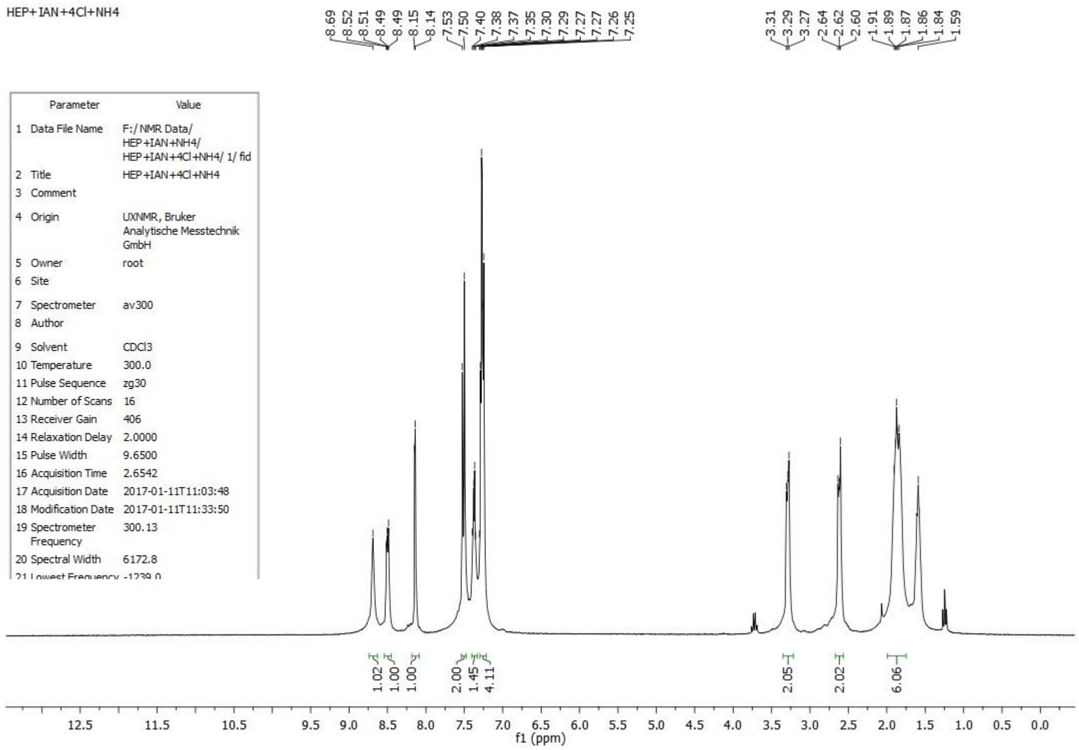

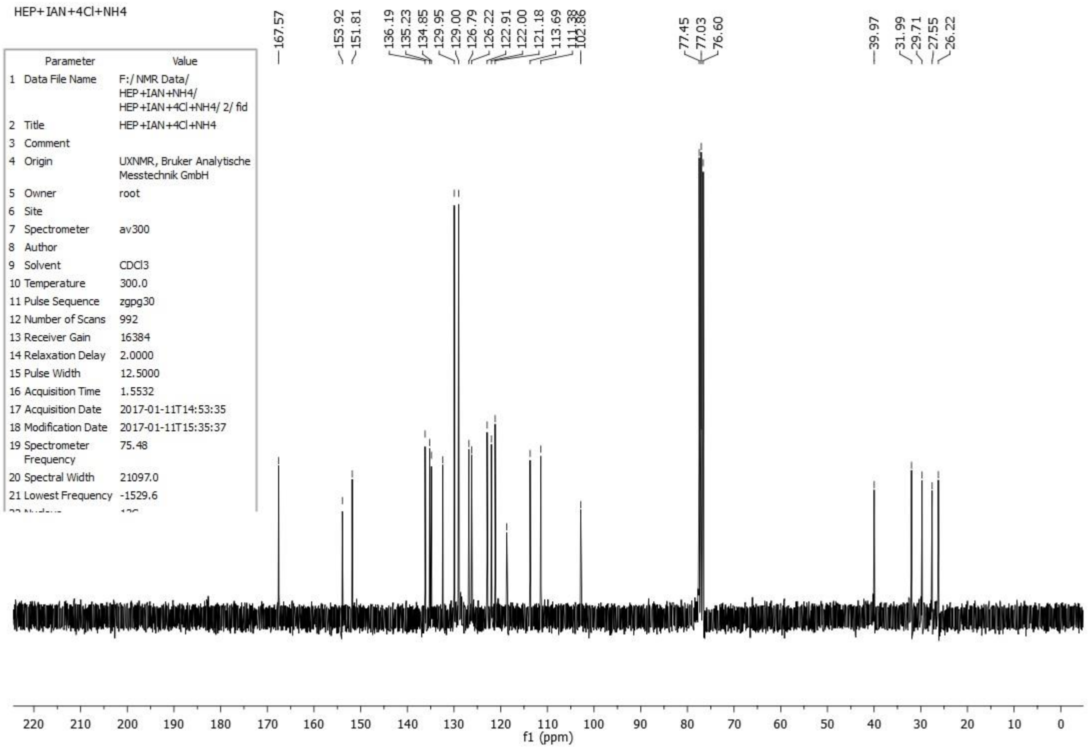

Figure 58: ^{1}H and ^{13}C NMR spectra of **17f**

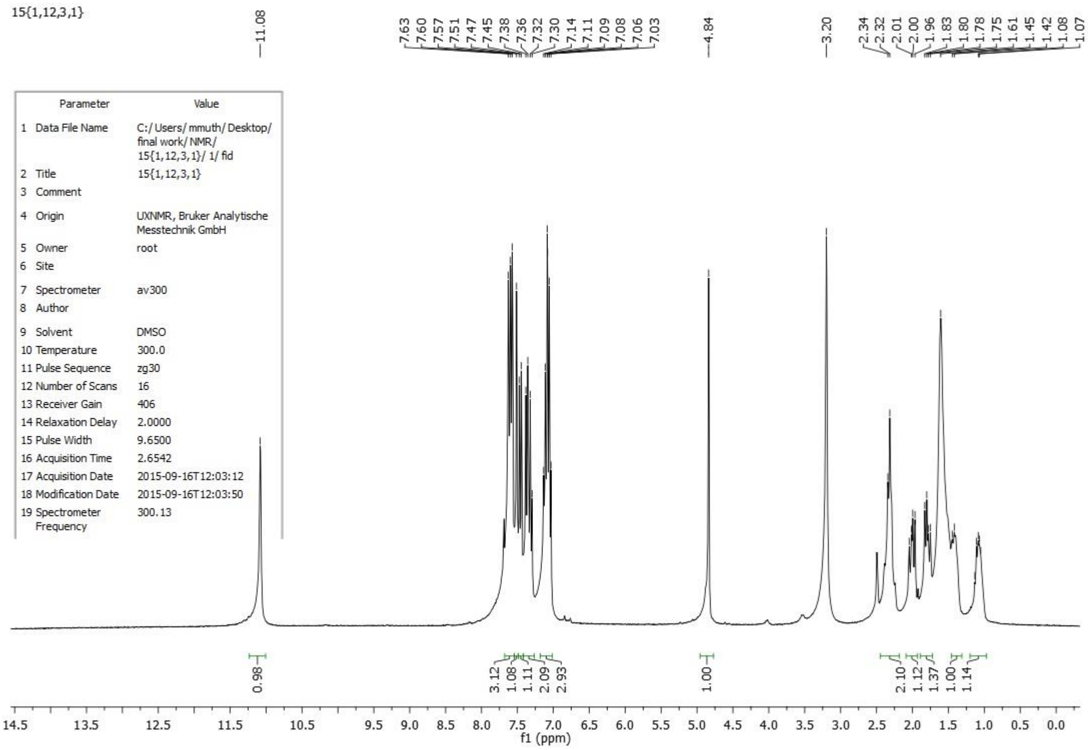

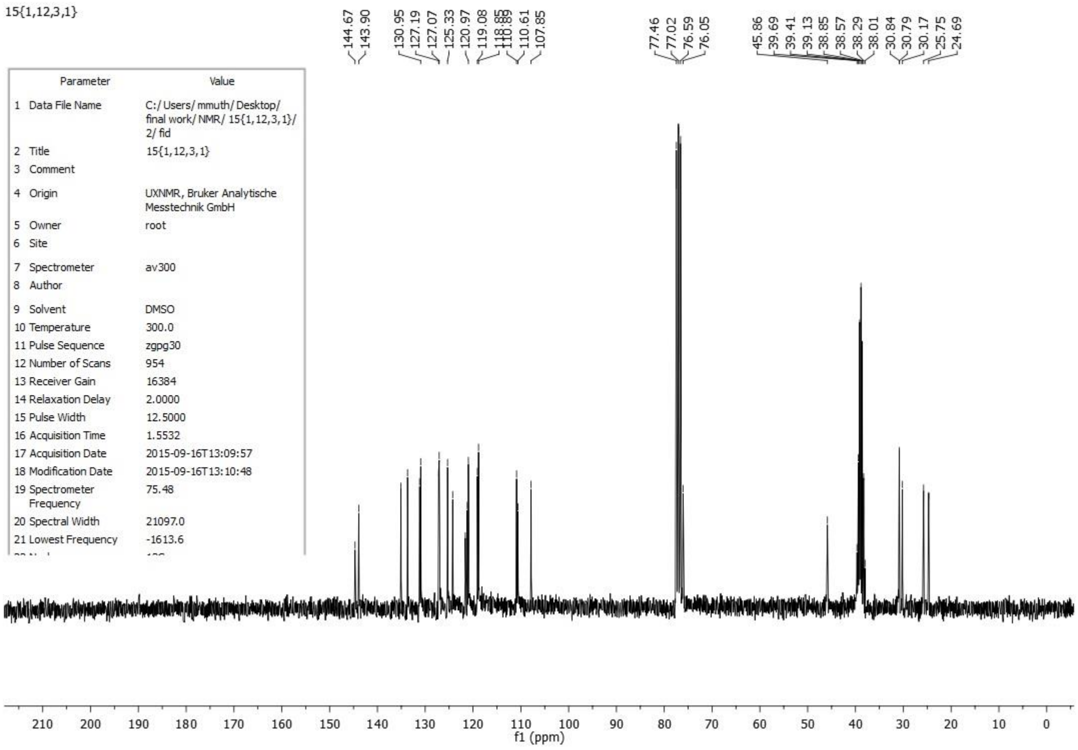

Figure 59: ^{1}H and ^{13}C NMR spectra of **17l**

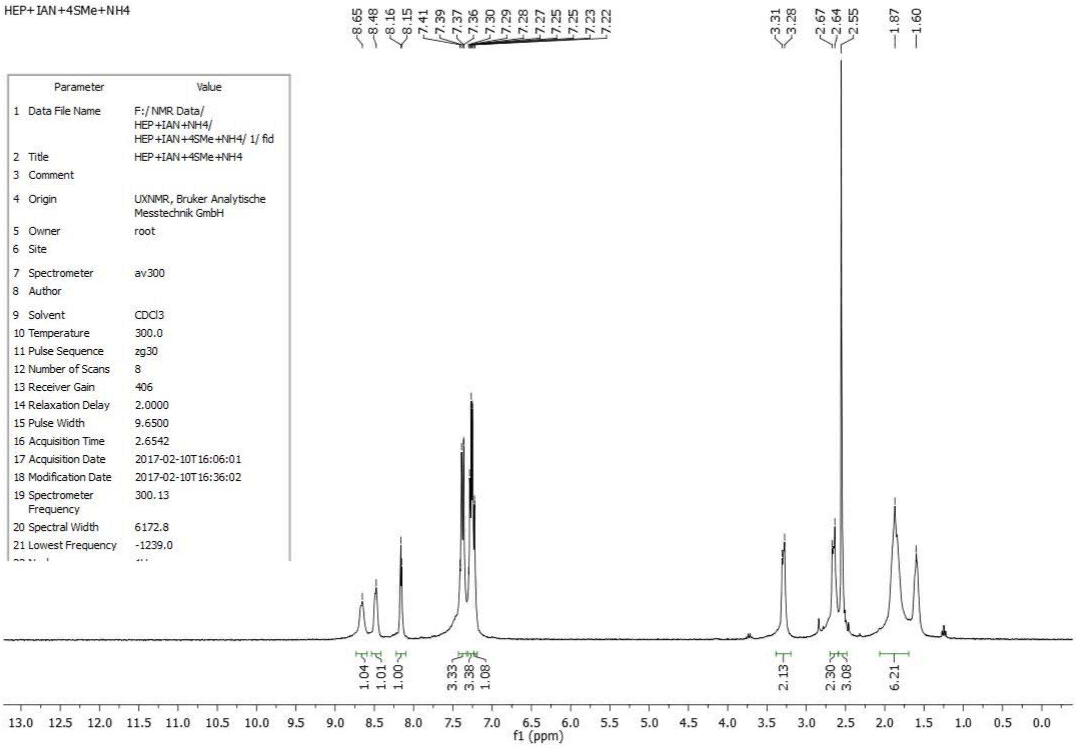

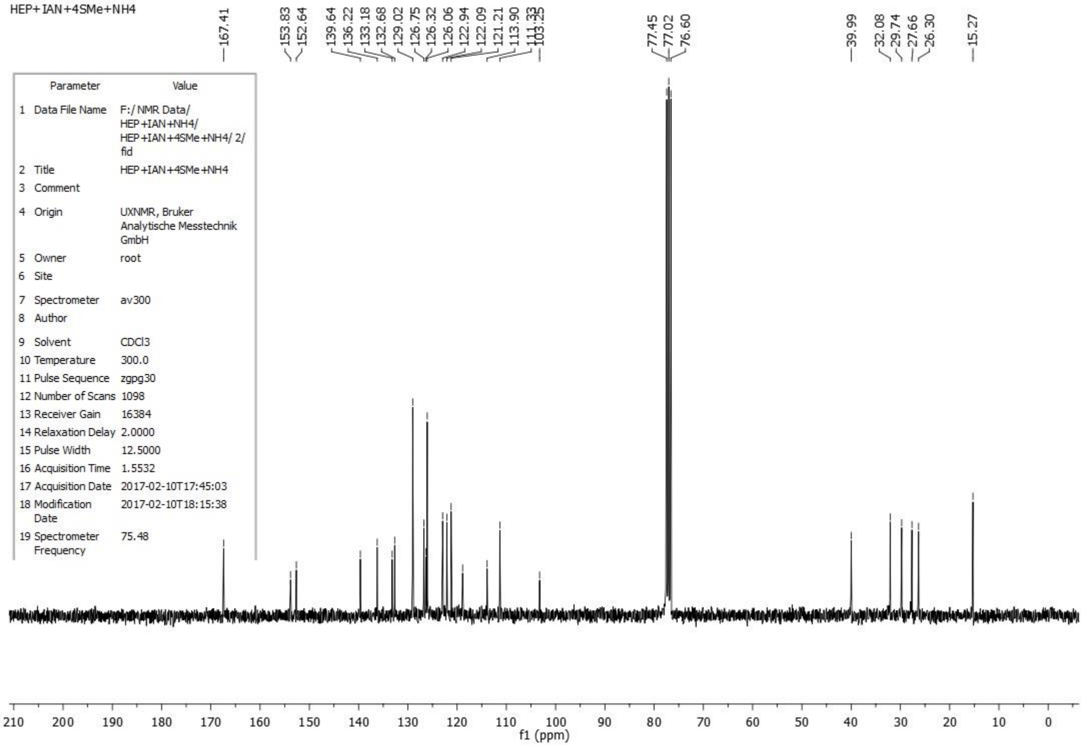

Figure 60: ^{1}H and ^{13}C NMR spectra of **17v**

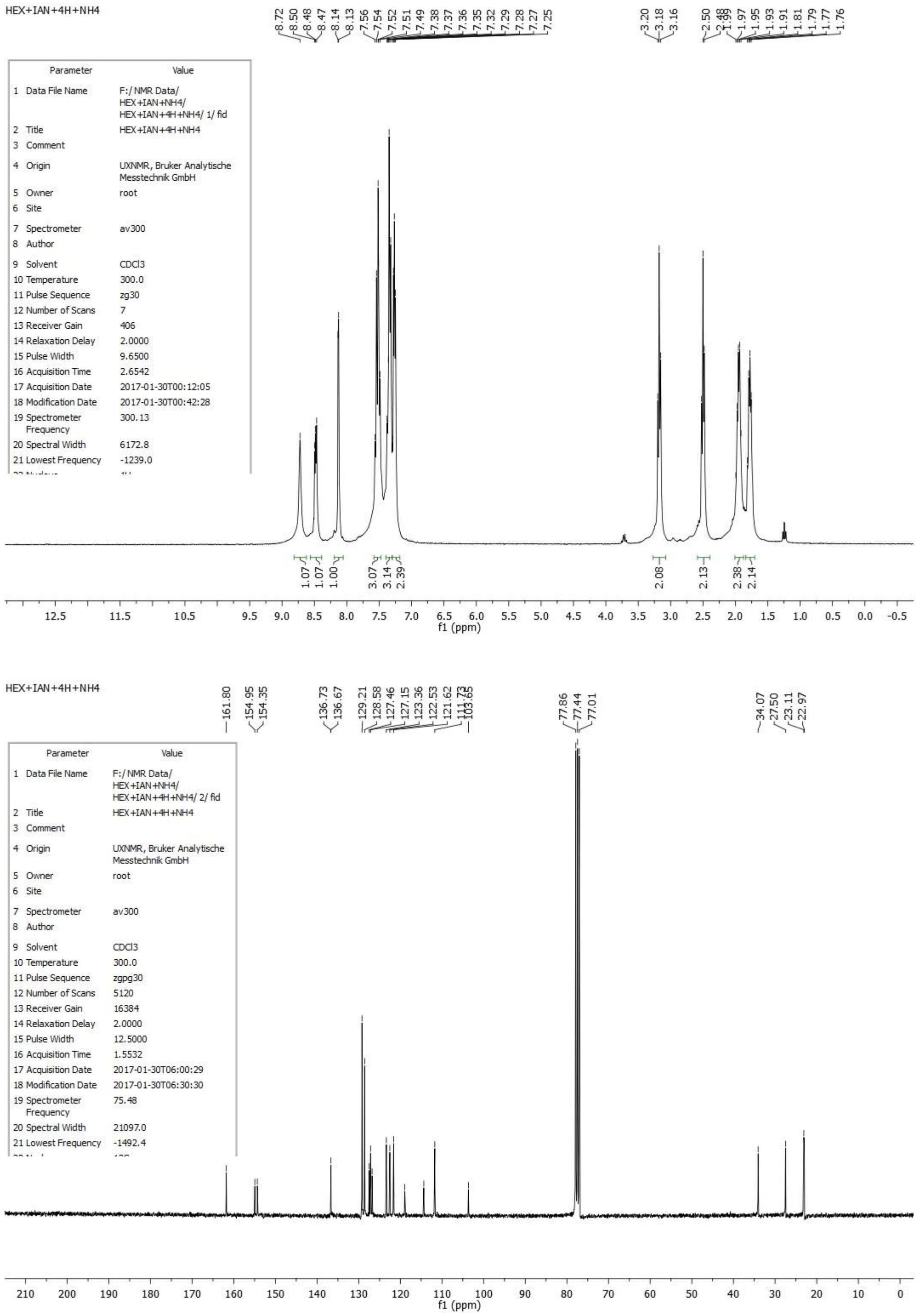

Figure 61: ^{1}H and ^{13}C NMR spectra of **18a**

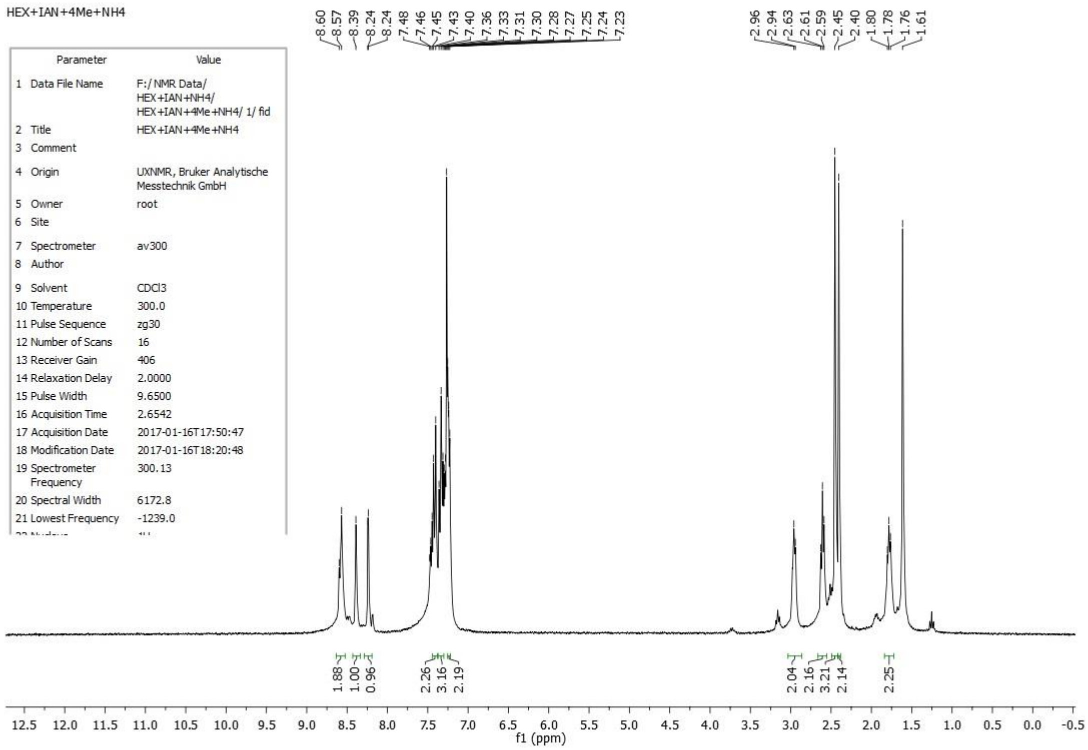

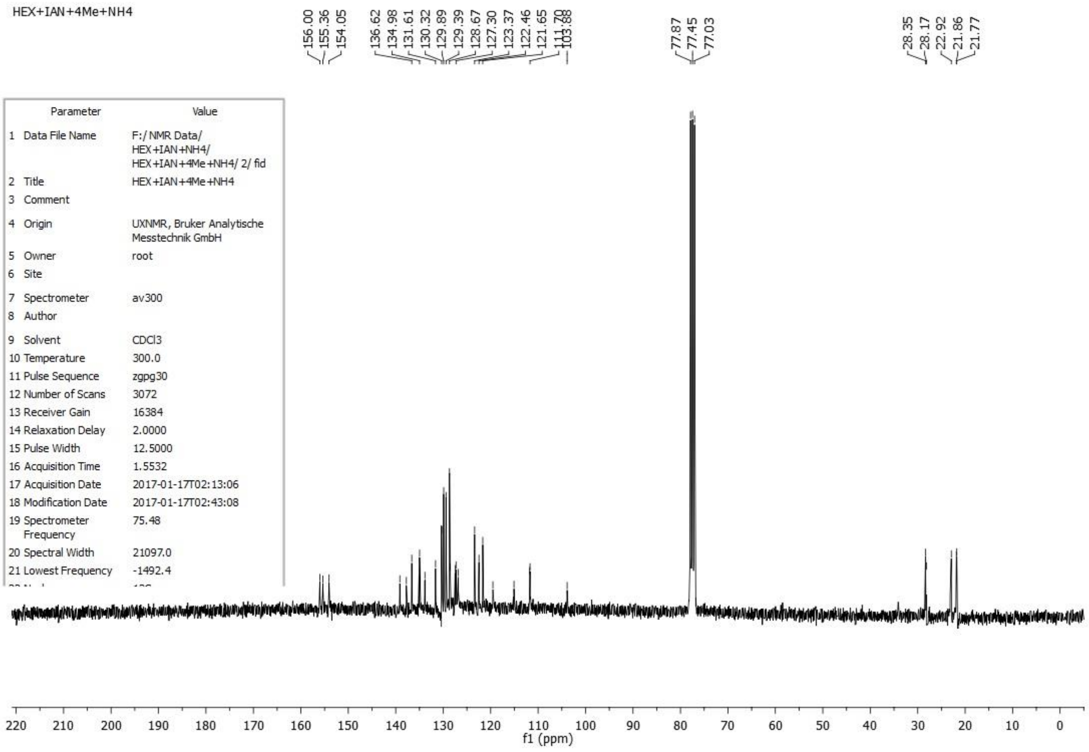

Figure 62: ^{1}H and ^{13}C NMR spectra of **18b**

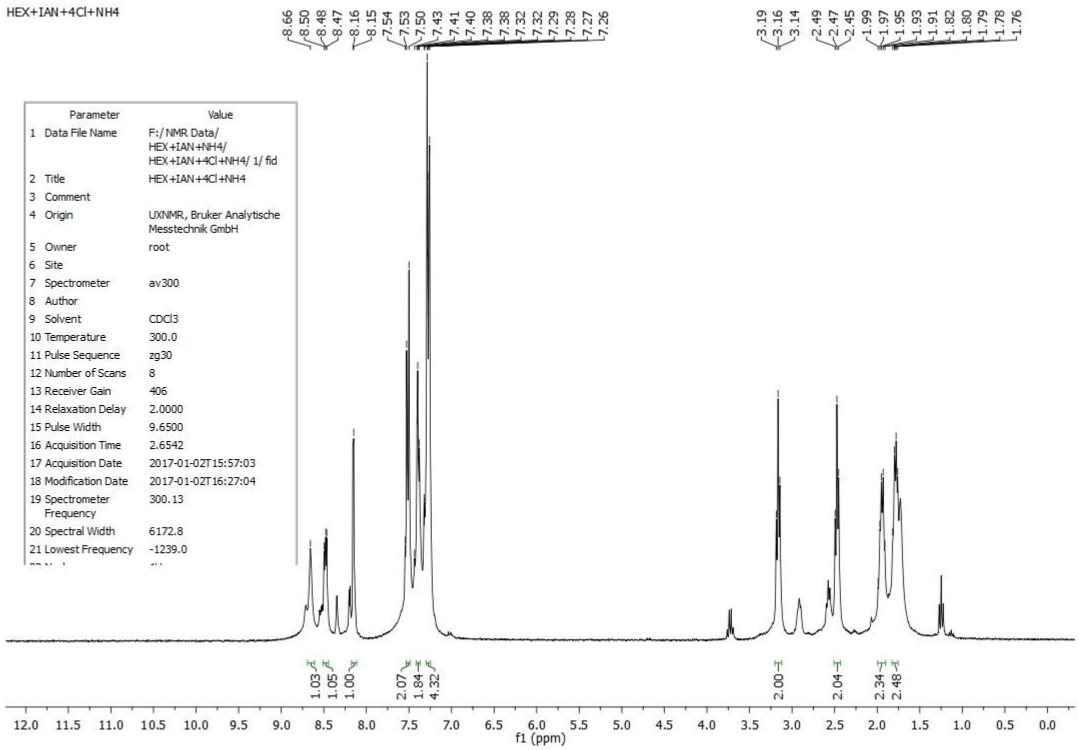

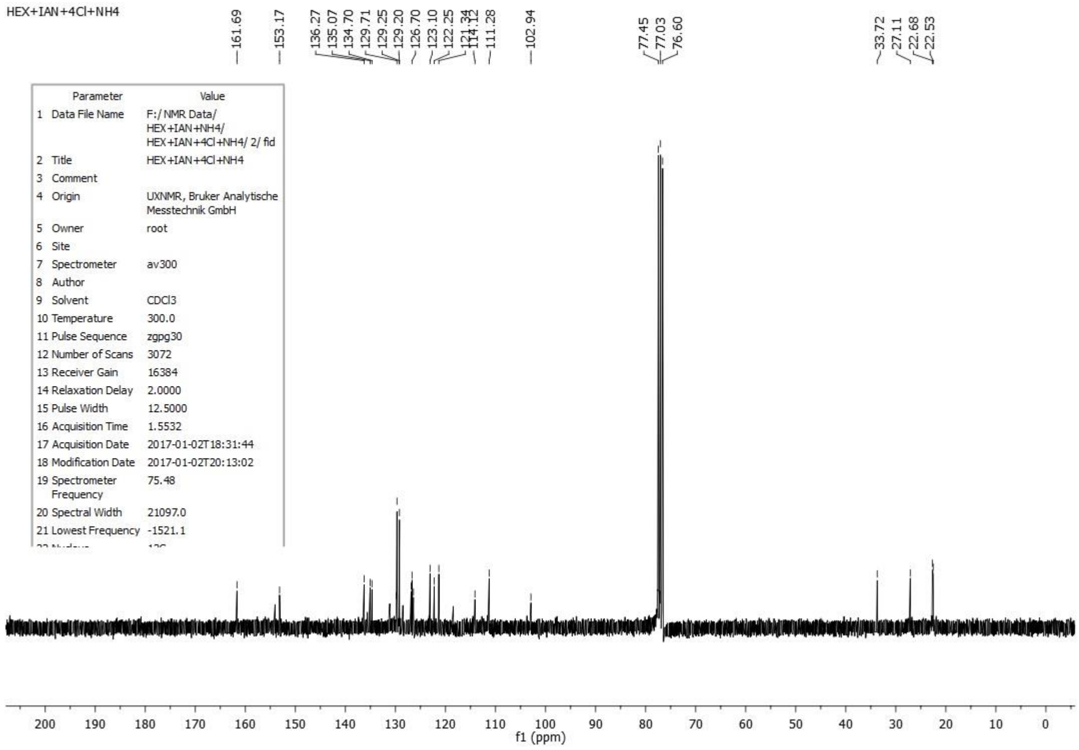

Figure 63: ^{1}H and ^{13}C NMR spectra of **18f**

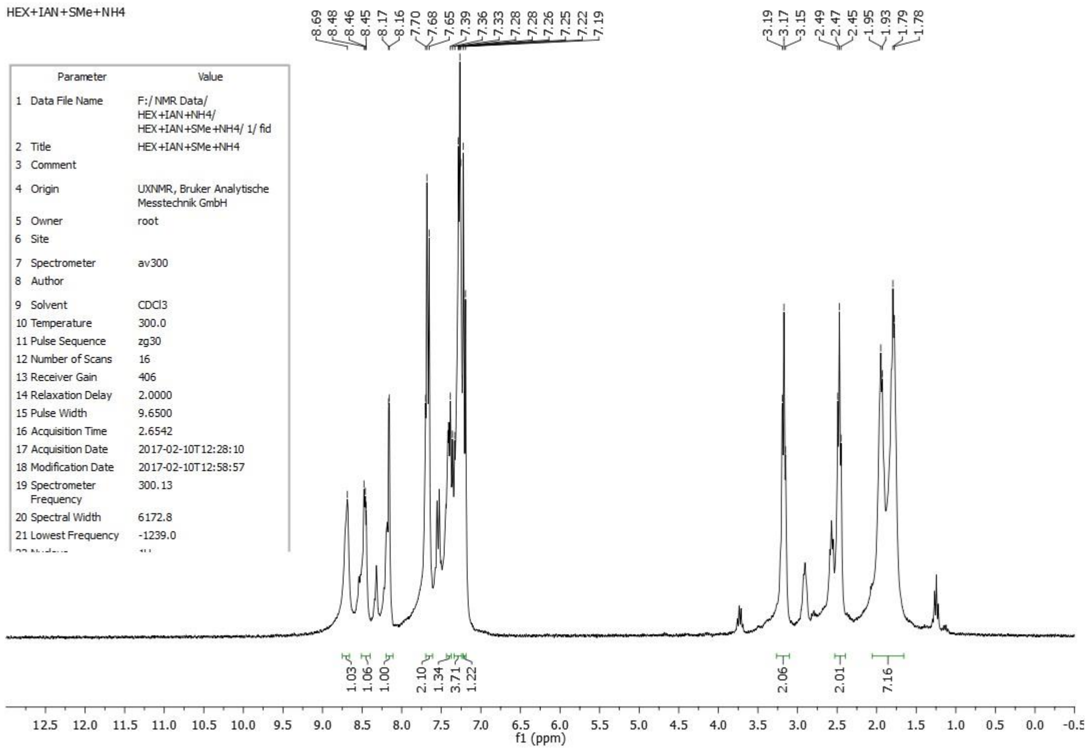

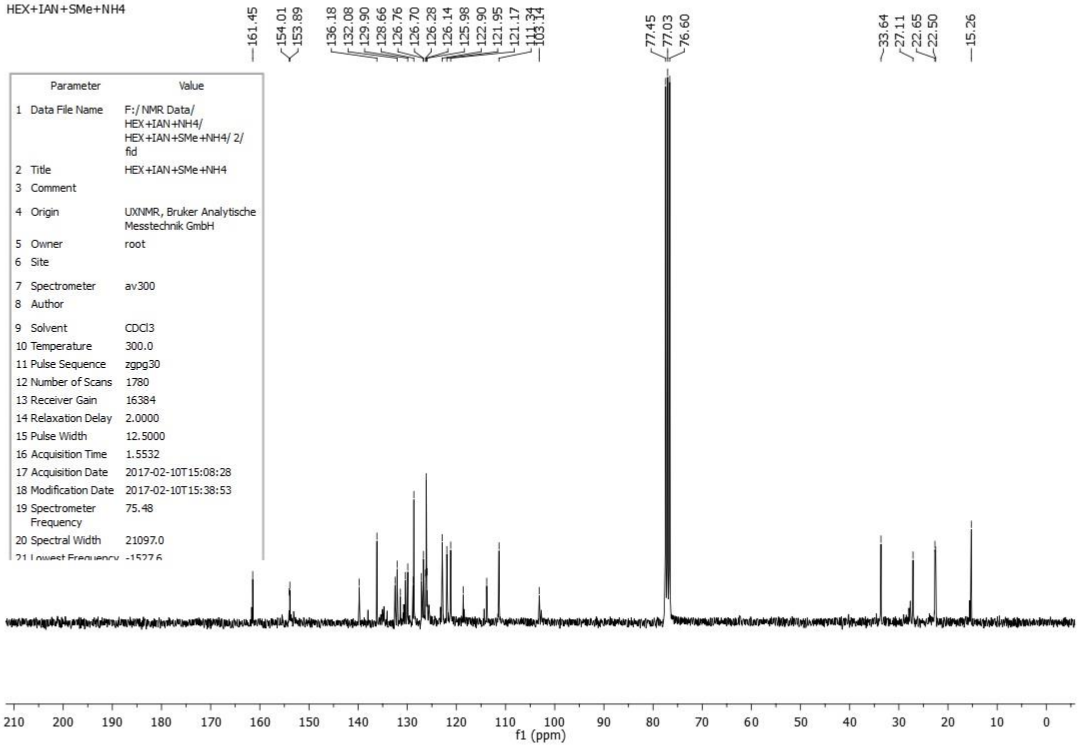

Figure 64: ^{1}H and ^{13}C NMR spectra of **18v**

X-ray structure determination and refinement

X-ray diffraction intensity data were collected for compounds **12r** and **16f** on a Bruker Smart Apex II single crystal X-ray diffractometer equipped with graphite mono-chromated MoKα (λ=0.7103 Å) radiation and CCD detector. Crystals were cut to suitable size and mounted on a glass fiber using cyanoacrylate adhesive. The unit cell parameters were determined from 36 frames (0.5° ω and φ scans) from three different crystallographic zones and using the method of difference vectors. The intensity data were collected with an average four-fold redundancy per reflection and optimum resolutions of 0.75 Å. The intensity data collection, frames integration, Lorentz and polarization correction and decay correction were done using SAINT. Empirical absorption correction multi-scan was performed using SADABS program. The accuracy of the crystal structures was evidenced from the final residual R- and wR- factors and other parameters including estimated standard deviations in the values of bond length and bond angles, 'data-to-parameter' ratio, etc. Details of the crystal data, data collection and refinement of **12r** and **16f** are given in **Tables S1** and **S2**, respectively.

Table S1. Crystal data and structure refinement parameters of **12r**

Empirical formula	$C_{30}H_{31}F_2N_3$	
Formula weight	471.58	
Temperature	296(2) K	
Wavelength	0.71073 Å	
Crystal system	Monoclinic	
Space group	P 21/n	
Unit cell dimensions	a = 11.5752(7) Å	α= γ =90°
	b = 7.8182(4) Å	β= 101.362(2)°
	c = 28.9629(17)Å	
Volume	2569.7(3)$Å^3$	
Z	4	
Density (calculated)	1.219 Mg/m^3	
Absorption coefficient	0.082 mm^{-1}	
F(000)	1000	
Crystal size	0.250 x 0.200 x 0.150 mm^3	
Theta range for data collection	3.1 to 28.7°.	
Index ranges	-15<=h<=15, -10<=k<=10, -39<=l<=39	
Reflections collected	53269	
Independent reflections	6872 [R(int) = 0.0697]	
Completeness to theta = 25.242°	99.2 %	
Refinement method	Full-matrix least-squares on F^2	
Data / restraints / parameters	6872 / 3 / 317	
Goodness-of-fit on F^2	1.328	
Final R indices [I>2sigma(I)]	R1 = 0.0735, wR2 = 0.1617	
R indices (all data)	R1 = 0.1440, wR2 = 0.1911	
Largest diff. peak and hole	0.356 and -0.254 e.$Å^{-3}$	

Table S2. Crystal data and structure refinement parameters of **16f**

Empirical formula	$C_{26} H_{21} Br FN_3$	
Formula weight	474.37	
Temperature	296(2) K	
Wavelength	0.71073 Å	
Crystal system	Monoclinic	
Space group	P 21/n	
Unit cell dimensions	a = 10.8550(1)Å	$\alpha = \gamma$ =90°
	b = 10.9522(1)Å	β= 93.141(3)°
	c = 18.533(2)Å	
Volume	2200.0(4)Å^3	
Z	4	
Density (calculated)	1.432 Mg/m^3	
Absorption coefficient	1.895 mm^{-1}	
F(000)	968	
Crystal size	0.270 x 0.230 x 0.190 mm^3	
Theta range for data collection	3 to 25°.	
Index ranges	-13<=h<=13, -13<=k<=13, -22<=l<=22	
Reflections collected	40938	
Independent reflections	40938 [R(int) = 0.0404]	
Completeness to theta	99.7 %	
Refinement method	Full-matrix least-squares on F^2	
Data / parameters	40938 / 280	
Goodness-of-fit on F^2	1.008	
Final R indices [I>2sigma(I)]	R1 = 0.0432, wR2 = 0.1200	
R indices (all data)	R1 = 0.0685, wR2 = 0.1416	
Largest diff. peak and hole	0.524 and -0.686 e.Å^{-3}	
